# Gene Gain and Loss during Evolution of Obligate Parasitism in the White Rust Pathogen of *Arabidopsis thaliana*

**DOI:** 10.1371/journal.pbio.1001094

**Published:** 2011-07-05

**Authors:** Eric Kemen, Anastasia Gardiner, Torsten Schultz-Larsen, Ariane C. Kemen, Alexi L. Balmuth, Alexandre Robert-Seilaniantz, Kate Bailey, Eric Holub, David J. Studholme, Dan MacLean, Jonathan D. G. Jones

**Affiliations:** 1 The Sainsbury Laboratory, Norwich Research Park, Norwich, United Kingdom; 2 The GenePool, The University of Edinburgh, Edinburgh, United Kingdom; 3 School of Life Sciences, University of Warwick, Wellesbourne Campus, United Kingdom; 4 School of Biosciences, University of Exeter, Exeter, United Kingdom; Massachusetts General Hospital, Harvard Medical School, United States of America

## Abstract

Biotrophic eukaryotic plant pathogens require a living host for their growth and form an intimate haustorial interface with parasitized cells. Evolution to biotrophy occurred independently in fungal rusts and powdery mildews, and in oomycete white rusts and downy mildews. Biotroph evolution and molecular mechanisms of biotrophy are poorly understood. It has been proposed, but not shown, that obligate biotrophy results from (i) reduced selection for maintenance of biosynthetic pathways and (ii) gain of mechanisms to evade host recognition or suppress host defence. Here we use Illumina sequencing to define the genome, transcriptome, and gene models for the obligate biotroph oomycete and *Arabidopsis* parasite, *Albugo laibachii*. *A. laibachii* is a member of the Chromalveolata, which incorporates Heterokonts (containing the oomycetes), Apicomplexa (which includes human parasites like *Plasmodium falciparum* and *Toxoplasma gondii*), and four other taxa. From comparisons with other oomycete plant pathogens and other chromalveolates, we reveal independent loss of molybdenum-cofactor-requiring enzymes in downy mildews, white rusts, and the malaria parasite *P. falciparum*. Biotrophy also requires “effectors” to suppress host defence; we reveal RXLR and Crinkler effectors shared with other oomycetes, and also discover and verify a novel class of effectors, the “CHXCs”, by showing effector delivery and effector functionality. Our findings suggest that evolution to progressively more intimate association between host and parasite results in reduced selection for retention of certain biosynthetic pathways, and particularly reduced selection for retention of molybdopterin-requiring biosynthetic pathways. These mechanisms are not only relevant to plant pathogenic oomycetes but also to human pathogens within the Chromalveolata.

## Author Summary

Plant pathogens that cannot grow except on their hosts are called obligate biotrophs. How such biotrophy evolves is poorly understood. In this study, we sequenced the genome of the obligate biotroph white rust pathogen (*Albugo laibachii*, Oomycota) of *Arabidopsis*. From comparisons with other oomycete plant pathogens, diatoms, and the human pathogen *Plasmodium falciparum*, we reveal a loss of important metabolic enzymes. We also reveal the appearance of defence-suppressing “effectors”, some carrying motifs known from other oomycete effectors, and discover and experimentally verify a novel class of effectors that share a CHXC motif within 50 amino acids of the signal peptide cleavage site. Obligate biotrophy involves an intimate association within host cells at the haustorial interface (where the parasite penetrates the host cell's cell wall), where nutrients are acquired from the host and effectors are delivered to the host. We found that *A. laibachii*, like *Hyaloperonospora arabidopsidis* and *Plasmodium falciparum*, lacks molybdopterin-requiring biosynthetic pathways, suggesting relaxed selection for retention of, or even selection against, this pathway. We propose that when defence suppression becomes sufficiently effective, hosts become such a reliable source of nutrients that a free-living phase can be lost. These mechanisms leading to obligate biotrophy and host specificity are relevant not only to plant pathogenic oomycetes but also to human pathogens.

## Introduction

For more than 150 years, attempts to culture downy mildews, powdery mildews, and rusts on artificial nutrient media have been unsuccessful. The terms obligate parasitism and obligate biotrophy are used to denote organisms that live in such an obligatory association with living hosts [1],[2]. Recent research on the obligate biotroph powdery mildew fungus *Blumeria graminis* or downy mildew oomycete *Hyaloperonospora arabidopsidis* reveals a close correlation between the biotrophic life style and massive gene losses in primary and secondary metabolism [3],[4]. Obligate biotrophs form an intimate haustorial interface with parasitized cells. Haustoria are differentiated intercellular hyphae, but little is known about their functionality and evolution beyond their involvement in nutrient uptake [5],[6].

The obligate biotroph oomycete *Albugo laibachii* is a member of the Chromalveolata, which incorporates Dinophyta, Ciliophora, Heterokonts (containing the oomycetes), Haptophyta, Cryptophyta, and Apicomplexa (which includes human parasites like *Plasmodium falciparum* and *Toxoplasma gondii* [7],[8]).

Within the oomycetes, *A. laibachii* belongs to a lineage known as peronosporalean, which includes the hemibiotrophic pathogen of potato *Phytophthora infestans* [9] and the necrotroph pathogen *Pythium ultimum* [10]. Within this lineage, obligate biotrophy evolved twice independently in white blister rusts (Albuginales) and downy mildews (part of the Peronosporaceae) [11]. The downy mildew pathogen *H. arabidopsidis* and *A. laibachii* are both pathogens of the model plant *Arabidopsis thaliana* [12]. While both show similar infection structures within the host [13],[14], *A. laibachii* releases motile zoospores from asexual spores and sexual oospores, while *H. arabidopsidis* lacks all motile stages [4],[15]. Both pathogens are regularly found to co-infect plants and sporulate on the same leaf [16].

A remarkable consequence of infection by *Albugo* sp. is enhanced host plant susceptibility to other parasites to which the host is resistant in the absence of *Albugo* infection, and also impairment of cell death mechanisms [16]. *Albugo* sp. infect 63 genera and 241 species [17], including economically important *Brassica rapa* (canola), *B. juncea* (oilseed mustard), and *B. oleracea* (cabbage family vegetables) [18],[19]. Recent analysis of oomycete evolutionary history [11] suggest that *Albugo* is more closely related to necrotrophs such as *Pythium* than to downy mildews, and thus provides a unique system to study the evolution and consequences of biotrophy, and to identify new defence-suppressing effectors and their host targets.

## Results/Discussion

### *A. laibachii* Isolates

Since prolonged culture of pathogen strains can result in genetic changes [20], we sequenced a fresh highly virulent field isolate of *A. laibachii*. The strain was selected from a heavily infected *Ar. thaliana* field plot (Norwich, United Kingdom) [21], and strains were single zoospore purified. Isolate Norwich 14 (Nc14) was determined as *A. laibachii* [19] and used for further analyses. In contrast to Nc14, *A. laibachii* isolate Em1 (formerly Acem1, *A. candida* East Malling 1 [19]) is an established *Albugo* strain that was collected 15 y ago [16],[22],[23], and we resequenced this strain. Both strains show identical ITS (internal transcribed spacer of ribosomal RNAs) and COX2 (cytochrome C oxidase subunit II) sequences. To ensure that sequence differences observed between these strains are of biological relevance not just the result of background mutations, we tested the host range for both isolates on 126 *Ar. thaliana* accessions and identified 12 that show resistance to only one of the *A. laibachii* isolates (Table S1). Nc14 is virulent on more accessions than the Em1 isolate is (Table 1).

**Table 1:** Percent of *Ar. thaliana* ecotypes resistant to *A. laibachii* Em1 and Nc14 isolates.

*A. laibachii* Isolate Tested	Percent Resistant *Ar. thaliana* Accessions		
	Per Each of the *A. laibachii* Isolates	To Both Isolates	Specifically to Only One of the Isolates
Em1	14.3	7.1	7.1
Nc14	9.5		2.4

Results indicate that the fresh isolate Nc14 is more virulent than Em1, which has been cultivated and propagated in the lab for more than 15 y.

### Illumina Genome Sequencing, Assembly, and Quality Assessment

**Figure 1:**
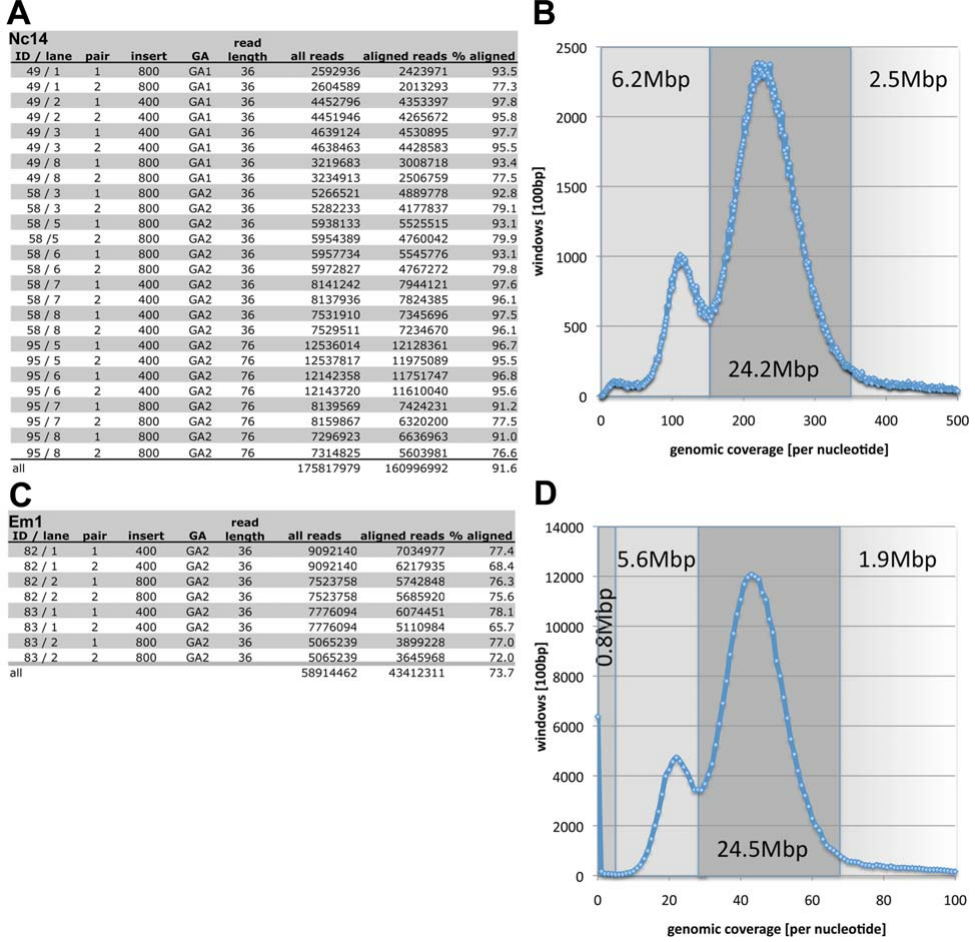
Genomic sequencing data and coverage of *A. laibachii* Nc14 and Em1 assemblies. (A) Reads generated for *A. laibachii* Nc14 using Illumina genome analyzer version 1 (GA1) or version 2 (GA2). (B) Distribution of genomic coverage. Grey fields indicate the total amount of sequence represented by the 100-bp windows with corresponding coverage. (C) Reads generated for *A. laibachii* Em1. (D) Distribution of genomic coverage showing Em1 reads aligned to the Nc14 genome using MAQ aligner. Nc14 and Em1 show a major peak at 226× and 43× coverage, respectively. A second peak is detected at 112× or 22×, showing half the coverage of the main peak, indicating highly heterozygous regions that were not merged in the assembly or hemizygous regions.

**Figure 2:**
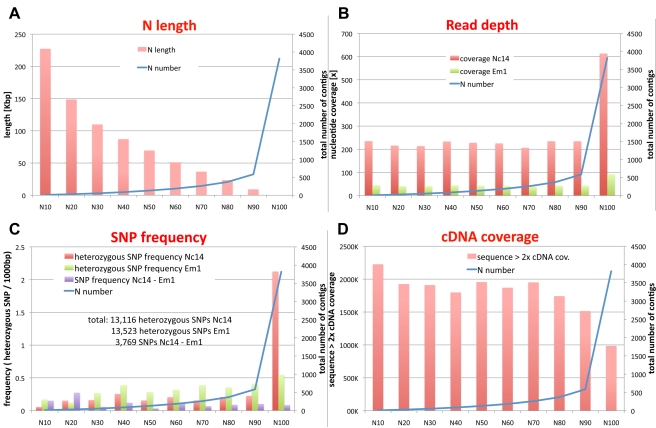
Distribution of contig length, nucleotide coverage, SNP frequency, and cDNA coverage in the *A. laibachii* assembly. (A) Genomic distribution of contig length (N length) versus contig number (N number). N lengths were calculated by ordering all sequences according to their length and then adding the length from longest to shortest until the summed length exceeded 10% (N10), 20% (N20), etc., up to 100% (N100) of the assembled contigs (32.7 Mbp). Blotting the N length versus the N number (number of contigs in each N category) indicates that 90% of the assembled genome show high continuity, while the last 10% are highly fragmented. (B) Average coverage for each category for Nc14 (red) and Em1 (green). In all, 90% of the genome shows low variation, consistent with 210–240× coverage for Nc14 and 40–50× for Em1. The last 10% show highly elevated coverage, indicating unresolved highly repetitive regions present in Nc14 and Em1. (C) Distribution of heterozygosity in each N category shows elevated levels in the set of short contigs. Heterozygous positions were accepted only if coverage was >180× and <350× for Nc14 (red) or >27× and <80× for Em1 (green). SNPs between Nc14 and Em1 were calculated ignoring heterozygous positions (lilac). (D) Alignment of Nc14 cDNA and summing up all regions showing >2× coverage indicate that the more continuous part of the genome contains more transcribed regions than the highly repetitive regions of the genome (in the histogram, N length and N number are cumulative while read depth, SNP frequency, and cDNA coverage are presented as binned data).

The *A. laibachii* Nc14 genome was sequenced using Illumina 76-bp paired reads with ∼240-fold coverage (Figure 1). In order to assemble the diploid heterozygous genome, an assembly pipeline was developed using Velvet [24] as primary assembler and Minimus [25] as meta-assembler (Figure S1). Short read assembly programs are sensitive to heterozygous positions depending on read depth and kmer-length. Reads not aligning to bacterial or plant sequence in public databases were used to estimate the genome size as ∼37 Mbp. Using the estimated genome size, 50% of the resulting assembly is contained in 164 contigs with an N50 of 56.5 kbp. A comparative analysis of contig size classes versus frequency indicates that 90% of the assembled genome shows a high degree of continuity in only 585 contigs, while 10% of the genome is fragmented in 3,231 contigs (Figure 2A). Read depth indicates that this 10% of the genome shows elevated levels of nucleotide coverage that are likely to comprise unresolved repeats (Figure 2B). Aligning Illumina cDNA reads from different stages of infection to reveal transcriptionally active regions in the assembly shows that few transcripts arise from the unresolved repetitive regions of the genome (Figure 2D), suggesting that the gene space of a genome can be reliably defined using Illumina-only approaches. A CEGMA [26] analysis revealed a high degree of completeness of assembly of core eukaryotic genes, as well as a continuity within the core genes comparable to high-quality Sanger read assemblies (Figure S2; Table S2). We designed 32 primer pairs for regions between 0.6 and 5 kb based on our assembly (Table S3). Thirty-one genomic regions could be amplified and were Sanger sequenced from both ends. All PCR products had the predicted size, and sequences showed 100% identity to the genome assembly.

The mitochondrial draft genome was assembled in a separate attempt because of its high repeat content and therefore higher coverage compared to the core genome. The assembled genome comprises 26.7 kb in 11 contigs and shows a high degree of synteny to the *P. infestans* mitochondrion Ia [27] and the *Py. ultimum* mitochondrion [10] (Figure S3). Considering the node coverage of the Velvet primary assembly (∼150×), 15.6 kb of the mitochondrial genome have >300× node coverage and seem to be duplicated. This might indicate, comparable to the *Py. ultimum* mitochondrion genome [10], that ∼50% of the genome is duplicated, leading to an estimated genome size of ∼43 kb. While the highly repetitive tRNAs are not resolved within the *A. laibachii* mitochondrial genome, regions of high synteny between the *Py. ultimum* and the *P. infestans* mitochondrial genome are found in ribosomal proteins and subunits of the NADH dehydrogenase as well as cytochrome C oxidase.

### Features of the *A. laibachii* Nuclear Genome

**Figure 3:**
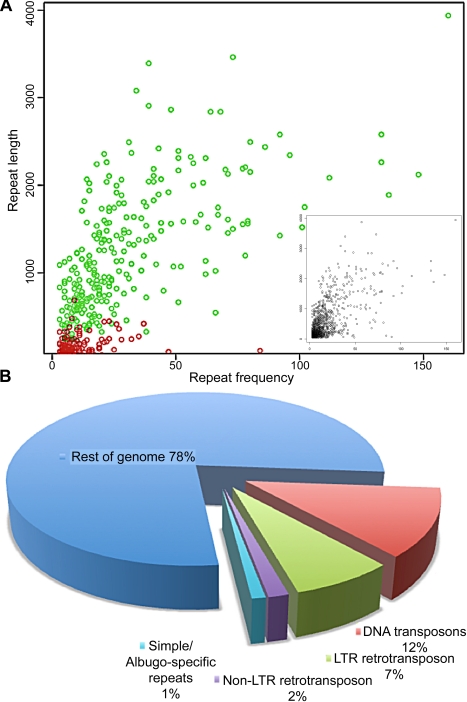
Repeats identified in the *A. laibachii* Nc14 contigs. Initial run of RepeatScout produced a library of 1,252 consensus repetitive sequences that include transposable elements, recently duplicated paralogous genes, and other dispersed duplicated regions. (A) Inset: The distribution of lengths of the identified repeats versus their frequency in the genome is shown. The majority of repeats fell into the category of short and rare in the assembly. The primary plot in (A) shows that the majority of the longest and most frequent repeats in the genome are transposon elements (shown in green and Table 1), while *Albugo*-specific repetitive sequences are mostly short (shown in red). (B) Summary of the proportion of the repetitive sequences (percent) in the *A. laibachii* Nc14 genome.

Approximately 22% of the *A. laibachii* Nc14 genome assembly consists of repetitive regions (Figure 3; Tables S4 and S5). The majority of repeats are represented by transposable elements (96%), while 4% of all repeats are *A. laibachii*-specific (Table S5). Compared to other obligate biotrophs, the number of repeats is low. *H. arabidopsidis*, for example, with an estimated genome size of 100 Mb, contains ∼43.3% repeats [4], while transposable elements account for 64% of the ∼120-Mb *Bl. graminis* (powdery mildew) genome [3]. We identified 45 contigs carrying telomeric repeats; amongst these, 25 contigs have telomeric repeats located at one end of a contig. We therefore postulate that the *A. laibachii* Nc14 genome is distributed over 12 or 13 chromosomes (Table S6). tRNA genes are difficult to resolve because of their high copy number [28]. Within our Illumina assembly, 153 tRNA genes were detected with 48 distinct anticodons (Figure S4; Table S7). Our ability to resolve all these repeats within the Illumina short read assembly illustrates its quality.

Based on read depth, both Nc14 and Em1 isolates possess ∼6 Mbp of hemizygous or highly heterozygous regions (6.2 and 5.6 Mbp for Nc14 and Em1, respectively) (Figure 1B and 1D) as well as ∼13,000 heterozygous loci (13,116 and 13,523 for Nc14 and Em1, respectively) (Figure 2C). Remarkably, most of the hemizygous/highly heterozygous regions are shared between Nc14 and Em1.

**Figure 4:**
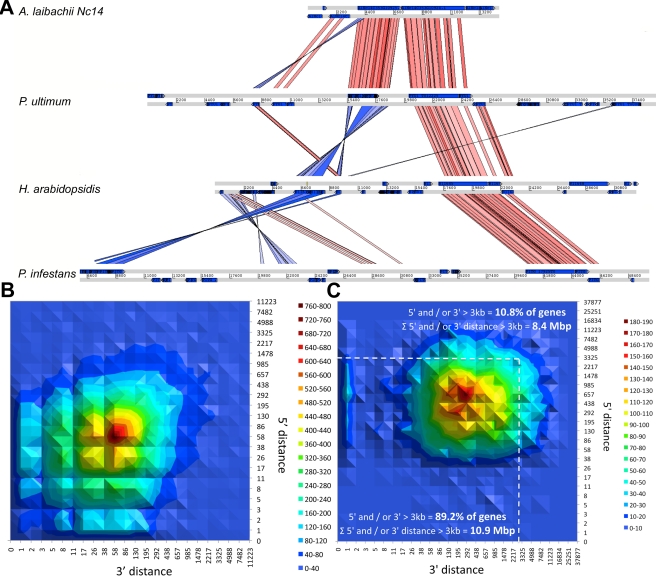
*A. laibachii* has a compact genome with expression clusters. (A) Synteny between *A. laibachii*, *Py. ultimum*, *H. arabidopsidis*, and *P. infestans*. The region shown is an example of the dense clustering of genes in the pentafunctional AROM polypeptide and a P-type ATPase. The AROM polypeptide comprises five enzymes of the shikimate pathway in one enzyme. With increasing genome size the distance between both genes increases and re-organisations occur (red, synteny without inversion; blue, inverted regions). (B) Plotting the distance between transcriptional islands based on the 5′ to 3′ orientation of the forward strand reveals that transcriptional regions are clustered close together. The maximum peak reflects the average intron size. Regions with no 3′ but with 5′ distance and vice versa reflect overlapping 3′ and 5′ non-coding regions of genes. Analysing the distance distribution between transcriptional units reveals a median distance between genes of 45 bp, showing that within transcribed regions, nearly all the DNA sequence corresponds to genes. (C) Plotting the 5′–3′ distance for all genes from ATG to stop to the next gene confirms the gene clustering. Only 10.8% of all genes have a distance to the next gene or the end of the contig greater than 3 kb. Summing the distance between these genes contributes to only 10.9 Mbp of the genome because of the close packaging, while summing the distance of the few genes that are not in clusters contributes to 8.4 Mbp of the genome.

Compared to other sequenced oomycetes like *P. infestans* (240 Mbp), *H. arabidopsidis* (100 Mbp), or even *Py. ultimum* (42.8 Mbp), *A. laibachii* has a highly compact genome structure (Figure 4A). Approximately 50% of the *A. laibachii* genome assembly matched cDNA reads, and transcriptionally active regions are further clustered, resulting in transcriptional hot spots and silent genomic regions (Figure 4B).

### Annotation and Validation of Protein Coding Genes

A reference set of 13,032 gene models was generated incorporating cDNA reads from different stages of infection (Figure S5A). From extensive cDNA sequencing of infected *Arabidopsis* leaves, approximately 20 M (∼1.5 Gbp) unique Illumina reads match the Nc14 genome assembly but not *Ar. thaliana* TAIR 9.0, and these were used to generate training sets for *ab initio* gene predictions and as evidence sets for consensus gene prediction. In all, 88.3% of all gene models are supported by at least three cDNA hits.

For validation of these gene models, a set of 860 annotated core eukaryotic orthologous groups (KOGs) [29] was compiled and tested. In all, 75% of these groups are present in the current annotation. For comparison, 78% of KOGs were present in *P. infestans*, 73% in *H. arabidopsidis*, 42% in *Pl. falciparum*, and 85% in *Ar. thaliana* (Figure S5B). In addition, 49.9% of all gene models show Pfam support, resulting in 2,505 Pfam domains, and 803 genes were functionally assigned to pathways using ASGARD [30] and manual annotation. Transcriptional units show an even more compact, clustered occurrence than *P. sojae* or *P. ramorum* and an occurrence pattern clearly different from that of *P. infestans* [9] (Figure 4C).

From our annotations using ASGARD we identified major enzymes of the lipopolysaccharide biosynthesis pathway, as have been described for *P. infestans* [31]. These analyses revealed, in addition, the possibility that *A. laibachii* is able to synthesize brassinosteroids. We identified potential homologues to the *Ar. thaliana* brassinosteroid biosynthesis genes *Dwf4* and *DET2* (Table S8). Although ASGARD identified homologues of *Br6ox*, *D2*, and *CPD*, manual annotation revealed that assigning function to members of the superfamily of cytochrome P450 enzymes in *A. laibachii* is difficult based on homology alone (Table S8). It has been hypothesized that the frequency of functionally redundant genes is reduced in obligate biotrophs, as reported for *Bl. graminis* [3]. Combining ASGARD and manual annotation we identified the absence of the whole steroid biosynthesis pathway, and, like other oomycetes, *A. laibachii* probably relies on the host as a source of sterols. We hypothesize that *A. laibachii* would need to take up campesterol from the plant as a precursor for brassinosteroid synthesis.

### Ancestral Red and Green Algae Genes in the *A. laibachii* Genome

During evolution, plastids of both red algae and green algae were transferred to other lineages by secondary endosymbiosis. How often and when secondary endosymbiosis occurred is difficult to address but of importance to clarify the origin of chromalveolates and their gain and loss of endosymbionts. There are two distinct hypotheses for what took place. The monophyletic hypothesis posits that a red alga was taken up only once, followed by repeated losses of this algal genome, giving rise to the highly divergent group of chromalveolates [32]. An alternative and more common view hypothesizes polyphyletic origins of the Chromalveolata, with in some cases multiple events of secondary endosymbiosis [33]–[35].

**Figure 5:**
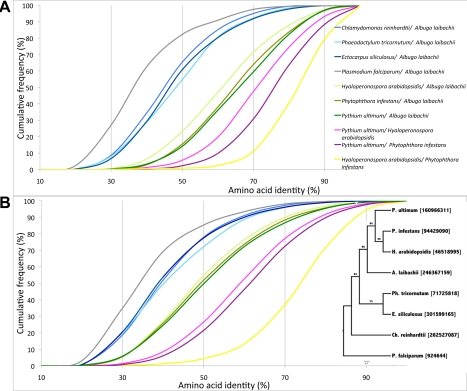
Molecular divergence between *A. laibachii* and other species based on pairwise comparisons. (A) Molecular divergence based on all pairwise comparisons of the one-to-one orthologues. In the figure, the cumulative frequencies of amino acid identity across each set of potential orthologous pairs is presented, indicating that although *H. arabidopsidis* and *A. laibachii* are both biotrophs, *H. arabidopsidis* is less diverged from *P. infestans* than it is from *A. laibachii* (e.g., in the *H. arabidopsidis*–*A. laibachii* comparison, ∼22% of all orthologues show an amino acid identity of <50%, while only ∼14% in a *Py. ultimum*–*A. laibachii* comparison show an amino acid identity of <50%). *A. laibachii* shows the highest amino acid identity to *Py. ultimum*. (B) Molecular divergence between *A. laibachii* and other species based on the subset of core eukaryotic genes to show stability of the test. Results are consistent with the one-to-one orthologue analyses although differences between *A. laibachii*, *P. infestans*, *H. arabidopsidis*, and *Py. ultimum* are less obvious, indicating the lack of selection pressure on the core eukaryotic genes [37]. For comparative reasons, a tree using ITS2 sequences is added. The represented tree is a maximum likelihood tree produced with PhyML.

Molecular divergence of *A. laibachii* from other species within the Chromalveolata was assessed by examining the percentage of amino acid identity between orthologous gene pairs (Figure 5). These analyses demonstrate that the green alga *Chlamydomonas reinhardtii*, the brown alga *Ectocarpus siliculosus*, and the diatom *Phaeodactylum tricornutum* show the same distribution of percentage amino acid identity to *A. laibachii* Nc14 regarding the cumulative frequency of orthologous pairs. In contrast, previous systematic analyses suggested that brown algae and diatoms are the closest relatives of oomycetes and that secondary endosymbiosis occurred with a red alga [32], although there are suggestions that oomycetes diverged before this event [36]. Using a set of >1,700 genes that are of “green” origin (from green algae) or “red” origin (from red algae) and that have been integrated into the diatom nuclear genome [37], we found more oomycete genes that show significant BLAST hits to green algae than to red algae (34 “green” compared to five “red”) (Figure S6; Table S9). These findings are consistent with the results published by Moustafa et al. [37] for diatoms. In a separate approach we identified genes showing high similarity between oomycetes, green algae, and red algae that are absent from diatoms (32 “green”; 11 “red”) (Tables S10 and S11). This result might indicate the presence of all these genes in a common ancestor, followed by loss or expansion of the gene family depending on adopted live style. To address this question, we further analysed genes absent from *A. laibachii* Nc14 and studied their presence/absence in three other oomycetes, *Pl. falciparum*, and the brown alga *E. siliculosus* (Table S12). The majority of genes absent from *A. laibachii* Nc14 are absent from other oomycetes and from *Pl. falciparum* but are present in the brown alga. These genes are involved in the photoautotrophic, aquatic life style of diatoms and algae, such as a sodium/bile acid cotransporter, a haloacid dehalogenase-like hydrolase, fatty acid biosynthesis genes, a zeaxanthin epoxidase and a fucoxanthin chlorophyll a/c binding protein. In contrast to the genes lost, we found that certain gene families like aspartic proteases or proteases containing MORN (membrane occupation and recognition nexus) repeats [38] show expansion in *A. laibachii* Nc14 compared to in diatoms. Although our results fit the hypothesis of a common ancestor, we cannot exclude horizontal gene transfer and uptake of an endosymbiont after the divergence between a brown algal ancestor and an oomycete ancestor, given the low number of diagnosed genes that we could analyse.

Potentially green-algae-derived proteins carrying MORN repeat domains (Figure S7) are involved in the complex process of internal budding in apicomplexans [39], which may be similar to the zoospore formation of oomycetes within oospores or zoosporangia or gamete formation in diatoms [40]. While oomycetes with a motile zoospore stage like *A. laibachii* and *P. infestans* carry the MORN repeat proteins, these proteins are absent in the non-motile *H. arabidopsidis* and absent in the non-motile red alga *Cyanidioschyzon merolae* [41]. We therefore hypothesize that loss of this gene of hypothetical green algal origin could have led to the evolutionary loss of the whole flagellum apparatus in *H. arabidopsidis* [4]. However, we cannot rule out that depletion of any major flagellar protein could have caused evolutionary loss of the whole flagellum apparatus. Inspection of the flagellar inner arm dynein 1 heavy chain alpha, which is absolutely necessary for flagellum function, reveals that genomic regions carrying flagellar inner arm dynein 1 heavy chain alpha genes show a high degree of synteny between oomycetes like *Py. ultimum* and *A. laibachii*. In contrast, a syntenic region in *H. arabidopsidis* shows replacement of the flagellar dynein by Mariner- or Gypsy-like transposable elements (Figure S8).

### Comparative Genomics to Identify Genes Implicated in Biotrophy

Since within the peronosporalean lineage, biotrophy evolved twice independently [11], we compared *A. laibachii* with the other obligate biotroph *H. arabidopsidis* [4], hemibiotroph *P. infestans* [9], and necrotroph *Py. ultimum* [10] (Figure 5; Tables S13 and S14). We found that *H. arabidopsidis* is the most diverged from *A. laibachii*. *H. arabidopsidis* shares the fewest (4,826) orthologous genes with *A. laibachii*, versus the average of 5,722 in *A. laibachii*/*P. infestans* and *A. laibachii*/*Py. ultimum* comparisons. Meanwhile, *H. arabidopsidis* genes show the highest amino acid identity with the genes of *P. infestans*, on average 73% of amino acid identity between all single copy orthologous pairs.

*Py. ultimum* shares the highest number of orthologous genes with *A. laibachii* (5,910 pairs). *P. ultimum* proteins also have a slightly higher percentage of amino acid identity with *A. laibachii* proteins than with other oomycetes (Figure 5). Yet, *Py. ultimum* itself is closer to *H. arabidopsidis* and *P. infestans* than to *A. laibachii*, sharing with them more orthologous genes with higher mean amino acid identity.

These analyses support the hypothesis that *A. laibachii* and *H. arabidopsidis* evolved biotrophy independently; genes missing in one or the other genome compared to the necrotroph *Py. ultimum* or hemibiotroph *P. infestans* may be correlated with biotrophy (Table S15). One of these genes is that for molybdenum-cofactor-dependent nitrate reductase. Nitrate reductase catalyzes pyridine-nucleotide-dependent nitrate reduction for nitrogen acquisition [42]. Both biotroph pathogens have a set of transporters showing homology to amino acid transporters, but other uptake mechanisms or sources could also enable nitrogen acquisition from their hosts [43]. While *H. arabidopsidis* lost only the nitrate reductase, *A. laibachii* also lost the sulphite oxidase and the whole molybdopterin (a cofactor required for nitrate reductase and sulphite oxidase function) biosynthesis pathway. In *Pl. falciparum*, which shows a high degree of adaptation to parasitism, nitrate reductase, sulphite oxidase, and the whole molybdopterin biosynthesis pathway are also missing. Most likely the loss of the two Mo-containing enzymes and the Mo-cofactor biosynthesis is the outcome of biotrophy and not the reason for biotrophy, though conceivably there may have been selection against this pathway if other nitrogen or sulphate sources are less energy-consuming and therefore enhance fitness during parasitism. Molybdenum has been reported to interfere with function of chaperones like Hsp90 [44],[45]. Avoiding the uptake of molybdenum might prevent this Hsp90 inhibition and increase fitness on *Ar. thaliana* accessions with high molybdenum levels like Col-0 [46]. *H. arabidopsidis* therefore could be in a less advanced stage of host adaptation compared to *A. laibachii* and *Pl. falciparum*.

Besides biotrophy, the formation of haustoria and haustorium-like structures evolved several times in peronosporalean biotroph and hemibiotroph pathogens. Haustoria in fungi are sites of enhanced nutrient uptake [47] and metabolism, such as thiamine biosynthesis [48]. In the oomycetes, all haustorium-forming species have lost the thiamine biosynthetic pathway. We infer that haustorial oomycetes obtain thiamine from the host.

We therefore hypothesize that evolution to biotrophy is initiated not by gene loss, but rather from the ability to build a haustorium and therefore differentiate a sophisticated interface with a host. The critical step to adopting biotrophy is likely to be efficient defence suppression to enable persistence of functioning haustoria; subsequent loss of biosynthetic pathways is likely to be secondary.

### The *A. laibachii* Secretome

Well-adapted human pathogens like *Pl. falciparum* and plant pathogenic fungi like *Ustilago maydis* have small secretomes (320 [49] and 426 [50] proteins, respectively) compared to necrotrophic fungi like *Aspergillus fumigatus* (up to 881 proteins [51]). We found that the same is true for oomycetes. Using SignalP [52] to predict potential secretion signal peptides and MEMSAT [53] to predict transmembrane (TM) domains, we identified 2,473 (2,136 without TM domains) potentially secreted proteins in the hemibiotroph *P. infestans* and 1,636 (1,222 without TM domains) in the necrotroph *Py. ultimum*. For *H. arabidopsidis* only 1,350 (1,054 without TM domains) and for *A. laibachii* 949 (672 without TM domains) were identified. Analysing the secretome for pathogenicity-related proteins like proteases, glucosyl hydrolases, and potential elicitins or lectins reveals a significant reduction in the *H. arabidopsidis* and *A. laibachii* secretome (Tables 2 and S16). We postulate that biotrophs reduce their activation of host defence by reducing their inventory of secreted proteins, particularly cell wall hydrolyzing enzymes.

**Table 2:** Quantitative comparison of pathogenicity-related proteins.

Protein	*A. laibachii* (Secreted Only)	*A. laibachii* (All)	*H. arabidopsidis* (All)	*P. infestans*	*Py. ultimum*	*T. pseudonana*	*Pl. falciparum*
Aspartyl proteases	1	10	9	12*	22**	5	11
Serine carboxypeptidases	6	32**	6	24*	1	6	0
Cysteine proteases	16	16	7	33**	32*	16	4
Glycosyl hydrolases	15	44	66	157**	85*	31	1
Pectin esterases	0	0	4*	11**	0	0	0
Pectate lyases	0	1	8	30**	15*	0	0
Cutinase	2*	2*	2*	4**	0	0	0
Lipases	3	12	10	19*	19*	22**	9
Phospholipases	3	13	13	36**	6	18*	15
Protease inhibitors, all	0	0	3	38**	1	11*	1
Cytochrome P450s	1	3	16	19*	39**	7	17
ABC transporters	3	41	53	156*	173**	50	10
NPP1-like proteins (necrosis-inducing proteins)	0	0	24*	27**	7	0	0
Elicitin-like proteins	1	3	1	40**	7*	0	0
Lectin-like proteins	5	6	6	10*	20**	0	0
Crinklers (CRN family) candidates	2	3	20	196**	26*	0	0
RXLR/Q effector candidates	49	49	115*	505**	57	62	4
CHXC effector candidates	29**	29**	3	5*	4	2	1

Genes were predicted for all datasets using Pfam prediction and BLASTP against NCBI data or specific datasets of selected protein groups. Results were further compared to data published by Haas et al. for *P. infestans* [9] or Levesque et al. for *Py. ultimum* [10], or Baxter et al. for *H. arabidopsidis* [4]. The data indicate that the *P. infestans* secretome of pathogenicity-related proteins is bigger than that of all other compared and annotated genomes (^**^, highest number; ^*^, second highest number).

### The *A. laibachii* Effector Complement

**Figure 6:**
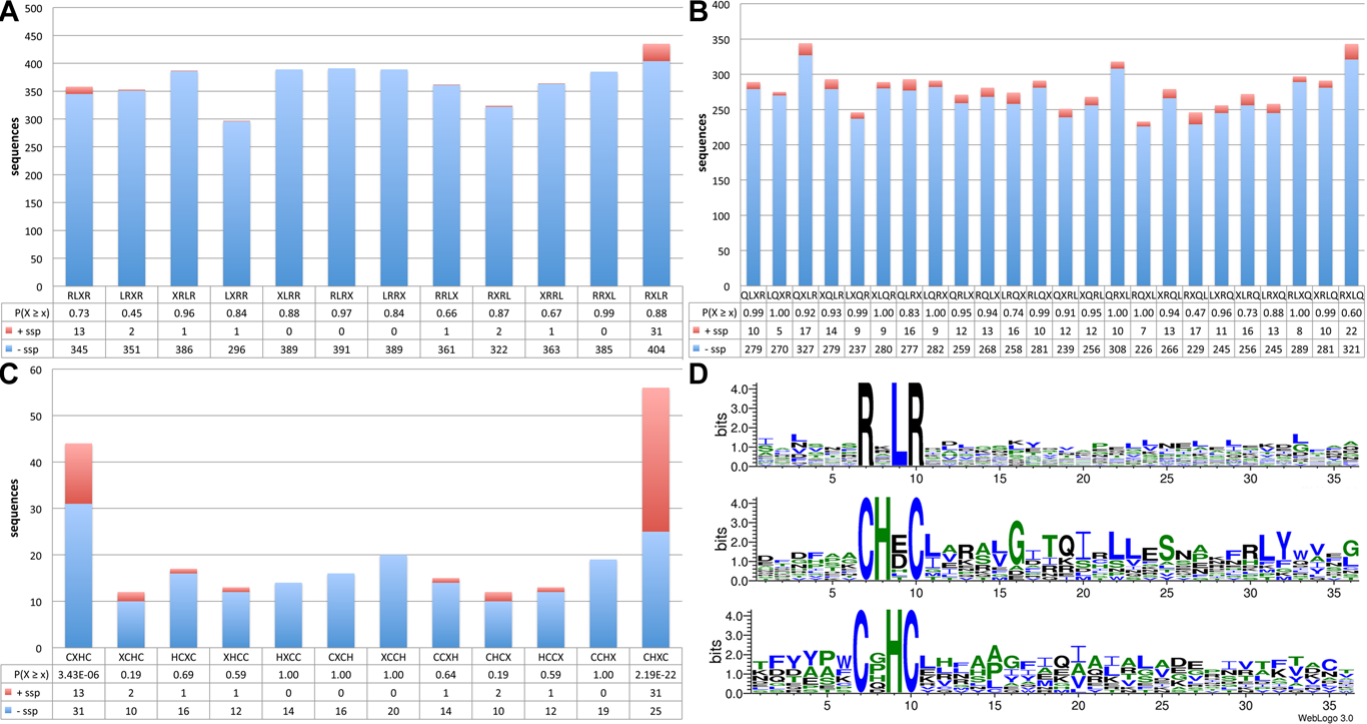
Validation and identification of potential delivery motifs. To identify potential effector delivery motifs we analysed RXLR (A), RXLQ (B), and the new CHXC (C) effector candidates for enrichment in the secretome. Motif shuffling was used to identify background levels. Calculating the cumulative hypergeometric probability to analyse the enrichment of secreted proteins (red) over non-secreted proteins (blue) for each of the permutated motifs reveals a significant enrichment of CHXCs in the secretome (*p*[*X*≥*x*] = 2.19×10^−22^). None of the RXLR or RXLQ motifs or permutations shows significant enrichment. There is also enrichment for CXHC proteins in the secretome. Except for one CHHC protein, there is no overlap between the two motif classes. The logo blot (D) clearly indicates that RXLR-containing proteins are conserved only within the selected amino acids, while for CHXCs it is not only the motif but also sequences C-terminal to it are conserved, including conserved glycine, leucines, and a tyrosine.

The ability to establish a sophisticated zone of interaction like the parasitophorous vacuole in *Pl. falciparum* or the haustorium in oomycetes and fungi requires sophisticated host defence suppression [54], which is predominantly achieved via secreted proteins delivered into the host cell [55],[56]. The *A. laibachii* secretome comprises 672 secreted proteins without TM domains. Genetically identified oomycete avirulence (Avr) proteins are secreted proteins that have signal peptide and RXLR motifs [57],[58]. In many oomycete genomes the RXLR motif is over-represented and positionally constrained within the secreted protein [59]. We identified 25 RXLR and 24 RXLQ effector candidates in the *A. laibachii* secretome. To determine the likelihood that RXLR or RXLQ motifs occur merely by chance in the *A. laibachii* secretome based on amino acid content, we performed *in silico* permutation of the motifs (Figure 6A and 6B). We concluded that the RXLR and RXLQ motifs were not likely to occur merely by chance, and that the likelihood of occurrence by chance is higher in the proteome as a whole than among secreted proteins. It was shown for *P. infestans* that effectors are often located in gene-depleted repetitive regions of the genome [9]. We therefore investigated RXLR candidate proteins in highly repetitive regions of the genome. We identified two RXLRs, one in a highly conserved repeat region with ∼10 repeats in Nc14 and one in a more diverged repeat region with >80 repeats within the genome. The first region also exists in *A. laibachii* isolate Em1; the diverged repeat of the second identified region exists but without the RXLR gene-containing region (Figure S9). There are 563 RXLR effector candidates identified in *P. infestans* [9], so RXLR effectors are less likely to be relevant for *A. laibachii* virulence.

Similar conclusions can be drawn for the CRN protein family, which shows expansion in *P. infestans* [9],[60] but not *A. laibachii*, where only three members of the CRN family could be identified with signal peptides. Eight additional CRN-like proteins were identified where no signal peptide has been predicted.

To identify new classes of effectors in the Albuginales clade, the secretome of *A. laibachii* was computationally screened for genes either showing heterozygosity or showing nucleotide polymorphisms between Nc14 and Em1. We identified a new class carrying a “CHXC” motif by inspection of the first 80 amino acids after the signal peptide cleavage site. CHXC candidates are significantly enriched within the secretome (Figure 6C). Comparisons of the N-terminal part of the CHXC proteins revealed additional conserved amino acids, particularly a glycin at +6 to the CHXC motif (Figure 6D).

### Intraspecies Comparison between *A. laibachii* Nc14 and *A. laibachii* Em1

In host–pathogen interactions, intraspecies comparisons enable the search for virulence alleles that undergo positive selection and fixation within the population [61],[62]. Secreted proteins with close contact to the host cell, such as effector proteins, often show enhanced levels of positive selection [63],[64]. By comparing the two *A. laibachii* isolates Nc14 and Em1, we identified a significantly higher frequency of non-synonymous to synonymous mutations within the predicted secretome compared to the rest of the proteome. Our analyses showed that this was particularly true for heterozygous positions and less convincing for homozygous SNPs (Table S17). Genes that are highly conserved between species, like KOGs, showed comparable non-synonymous and synonymous substitution rates, with a slight excess of synonymous mutations. There are significantly more genes within the KOGs showing a non-synonymous/synonymous ratio less than 1 than genes with values greater than 1. Comparing this to candidate effector classes like RXLRs, RXLQs, and CHXCs reveals that in particular the CHXCs show significantly higher frequencies of non-synonymous to synonymous mutations. This supports the idea that the CHXC sub-class of secreted proteins is under positive selection, similar to other described oomycete effectors like ATR1 or ATR13 from *H. arabidopsidis* [57],[65].

Further to this we identified Nc14 genes absent or highly diverged from the Em1 complement. We defined a gene as absent or highly diverged if >10 bp showed 0 coverage in the Em1 alignment. Out of the 672 secreted proteins without TM domains, we identified seven as absent from Em1 (1.04%). We also detected two with a predicted TM domain (0.73%) that are absent from Em1. Regarding all gene models, 96 were absent (0.74%). This finding is a further indication for a greater selection pressure on secreted than on non-secreted proteins, as has been found in species or interspecies comparisons in *Phytophthora* sp. [66] and *Ustilago/Sporisorium* [67].

### Validation of Effector Delivery

**Figure 7:**
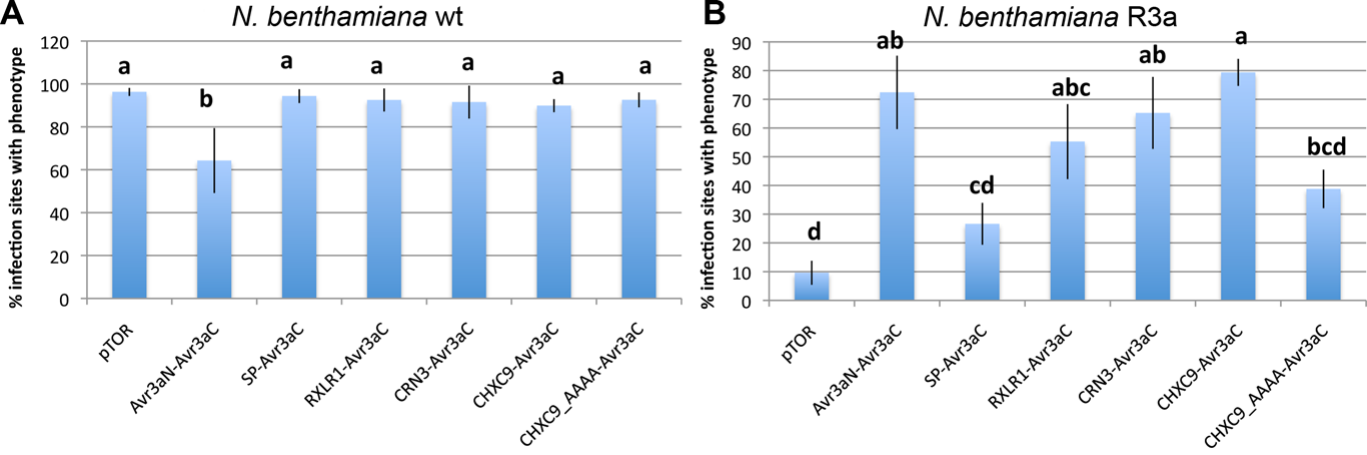
*P. capsici* test for delivery motif. To identify known and new classes of transfer motifs in *A. laibachii*, the *P. capsici–N. benthamiana* translocation assay was used. This test system is based on Avr3a-mediated avirulence in plants carrying R3a [68]. (A) virulence assay to show that transgenic *P. capsici* is not impaired in growth; (B) Hypersensitive response (HR) assay on R3a-carrying plants for delivery assay. The Avr3a RxLR translocation domain is replaced by the N-terminus of different *A. laibachii* effector candidates. For the assay, RXLR1, CRN3, and CHXC9 carrying the newly identified CHXC motif were used. Our results validate that known motifs like the CRN motif are functional while the selected RXLR shows low delivery efficiency. CHXC9 shows the same efficiency as Avr3a does, and dependency of the CHXC motif could be identified (statistical analyses using the Tukey test; means with the same letter are not significantly different; error bars denote standard error of the mean). wt, wild type.

We tested *A. laibachii* effector candidates (one CHXC, one RXLR, and one CRN effector candidate) for their host delivery efficiency using a *P. capsici*–*Nicotiana benthamiana* translocation assay [68]. Briefly, N-terminal domains of candidate effectors were fused to the *P. infestans* Avr3a effector domain, transformed into *P. capsici*, and tested for whether they confer translocation of Avr3a into *N. benthamiana* carrying *R3a*, resulting in avirulence. Statistical analyses of the delivery efficiency (Figure 7) clearly indicate that the *A. laibachii* CRN3 N-terminus and CHXC9 N-terminus are as efficient as the Avr3a N-terminus in Avr3a translocation, while the RXLR1 N-terminal domain is less efficient. An alanine replacement construct of the CHXC motif supports the importance of this motif for delivery efficiency. The Avr3a C-terminus alone confers a low basal delivery level without the need for the N-terminal enhancer. These findings reveal the potential of the CHXC proteins to be delivered into the host cell, similar to RXLRs and CRNs, though the delivery mechanism for all these effector classes requires further investigation.

### Validation of Virulence-Conferring Function of *A. laibachii* Effector Candidates

**Figure 8:**
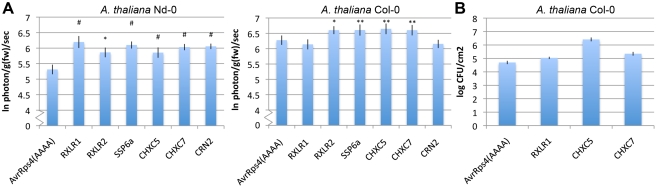
Candidate *A. laibachii* Nc14 effectors contribute to Pst DC3000 virulence. (A) *Arabidopsis* plants (4- to 5-wk-old) were spray inoculated with 5×10^8^ CFU Pst DC3000 lux harbouring candidate effectors cloned in pEDV6. Bacterial growth was measured as an increase in luciferase photon emission per gram fresh weight per second (photon/g[fw]/sec). The histogram represents the log median of photon emission of three independent experiments, each with four technical replicates. Error bars denote standard error of the mean. Two-way ANOVA: #, *p*<0.001; **, *p*<0.01; *, *p*<0.05 from AvrRps4(AAAA). (B) Plants 4- to 5-wk-old were infected with 5×10^8^ CFU of Pst DC3000 ΔAvrPto/ΔAvrPtoB harbouring candidate effector cloned in pEDV6. Bacterial populations were sampled 4 d post-inoculation. The histogram represents the median colony count of two independent experiments, each with more than four technical replicates. Error bars denote standard error of the mean.

To assay the effectors for virulence function, we used *Pseudomonas syringae* pv. tomato (Pst) DC3000 luciferase [69] carrying “effector detector vector” (EDV) constructs to deliver effectors into the plant cytoplasm via type III secretion [70] (Figure 8). Tests on *Ar. thaliana* Nd-0 plants revealed that several selected *A. laibachii* RXLRs, CRNs, and CHXCs enhance virulence compared to a non-functional AvrRps4 (AvrRps4[AAAA]). On *Ar. thaliana* Col-0, in contrast, the CRN and one RXLR (RXLR1) do not enhance virulence while RXLR2 and CHXCs still do. These tests indicate that CHXCs carry the capacity to enhance virulence in phytopathogenic bacteria, perhaps by suppression of host resistance mechanisms [54],[70]. These virulence assays together suggest that *A. laibachii* uses at least three different major effector classes.

### Evolutionary Origin of CHXC Effectors

To try to identify the evolutionary source of CHXCs, we investigated enrichment of CHXC-motif-containing proteins in the secretomes of *P. infestans*, *Py. ultimum*, *H. arabidopsidis*, *Saprolegnia parasitica*, *Thalassiosira pseudonana* (diatom), *Pl. falciparum* (Apicomplexa), *E. siliculosus* (brown alga), *C. merolae* (red alga), *Ch. reinhardtii* (green alga), *Volvox carteri* (green alga), and *Ar. thaliana*. Only *A. laibachii* contained a significant enrichment of CHXCs in its secretome. Although not significantly enriched, both the fish pathogen *S. parasitica* and the land plant *Ar. thaliana* contained more than ten CHXC proteins carrying potential secretion signals (14 and 11, respectively) (Figure S10). In contrast to CHXC-containing proteins, almost all inspected organisms show a high number of CXHC-containing potentially secreted proteins; a common CXHC protein is protein disulphide isomerase (Table S18).

**Figure 9:**
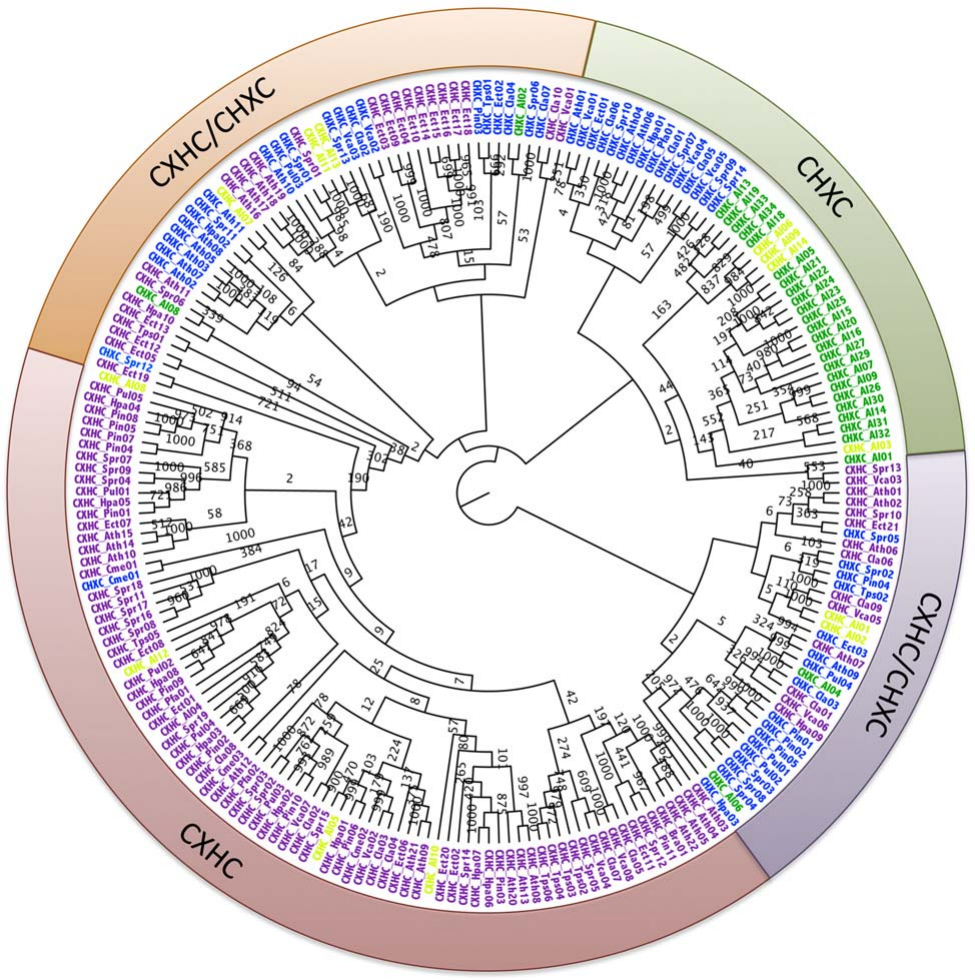
Result of neighbour-joining analyses using N-termini of all predicted CHXCs or CXHCs from the genomes of *P. infestans*, *Py. ultimum*, *H. arabidopsidis*, *T. pseudonana*, *Pl. falciparum*, *E. siliculosus*, *C. merolae*, *Ch. reinhardtii*, *V. carteri*, *S. parasitica*, as well as *Ar. thaliana*. The outer ring summarises clades with N-termini predominantly carrying CHXC or CXHC motif or mixed clades (CXHC/CHXC) into classes. *A. laibachii* CHXCs are mainly clustered in the CHXC class (green), containing besides *A. laibachii* distantly related CHXCs from *S. parasitica*, *V. carteri*, *Ch. reinhardtii*, and *Ar. thaliana*. CHXCs are distant from endoplasmic reticulum proteins like disulphide isomerases that predominantly carry the CXHC motif and are grouped within the CXHC class (red). Between the CHXC class and the CXHC class, mixed clades contain protease and defensin homologues (orange) or *Ar. thaliana* cystein-rich proteins (violet). (Names in green indicate *A. laibachii* CHXCs and in yellow, *A. laibachii* CXHCs. Blue indicates CHXCs from other species; magenta indicates CXHCs from other species; 16 amino acids before and 45 amino acids after the CHXC or CXHC motif in the N-terminus were used. The tree is midpoint rooted. All bootstrap counts refer to 1,000 replications.). Ath, *Ar. thaliana*; Cla, *Ch. reinhardtii*; Cme, *C. merolae*; Ect, *E. siliculosus*; Hpa, *H. arabidopsidis*; Pfa, *Pl. falciparum*; Pin, *P. infestans*; Pul, *Py. ultimum*; Spr, *S. parasitica* (Spr); Tps, *T. pseudonana*; Vca, *V. carteri*.

Given that *A. laibachii* CHXCs show the closest clustering with *S. parasitica*, *V. carteri*, *Ch. reinhardtii*, and *Ar. thaliana* CHXCs (Figure 9), conceivably this candidate effector class evolved from an ancestral green-alga-derived gene.

Whatever their origin, we conclude that CHXC proteins are present in all organisms analysed but evolved effector function only in Albuginales and possibly Saprolegniales. In Albuginales, one N-terminal sub-class of CHXCs (CHxCLx(4)Gx(5–6)L) shows significant expansion, with 23 members, while other CHXCs are distinct from this clade. *S. parasitica* CHXCs are distinct from this major *A. laibachii* clade and therefore remain to be tested in future experiments.

### Conclusions

**Figure 10:**
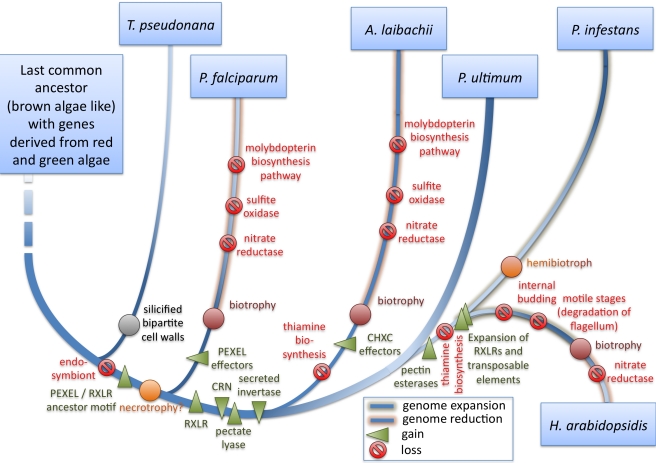
Gain and loss of genes and pathways for selected Chromalveolata in comparison to *A. laibachii*. It was hypothesized that the last common ancestor of Chromalveolata was a brown-alga-like organism with genes from green and red algae integrated into the nuclear genome after primary and secondary endosymbiosis [32],[37]. While some heterokonts kept their secondary endosymbiont and, in the case of diatoms, acquired a silicated bipartite cell wall [97], others lost their secondary endosymbiont. We postulate that after the loss of the endosymbiont, convergent evolution led to effector proteins like PEXEL [49],[98] and RXLR precursors. PEXEL effectors might have enabled *Pl. falciparum* to achieve more complex interactions with its host and establish intercellular growth. In addition to the RXLR effector proteins, oomycetes acquired or evolved another class of effectors, the CRNs [60] and a secreted invertase that allows use of sucrose from host plants [99]. Oomycetes that are biotrophs or hemibiotrophs today lost their thiamine biosynthesis pathway and, in the case of *A. laibachii*, evolved a new “CHXC” effector class. After taking up the biotroph lifestyle, the genomes of *Pl. falciparum*, *H. arabidopsidis*, and *A. laibachii* started a gene reduction that is exemplified by looking at enzymes that require molybdenum cofactors and the molybdopterin biosynthesis pathway. Hemibiotroph *P. infestans* instead shows a strong genome expansion [9]. In this context, *H. arabidopsidis* showed a genome expansion and acquired biotrophy late, based on the loss of only one molybdenum-dependent enzyme.

The *A. laibachii* genome assembly sheds light on the evolution of biotrophy since it allows the first comparison, to our knowledge, of two oomycete obligate biotroph pathogens (*A. laibachii* and *H. arabidopsidis*) that evolved biotrophy independently. In addition, *A. laibachii* shows the highest overall amino acid identity to the necrotroph pathogen *Py. ultimum* and the hemibiotroph *P. infestans*. One of the striking results of this comparison is that all organisms able to build haustoria have lost their thiamine biosynthesis pathway, presumably because thiamine is easily obtained from hosts via the haustorial interface. A closer interface requires effective host defence suppression. We therefore hypothesize that the evolution of biotrophy involves a series of steps: step 1, involving progressively more effective effectors to suppress defence, step 2, attenuated activation of defence by reduction in the inventory of cell wall hydrolyzing enzymes, resulting in, step 3, weak selection to maintain certain biosynthetic pathways if the products of the pathways can be directly obtained from the host. This results in progressively more comprehensive auxotrophy and culminates in irreversible biotrophy (Figure 10).

## Materials and Methods

### Field Isolate

An infected leaf was harvested from an *Ar. thaliana* plant grown in a heavy infected field plot in Norwich (UK; 52.6236,1.2182) [21] in December 2007. Zoosporangia were washed off the leaf surface and used to infect *Ar. thaliana* Ws-0-eds1 plants. After 1 wk one pustule was punched out, and spores were placed on ice for 30 min to release zoospores. Unhatched zoosporangia were removed by filtration, and zoospores were diluted to ∼10 zoospores/ml and sprayed on *Ar. thaliana* Ws-0 plants (∼100 µl/plant). This procedure was repeated 4× until spores were bulked up on *Ar. thaliana* Ws-0 plants. Zoosporangia were harvested using a home-made cyclone spore collector [71].

### Plant Inoculation

Zoospores were suspended in water (10^5^ spores/ml) and incubated on ice for 30 min. The spore suspension was then sprayed on plants using a spray gun (∼700 µl/plant), and plants were incubated in a cold room in the dark over night. Infected plants were kept under 10-h light and 14-h dark cycles with a 20°C day and 16°C night temperature.

### DNA Extraction and Sequencing

High molecular weight DNA was extracted from zoosporangia using a phenol/chloroform-based purification method after grinding in liquid nitrogen, adapted from [72]. Library preparation for Illumina sequencing was performed as described [28]. All data were generated using paired-end reads. 800 bp and 400 bp paired-end sequencing libraries were constructed, and 8.8 Gbp of usable data were generated (for read and insert length, see Figure 1A).

### Calculation of Expected Nc14 Genome Size

Figure 1A lists all reads after purification from plant and bacterial contamination as well as all reads aligned to the assembly. In summary, 91.6% of all reads can be aligned to the contigs, suggesting 2.8 Mbp missing from the assembly. Since 32.7 Mbp are in the assembly, the genome can be estimated to 35.5 Mbp. In another approach considering all reads and their read length, 8.8 Gbp (∼7% correction for lower quality of second read pair) were generated, which would lead to an expected coverage of the 32.7 Mbp genome of ∼270×. The mean coverage using single copy genes (glycolysis and TCA) is 240×. Considering the 2.5 Mbp of repeats (Figure 1B, right side, coverage underestimated) with an average coverage of 1,086×, which is ∼4.4 times more than the mean coverage of the contigs, this repeat region corresponds to 10.9 Mbp. In contrast to this, the genome contains ∼6.2 Mbp of hemizygous regions (Figure 1B, left side, coverage overestimated). These calculations suggest a genome size of ∼43 Mbp, given all repeats resolved, or an effective genome size of ∼37 Mbp.

### cDNA Preparation and Sequencing

*A. laibachii*–infected *Ar. thaliana* Ws-0 plants were harvested 0 (after cold room, see plant inoculation), 2, 4, 6, 8 and 10 d after infection. Total RNA was extracted using TRI Reagent RNA Isolation Reagent (Sigma), and Dynabeads (Invitrogen) were used to enrich for mRNA. First and second strand cDNA synthesis was performed according to manufacturer's instructions using the SMART cDNA Library Construction Kit (Clontech), and cDNA was normalized using the Trimmer kit from Evrogen. cDNA samples were mixed in equal amounts and fragmented using a Covaris sonicator (Covaris). Illumina libraries were prepared as described for fragmented genomic DNA [28].

### Data Acquisition

Data for comparative genomics were downloaded from the sources listed in Table 3.

**Table 3:** Sequence sources for comparative genomics analyses.

Organism	Download Site	Reference	Genome Version	Annotation/Proteins	Download Site Host
*Chlamydomonas reinhardtii*	http://genome.jgi-psf.org/Chlre4/Chlre4.home.html	[100]	4.0	3.1	DOE Joint Genome Institute
*Ectocarpus siliculosus*	http://www.ebi.ac.uk/ena/	[101]	2.0	20100616100000	European Bioinformatics Institute
*Phaeodactylum tricornutum*	http://genome.jgi-psf.org/Phatr2/Phatr2.download.ftp.html	[102]	2.0	20070523	DOE Joint Genome Institute
*Thalassiosira pseudonana*	http://genome.jgi-psf.org/Thaps3/Thaps3.info.html	[103]	3.0	2.0	DOE Joint Genome Institute
*Saccharomyces cerevisiae*	ftp://genome-ftp.stanford.edu/pub/yeast/sequence/genomic_sequence/	[104]	Nov 30 2006	Jan 06 2010	Saccharomyces Genome Database
*Debaryomyces hansenii*	http://www.ebi.ac.uk/2can/genomes/eukaryotes/Debaryomyces_hansenii.html	[105]	CR382133.2	CR382133	European Bioinformatics Institute
*Toxoplasma gondii*	http://toxodb.org/common/downloads/release-6.0/Tgondii/	[106]	6.0	6.0	ToxoDB
*Plasmodium falciparum*	http://plasmodb.org/common/downloads/release-6.3/Pfalciparum/	[107]	6.3	6.3	PlasmoDB
*Homo sapiens*	ftp://iubio.bio.indiana.edu/eugenes/2003/man/	[108]	Jun 23 2002	Jun 24 2002	IUBio Archive
*Takifugu rubripes*	http://www.fugu-sg.org/downloads/downloads3.htm	[109]	5	5	Institute of Molecular and Cell Biology
*Phytophthora sojae*	http://genome.jgi-psf.org/Physo1_1/Physo1_1.home.html	[110]	1.1	1.1	DOE Joint Genome Institute
*Phytophthora infestans*	http://www.broadinstitute.org/annotation/genome/phytophthora_infestans/MultiDownloads.html	[9]	4.1	4.1	Broad Institute
*Saprolegnia parasitica*	http://www.broadinstitute.org/annotation/genome/Saprolegnia_parasitica/Downloads.html	[111]	1	1	Broad Institute
*Pythium ultimum*	http://pythium.plantbiology.msu.edu/download.html	[10]	Release 1	Release 1	Michigan State University
*Hyaloperonospora arabidopsidis*	http://vmd.vbi.vt.edu/download/index.php	[4]	8.3.2	8.3.2	Virginia Bioinformatics Institute
*Ustilago maydis*	http://www.broadinstitute.org/annotation/genome/ustilago_maydis.2/MultiDownloads.html	[50]	Release 2	1	Broad Institute
*Fusarium oxysporum*	http://www.broadinstitute.org/annotation/genome/fusarium_group/MultiDownloads.html	[112]	2	2	Broad Institute
*Volvox carteri*	http://genome.jgi-psf.org/Volca1/Volca1.download.ftp.html	[113]	2	2	DOE Joint Genome Institute
*Cyanidioschyzon merolae*	http://merolae.biol.s.u-tokyo.ac.jp/download/	[114]	Jul 03 2007	Jan 18 2008	University of Tokyo

### Genome Assembly

First Velvet [24] was used, running different kmer-lengths and different sequencing library subsets (kmer-length: 23, 31, 41, 45, 49, 55, 61, 67, and 73; subsets: 400-bp insert only, 800-bp insert only). N50 number and length were determined for each of the assemblies, and the best assembly was selected as the matrix to be used with the Minimus2 genome merge pipeline [25]. For the current assembly the 400-bp only subset with kmer-length 61 was used as matrix, and for kmer-lengths 49, 55, 61, 67 and 73, all 400- and 800-bp assemblies were added (Minimus parameters: consensus error <0.001; minimum identity >99%; 20-bp maximum trimming). A set of genes showing high heterozygosity was used to ensure that contigs were properly joined. Parameters were changed through several rounds, and minimum overlap, in particular, was lowered from 100 bp to 15 bp. An overlap of 15 bp was found to be the optimum for difficult heterozygous regions. After each Minimus assembly, all reads were back aligned to the contigs using MAQ aligner [73]. Regions showing less than 3× average coverage were removed, and redundant fragments were removed using BLASTN with an e-value cut-off of 1e^−20^ and 99.9% identity. After this step a next round of Minimus was started, with changing minimum overlap in steps of 20 bp down from 100 bp. Below 20 bp steps were changed by 5 bp (See Figure S1 for work flow).

Since it is impossible to cultivate obligate biotrophs under sterile conditions, plant and bacterial contaminations were removed by using BLAST against genome sequences of the host plant *Ar. thaliana* (TAIR 9.0), fungal genomes (*Neurospora crassa*), oomycetes (*H. arabidopsidis*), and diverse bacterial genomes (*Xanthomonas* sp. and *Pseudomonas* sp.).

### Prediction of Heterozygous Loci

To identify heterozygous loci, Illumina reads were aligned using MAQ, and the SNP detection pipeline was used according to the manual, with default parameters and minimum coverage greater than 180× for the Nc14 alignment and greater than 20× for the Em1 alignment. From the MAQ SNP file, positions were selected where two bases are possible and maximum coverage was less than 350×.

### Repetitive Elements

Assembled repetitive elements were identified using the RepeatScout program (http://bix.ucsd.edu/repeatscout/) with a seed size of 14. The frequency of elements and their location in the assembly were estimated with RepeatMasker using a library of repetitive elements built up by RepeatScout. A sequence was considered to be repetitive if it occurred in the genome assembly on at least three different contigs. The resulting library was searched for the sequences homologous to the known transposon elements using TBLASTX (e-value cut-off of 1e^−5^) and a database of transposons, RepBase [74]. Consensus repeats that matched predicted Nc14 protein coding genes were filtered out. The remaining consensus repeats that do not match any sequences deposited in the NCBI database or any known transposon element and that do not overlap with Nc14 protein coding genes represent either *Albugo*-specific repeats or simple repeats.

tRNA genes were predicted with the program ARAGORN [75] using first default parameters and second options allowing introns in the gene sequences.

### Genome Quality Using CEGMA

CEGMA was used according to the manual [26] with a local installation.

### cDNA Assembly

For the combined ABySS [76] and Oases [77] assembly, adaptor sequences from the SMART kit cDNA synthesis were removed for the ABySS assembly, and the ABySS program was used according to the manual. Different kmer-lengths were tested, and a length of 61 used for the final assembly.

Untrimmed cDNA sequences were assembled using Velvet and a kmer-length of 51, 57, 61, and 71. Oases was used for the final assembly of the contigs according to the manual, using default parameters.

MUMmer in maxmatch mode was used to combine all ABySS and Velvet assemblies. Redundant contigs were removed using BLAST.

Since the assembled cDNA is not strand specific but orientation is needed for gene prediction, cDNA 5′ tags were generated by Illumina sequencing (E. Kemen, A. Balmuth, J. D. Jones, unpublished data). Using Bowtie aligner [78], cDNA 5′ tags were aligned onto the assembled cDNA and, based on tag counts, orientated in the 5′ to 3′ direction.

### cDNA Alignments

To map assembled cDNA against the genome, either BLAT [79] in trimT and fine mode or PASA [80] with default settings was used.

Illumina reads were directly mapped to the genome using the Bowtie aligner, in “best” mode and with strand correction (strandfix mode). Pileup files were generated using bowtie-maqconvert and maq pileup allowing four mismatches per 76-bp read. To incorporate this data as hints files for gene prediction, regions with greater than 3× coverage were extracted.

### Gene Prediction and Annotation

To generate a reliable gene set to train further programs, GeneMark [81] was used for *ab initio* gene prediction. ORFs plus 50 bp on the 3′ end and 50 bp on the 5′ end were extracted, and Illumina-sequenced cDNA was aligned to the ORFs using Bowtie. Gene models were selected if the coverage within the ORF didn't drop below three. This dataset with more than 2,000 genes was used as “traingenes” for the automated training program provided with the Augustus package (autoAug.pl). The trained Augustus program was then used for gene prediction including the combined Oases/ABySS-assembled cDNA (mapped using BLAT) as evidence. Default parameters (extrinsic.ME.cfg) were used for all predictions.

For consensus gene predictions with *P. infestans*, SGP2 was used according to the manual [82].

ASGARD [30] alignments were converted into GFF files to be used for consensus predictions.

Consensus gene models were generated using Evigan [83]. cDNA from assemblies and alignments was converted into GFF files and combined with Augustus, GeneMark, SGP2, and ASGARD predictions. The genome was than screened for gene-free regions, and Augustus gene predictions were added if available. In a third round, regions that did not contain consensus gene models or Augustus gene models were extracted, and GeneMark annotations were added if available.

A set of genes was further tested by 5′ and 3′ RACE to validate start and stop sites.

### Orthologous Genes and Divergence Level

Molecular divergence of *A. laibachii* from other species was assessed by examining the percentage of amino acid identity between orthologous gene pairs [75].

Orthologous pairs were identified using the OrthoMCL program with an e-value cut-off of 1e^−5^ [84]. Alignments of protein pairs were performed with MUSCLE [85].

Amino acid identity was calculated only for the single copy genes by either excluding alignment gaps from calculations or taking gaps into account. The results show similar trends, so we present only results for the calculations when alignment gaps were excluded.

The total number of orthologous groups identified between species and the number of one-to-one orthologous pairs, as well as a mean amino acid identity, are shown in Table S7. In the comparison of *T. gondii* and *A. laibachii*, we found few orthologous pairs represented by the single copy genes (23 pairs); therefore, we excluded this pair of species from the analyses of sequence divergence.

We also estimated the levels of amino acid identity for the core eukaryotic genes (orthologous genes shared by all examined species); these data are presented in Table S8.

### Green- and Red-Alga-Derived Genes

To identify *A. laibachii* genes with sequence similarity to green- or red-algal-derived diatom genes, a set published by Moustafa et al. [37] was used. All *A. laibachii* proteins showing homology to genes identified by Moustafa et al. [37] were further blasted (BLASTP) against the *Ch. reinhardtii* gene set, the *E. siliculosus* gene set, the *U. maydis* gene set, and the *Fusarium oxysporum* gene set with an e-value cut-off of 1e^−20^. Genes were considered to be green-alga-derived only if the protein was absent from *U. maydis* and *F. oxysporum* but present in *Ch. reinhardtii*, and was considered red-alga-derived if not in *U. maydis* or *F. oxysporum* but in *E. siliculosus*. The same analyses were performed on the *Saccharomyces cerevisiae*, *Pl. falciparum*, *H. arabidopsidis*, *P. infestans*, *Py. ultimum*, *V. carteri*, *Ch. reinhardtii*, *C. merolae*, *C. merolae*, *Th. pseudonana*, and *Ph. tricornutum* gene sets.

*A. laibachii* candidate genes with significant sequence similarity to green or red algae and other oomycetes (e-value cut-off of 1e^−20^) but not to fungi, brown algae, or diatoms were identified using the criteria in Table 4. Representative organisms for each group are as follows: green algae: *V. carteri*, *Ch. reinhardtii*; red algae: *C. merolae*, *Galdieria sulphuraria*; fungi: *F. oxysporum*; brown algae: *E. siliculosus*; diatoms: *Ph. tricornutum*, *Th. pseudonana*; oomycetes: *P. sojae*, *Py. ultimum*, *H. arabidopsidis*.

**Table 4:** Criteria for identification of red- and green-alga-derived genes.

Category	Presence/Absence					
	Green Algae	Red Algae	Fungi	Brown Algae	Diatoms	Oomycetes
Genes of Nc14 with significant sequence similarity with green algae	+	−	−	−	−	+
Genes of Nc14 with significant sequence similarity with red algae	−	+	−	−	−	+

Homologues between oomycetes, fungi, brown algae, and diatoms were identified using OrthoMCL (e-value cut-off of 1e^−20^ or 1e^−5^) [37].

### Synteny

Synteny between multiple species was analysed using the Artemis Comparison Tool [86]. Alignments between genomic sequences were performed using TBLASTX with a score cut-off of 210. Annotations of *P. infestans*, *Py. ultimum*, and *H. arabidopsidis* were transferred using TBLASTN with an e-value cut-off of 1e^−30^. LTR_FINDER [87] was used to annotate long terminal repeats (LTRs) within the genomic sequences, and coordinates were manually added. Regions between LTRs were blasted against RepBase [74] to identify the presence and/or type of transposon.

### Defining the Secretome

Secreted proteins were predicted using a local installation of SignalP 3.0 [88]. Proteins were considered to be secreted if both the neural networks and hidden Markov model methods predicted the protein to have a signal peptide. Predictions of TM domains were performed after removing the predicted secretion signal. TM domains were identified using MEMSAT3 [89]. Proteins were considered to be without a TM domain with *p*_non-TM_>0.0004 or, for high stringency, *p*_non-TM_>0.01.

### Motif Discovery

To identify new motifs, subsets of secreted proteins were selected and analysed using MEME [90] with default parameters. Identified motifs were tested against the whole gene set and the Swiss-Prot database using MOTIF Search. In a second step, motifs were selected only if they were positioned within 50 amino acids after the secretion signal.

Tests for over-representation of an identified motif were done using motif and sequence shuffling. Secreted proteins were predicted [88] as described in the previous section, and the signal peptide was removed prior to further analyses.

Each of the sequences without secretion signal was randomly shuffled 30 times. After each shuffling the sequences were screened for the motif in question. If the motif was identified after shuffling, the sequence was excluded from the next round. If the motif was never identified within the 30 times shuffling, the motif in the original protein was counted as “unique empirical”. All possible combinations of the amino acid sequence within the motif were calculated. For each of these permutations, the “unique empirical” proteins were calculated.

The 30 times shuffling was repeated 1,000 times to calculate background levels. Background levels were defined as how often a sequence was found again having the motif or the permutated motif. This was called “background (mean)”. Motifs that were above this background were considered for further analyses.

The second criterion was if a motif was significantly enriched in the secretome compared to all non-secreted proteins. For statistical validations we calculated the cumulative hypergeometric probability.

### Selection of Candidates for Further Experiments

Candidates for further experiments were evaluated according to a ranking list. Maximum possible score was nine points, and the following scores were given: one point for being on a shorter, repetitive contig (≤3,000 bp) or end of contig, since we assumed that effector candidates might be in repetitive regions as shown for *P. infestans* effectors [9]; one point for having cDNA support; two points for being a short protein (≤400 amino acids); two points for carrying one of the identified motifs (RXLR, RXLQ, CHXC, CRN); one point for being expressed before day 10 after infection; one point for being expressed before day 4 after infection; and one point for showing SNPs in the Em1 comparison.

### *P. capsici* Tests

#### Plant and bacterial growth procedures and *P. capsici* culturing

*N. benthamiana* plant genotypes and *P. capsici* strain LT1534 were grown and cultured as described by Schornack et al. [68]. *P. capsici* transformation was performed as described by Schornack et al. [68].

#### Plasmid construction and preparation

*Phytophthora* transformation constructs SP_AVR3aC, RXLR1_AVR3aC, CRN3_AVR3aC, CHXC9_AVR3aC, CHXC9AAAA_AVR3aC, CHXC7_AVR3aC, and CHXC7AAAA_AVR3aC were synthesized and cloned into pTOR by Genscript. Fusion genes were flanked by ClaI (5′) and SacII (3′), and internal AscI sites were inserted between the N-terminal effector domain and AVR3aC coding domain. N-terminal domains used are listed in Table 5.

**Table 5:** Summary of constructs generated for the *Phytophthora* infection assays.

Construct	N-Terminal Effector Domain (aa)	C-Terminal Avr3ac Domain (aa)
SP_Avr3aC	*P. infestans* Avr3a	*P. infestans* Avr3a KI_67–147_
RXLR1_Avr3aC	*A. laibachii* NC14 RXLR1_1–52_	*P. infestans* Avr3a KI_67–147_
CRN3_Avr3aC	*A. laibachii* NC14 CRN3_1–90_	*P. infestans* Avr3a KI_67–147_
CHXC9_Avr3aC	*A. laibachii* NC14 CHXC9_1–112_	*P. infestans* Avr3a KI_67–147_
CHXC9_AAAA_Avr3aC	*A. laibachii* NC14 CHXC9_1–112_, AAAA_41–44_	*P. infestans* Avr3a KI_67–147_
CHXC7_Avr3aC	*A. laibachii* NC14 CHXC7_1–107_	*P. infestans* Avr3a KI_67–147_
CHXC7_AAAA_Avr3aC	*A. laibachii* NC14 CHXC7_1–107_, AAAA_53–57_	*P. infestans* Avr3a KI_67–147_

#### *Phytophthora* infection assays

*Phytophthora* infection assays were performed according to Schornack et al. [68] with slight modifications.

#### Plasmid constructs

Vector pTOR::Avr3a and pTOR::Avr3a (AAAA-AAA) were obtained from Dr. Steve Whisson [91]. The control construct SP_Avr3aP was synthesized using the signal peptide of *P. infestans* Avr3a, fusing the signal peptide directly to the Avr3a C-terminus (GenBank accession number ACX46530.1). Translocation fusion constructs were synthesized using N-terminal coding sequences of *Albugo* RXLR1 (Gene name: AlNc14C278G10072, GI: 325190660), CRN3 (Gene name: AlNc14C196G8578, GI: 325188975), CHXC9 (Gene name: AlNc14C832G12555, GI: 325193652), and CHXC7 (Gene name: AlNc14C191G8449, GI: 325188831), and the AVR3a C-terminus (GenBank accession number ACX46530.1, GI: 260594559). CHXC9_AAAA_AVR3aC and CHXC7_AAAA_AVR3aC fusion constructs were synthesized by replacing the CHXC motif with a quadA motif. All plasmid suspensions used for *P. capsici* transformation were prepared using the Qiagen Midi Prep kit (Qiagen). For a summary of constructs see Table 5.

### Effector Detector Vector Assays

Candidate RXLR effectors were cloned from RXLR to stop; all other candidate effectors were cloned from SP cleavage site to stop into pENTR D-TOPO (Invitrogen) and mobilized into pEDV6 [70]. The resulting effector∶pEDV6 constructs were conjugated into Pst DC3000 luxCDABE [69] and Pst DC3000 ΔAvrPto/ΔAvrPtoB [92]. The contribution of an individual effector was assessed by spray inoculating 4- to 5-wk-old short day grown plants as previously described [93].

Growth of Pst DC3000 luxCDABE effector∶pEDV6 was calculated by measuring whole plant luminescence using a Photek camera system and normalizing this to plant fresh weight [69].

To assess the virulence of Pst DC3000 ΔAvrPto/ΔAvrPtoB effector∶pEDV6, bacterial colony counts were performed as previously described [94].

### Accession Numbers

All Illumina sequence reads generated during this study have been submitted to the Sequence Read Archive at EBI and are accessible under the accession number ERA015557. Individual studies are available with accession numbers ERP000440 (Alias: albugo_laibachii_nc14_dna_sequencing, http://www.ebi.ac.uk/ena/data/view/ERP000440), ERP000441 (Alias: albugo_laibachii_nc14_cdna_sequencing, http://www.ebi.ac.uk/ena/data/view/ERP000441), and ERP000442 (Alias: albugo_laibachii_em1_dna_resequencing, http://www.ebi.ac.uk/ena/data/view/ERP000442).

All contigs and annotations are available through EBI or NCBI. The accession range is from FR824046 to FR827861 (3,816 contigs including annotations) and can be accessed through the ENA browser (http://www.ebi.ac.uk/ena/).

## Supporting Information

Figure S1 **Assembly pipeline using Velvet and Minimus.** Blue boxes with white filling indicate the different Velvet assemblies used. For the Minimus assembler the best contig was used as a seed leading to supercontigs v1. Mis-assemblies in this version were identified and corrected by back aligning all reads (Figure 1A) using MAQ [73] and Bowtie [78]. A self-BLAST was used to avoid redundancy in the contigs. This pipeline was retrained using RACE data of highly heterozygous regions using contig-spanning genes.
(TIF)

Figure S2 **The continuity and quality of the assembled contigs were assessed using CEGMA.** In terms of core eukaryotic genes, 93.6% of a selected set of 248 genes could be detected. While 98.4% and 100%, respectively, of the highly conserved classes 3 and 4 were detected, 86.4% and 89.3%, respectively, of the more divergent classes 1 and 2 were found. Since CEGMA distinguishes between partial and full-length predicted genes, it allows studying the continuity of the genome as well. For the *A. laibachii* Nc14 genome only poorly conserved proteins show an elevated number in partial compared to full-length genes. For groups 2, 3, and 4, all genes predicted were present in full length, indicating that none of the genes was split over contigs. The Illumina-assembled Panda genome and the Sanger/Illumina combined genome of *H. arabidopsidis* were compared (dotted lines). The Panda genome shows high fragmentation of genes, indicated by the distance between partial and complete annotations. The *H. arabidopsidis* genome shows high continuity and a high detection level, although some genes are fragmented in the highly conserved class 4.
(TIF)

Figure S3 **Synteny between the** ***A. laibachii*** **Nc14 draft, the** ***P. infestans*** **Ia, and the** ***Py. ultimum*** **mitochondrial sequence.** The much bigger size of the *Py. ultimum* mitochondrial genome is due to a ∼22-kb inverted repeat [10]. Several regions within the *A. laibachii* mitochondrion show direct synteny (red) and inverted synteny (blue), reflecting regions within the *Py. ultimum* inverted repeats. The same region is not inverted in comparison to the *P. infestans* mitochondrion (far left and far right contigs of the *A. laibachii* assembly). Gene annotation in the *P. infestans* genome (annotated by BLAST from the protein sequences) shows that some genes don't show synteny in the *A. laibachii* Nc14 sequence, which is due to unresolved tRNA sequences. Genes in regions with synteny are in particular genes coding for ribosomal proteins, NADH dehydrogenase, and cytochrome C oxidase.
(TIF)

Figure S4 **Annotation of tRNA genes.** The trend shows that copy number correlates with possible codons and amino acid usage in the proteome. Exceptions are the tRNA for the start codon that encodes Met and for the codons that encode Val and Pro.
(TIF)

Figure S5 **Gene prediction pipeline and quality control.** (A) To ensure the best possible gene calls, we combined trained (Augustus), *ab initio* (GeneMark), and consensus (SGP2) gene predictions. Consensus gene calls were made using Evigan based on cDNA evidence. Evidence was generated either by direct alignment of cDNA reads from different stages of infection using Bowtie or by assembling the cDNA using Velvet in combination with Oases or/and using ABySS. (B) For validation of these gene models, a set of 860 annotated KOGs was compiled and tested. Results indicate that 75% of these groups are present in the current annotation. For comparison, 78% of KOGs were present in *P. infestans*, 73% in *H. arabidopsidis*, 42% in *Pl. falciparum*, and 85% in *Ar. thaliana*.
(TIF)

Figure S6 **Genes of “green” or “red” origin present in diatoms and a set of other chromalveolates.** Diagram showing the fraction of genes that are in common between the diatom *Ph. tricornutum* and the tested species that are integrated into the nuclear genome but are of green alga or red alga origin [37]. Bars show the percent of genes present in *Ph. tricornutum*; lines show absolute numbers. Coloured bar below the diagram indicates systematic groups (yellow: fungi; light blue: Apicomplexa; blue: Oomycota; green: green algae; red: red algae; brown: brown algae; lilac: diatoms). The diagram shows that oomycetes still carry about 20% of the green-alga-derived genes that diatoms do. The brown alga *E. siliculosus* carries ∼60% of the green alga genes the diatoms do. This might indicate that the ancestral brown algae contained far more green alga genes but these genes were replaced by red alga genes.
(TIF)

Figure S7 **Maximum likelihood trees inferred from comparisons of ITS2 (A) or MORN repeat proteins (B).** A comparison between both trees indicates incongruence between the ITS2 tree and the MORN repeat tree. The ITS2 tree reflects current systematics and indicates that brown algae and diatoms are closer to oomycetes than green algae are. Green algae build an isolated clade from brown algae and chromalveolates. The MORN repeat analyses indicate closer clustering of green algae to brown algae and oomycetes than to diatoms and apicomplexans. These analyses might support a hypothesis that brown-alga-like ancestors accumulated green alga genes. (All bootstrap counts were calculated from 100 replications. Both trees are midpoint rooted.)
(TIF)

Figure S8 **Synteny of a region in** ***A. laibachii*** **containing the flagellar inner arm dynein 1 heavy chain alpha (essential for flagellar function) to** ***Py. ultimum*** **and** ***H. arabidopsidis*** **.** *Py. ultimum* is able to form mobile zoospores while *H. arabidopsidis* isn't. Compared to *A. laibachii* the region is expanded in *Py. ultimum* and *H. arabidopsidis*, but while *Py. ultimum* maintains the flagellar dynein, *H. arabidopsidis* shows a region with synteny but an insertion with homology to transposable elements. LTR sites were annotated using LTR_Finder (labelled in red).
(TIF)

Figure S9 **Gbrowse view of two repetitive regions in the** ***A. laibachii*** **Nc14 and** ***A. laibachii*** **Em1 genome.** Both regions contain RXLR effector candidates. (A) A highly conserved repeat region with ∼10 repeats in Nc14 and ∼6 repeats in Em1. (B) A more diverged repeat region with >80 repeats within the Nc14 genome but deletion of the gene-containing region within the Em1 repeats.
(TIF)

Figure S10 **Representation analyses of permutated CHXC motifs in the proteome of selected chromalveolates, red and green algae, and** ***Ar. thaliana*** **.** The analyses reveal that only *A. laibachii* and *S. parasitica* contain a significant number of CHXC-motif-containing proteins in the secretome. Only for *A. laibachii* is there a significant enrichment of secreted CHXCs over non-secreted CHXCs. All organisms show a significant number of CXHC proteins, with a high proportion of secreted proteins. CXHC proteins are conserved between genomes and are predominantly enzymes like disulphide isomerase.
(TIF)

Table S1 **Host range of** ***A. laibachii*** **Nc14 and** ***A. laibachii*** **Em1 tested on 126** ***Ar. thaliana*** **ecotypes.** Twelve ecotypes could be identified that show resistance to only one of the *A. laibachii* isolates, indicating a difference in host range (red: *Ar. thaliana* ecotypes resistant to both *A. laibachii* isolates; orange: ecotypes resistant to one; green: ecotypes susceptible to both).
(DOC)

Table S2 **Genes missing from the CEGMA prediction.** Genes not detected by CEGMA in the *A. laibachii* Nc14 assembly were further analysed and compared to the *P. infestans* genome and *H. arabidopsidis* Emoy2 genome. In all, 12 out of 28 core eukaryotic genes not predicted in *A. laibachii* Nc14 were not predicted in the other two oomycete genomes as well (light grey shading). In addition, three were present in only one of the tested genomes. To rule out the possibility that genes were not predicted because of unusual gene models that cannot be predicted by CEGMA, a BLAST and manual curation was performed on all missing candidates. Eleven could not be identified in the genome as well, while some genes gave multiple results (e.g., ATB binding domains) and were therefore ignored (labelled with “?”). The blast cut-off value was 1e^−20^. (Asterisk indicates partial genes.)
(DOC)

Table S3 **Primer pairs used to validate genome continuity and accuracy.** Genomic regions were selected and PCR amplified. The first column gives the primer name and orientation, the second column, primer sequence, the third column, expected length of the PCR product, and the last column indicates if the region could be amplified or not.
(DOC)

Table S4 **Repetitive elements in the** ***A. laibachii*** **assembly.** After a search of the library generated with RepeatScout for sequences homologous to transposons, we identified 270 consensus elements showing significant similarity to known transposons. The most abundant in the genome were *mariner* (DNA transposon) and *copia* (LTR retrotransposon) elements. Consensus repeats that do not match any deposited in the NCBI database and do not overlap with Nc14 protein coding genes are either *Albugo*-specific repeats (light grey background) or simple repeats. We identified 191 such consensus sequences that compose about 1% of the assembly.
(DOC)

Table S5 **Distribution of repetitive elements relative to contig length.** Out of the total 3,816 contigs in the assembly, 2,211 contigs have regions with similarity to transposons or other repetitive sequences. Most of these contigs (1,528 contigs) are less than 5,000 bp long.
(DOC)

Table S6 **Distribution of repeats matching telomeric consensus sequences.** Forward and reverse telomeric consensus sequences were identified with RepeatScout. A total of 45 contigs have repeats matching telomeric consensus sequences; amongst these, 25 contigs have telomeric repeats located either at the beginning or at the end of a contig. In all, 5,925 bp of telomeric repeats was assembled.
(DOC)

Table S7 **Characterisation of tRNA genes in the assembled** ***A. laibachii*** **contigs.** Type of tRNA gene, number of genes (without and with introns), number of anticodons, type of anticodon, and frequency of usage as a number of stars; 15 tRNA genes were predicted with introns.
(DOC)

Table S8 **Annotations for the brassinosteroid biosynthesis pathway.** The first column gives the enzyme commission numbers (EC numbers) of possible genes. The second column indicates gene names in *Ar. thaliana*. Question marks indicate genes that are difficult to annotate for a certain function (genes that belong to the superfamily of cytochrome P450s). The third column indicates genes identified using the ASGARD annotation pipeline, and the fourth column indicates manual annotation. (GI numbers in brackets.)
(DOC)

Table S9 **Potentially green-alga-derived genes that were identified based on results of a set of green- and red-alga-derived genes present in the diatom** ***Ph. tricornutum*** **.** Genes listed here had to be present in the green alga *Ch. reinhardtii* (chloroplast or nuclear genome) but had to be absent from the red alga *C. merolae* and from the fungi *F. oxysporum* and *U. maydis*. (Orange: in *A. laibachii*, *P. infestans*, *Py. ultimum*, *Ph. tricornutum*, *Th. pseudonana*, *Ch. reinhardtii*, and *Pl. falciparum* but not in *H. arabidopsidis* and *E. siliculosus*. Brown: as before but in *E. siliculosus*. Green: shared at least between *Pl. falciparum* and oomycetes. Annotations for identified genes were taken from the list published by Moustafa et al. [37].)
(DOC)

Table S10 **Green alga genes showing homology to** ***A. laibachii*** **genes but not to diatome, red alga, brown alga, or fungal genes.** Genes listed here had to be present in the green algae *Ch. reinhardtii* (chloroplast or nuclear genome) and *V. carteri* but had to be absent from the red alga *C. merolae*, the fungi *F. oxysporum* and *U. maydis*, and the brown alga *E. siliculosus* (for the BLAST analyses, an e-value cut-off of 1e^−20^ was used; proteins retained by repeating the analyses using an e-value cut-off of 1e^−5^ are indicated in blue).
(DOC)

Table S11 **Red alga genes showing homology to** ***A. laibachii*** **genes but not to diatom, green algae, brown alga, or fungal genes.** Genes listed here had to be present in the red algae *C. merolae* and *G. sulphuraria* but had to be absent from the green algae *Ch. reinhardtii* (chloroplast or nuclear genome) and *V. carteri*, the fungi *F. oxysporum* and *U. maydis*, and the brown alga *E. siliculosus* (for the BLAST analyses, an e-value cut-off of 1e^−20^ was used; proteins retained by repeating the analyses using an e-value cut-off of 1e^−5^ are indicated in blue).
(DOC)

Table S12 **Potentially green-alga-derived genes that are present in the diatoms** ***Ph. tricornutum*** **and** ***Th. pseudonana*** **but not in** ***A. laibachii*** **Nc14.** Genes listed here had to be present in the green alga *Ch. reinhardtii* (chloroplast or nuclear genome) but had to be absent from *A. laibachii* Nc14, the red alga *C. merolae*, and the fungi *F. oxysporum* and *U. maydis*. Columns 3–7 show presence/absence in *Py. ultimum*, *P. infestans*, *H. arabidopsidis*, *Pl. falciparum*, and *E. siliculosus* using the same criteria. (a, absent; p, present. Annotations for identified genes were taken from the list published by Moustafa et al. [33].)
(DOC)

Table S13 **Molecular divergence of** ***A. laibachii*** **based on all orthologous genes.**
(DOC)

Table S14 **Molecular divergence of** ***A. laibachii*** **based on core eukaryotic gene pairs.**
(DOC)

Table S15 **Presence and absence of important metabolic enzymes.** Red indicates absence and green indicates presence of genes. Genes present were annotated or validated in each organism. Remarkable is the absence of all molybdopterin biosynthesis genes, and enzymes using the cofactor, in *A. laibachii* and *Pl. falciparum*. *P. infestans* and *H. arabidopsidis* each lack one of the molybdopterin biosynthesis enzymes but contain molybdopterin-dependent enzymes, which might indicate that other enzymes can compensate for the missing step; in case of B73, the missing enzyme might be replaced by a multifunctional Cnx1 or by high concentrations of Mo inside the cell [95].
(DOC)

Table S16 **List of all annotated proteins of** ***A. laibachii*** **that might be associated with pathogenicity.** Annotation and identification were done using Pfam and BLASTP against the NCBI database. Localisation was predicted using a local installation of WoLF PSORT [96]. SignalP 3.0 was used for secretion prediction.
(DOC)

Table S17 **Intraspecies comparison between Nc14 and Em1.** All genes, genes with a predicted secretion signal peptide and without a TM domain, genes representing KOGs, or genes carrying a CHXC, RXLR, or RXLQ motif were compared. The second column in the table indicates heterozygosity (het) within Nc14; the third column indicates heterozygous positions within Em1 (green) or homozygous (hom) SNPs between Nc14 and Em1 (blue). The fourth column shows Em1-specific heterozygous positions or SNPs corrected against Nc14 heterozygous positions carrying the same nucleotide in one of the haplotypes. Frequencies of non-synonymous and synonymous mutations (darker coloured fields, mutations per 100 bp) are almost balanced in the all-gene and KOG gene comparisons, while a comparison of all secreted proteins indicates a 3∶1 ratio (non-synonymous∶synonymous). RXLRs and, particularly, RXLQs show an imbalance (∼2∶1), with high variation due to the small sample size. CHXCs, with a ratio of ∼5∶1, show a significant imbalance in the comparison between Nc14 and Em1. Considering total number and percentage of genes with a ratio of non-synonymous/synonymous <1 or >1 (light-coloured fields), only KOG genes show a significantly higher number of genes with a value <1, while all other classes show more genes with a value >1.
(DOC)

Table S18 **CHXC and CXHC candidate genes.** This table gives an overview of all predicted CHXC (white background) and CXHC (grey background) candidates from various species. The first column of the table indicates name of CHXC or CXHC candidates used for the phylogenetic analyses (Figure 9). The second column indicates the species name, and the third column indicates the locus tag within the corresponding genome. The fourth column shows the best BLAST hit against the NCBI nr database with an e-value<10^−50^. The last column indicates the accession number of the best hit.
(DOC)

## Abbreviations

KOG: core eukaryotic orthologous group
LTR: long terminal repeat
Nc14: Norwich 14
Pst: *Pseudomonas syringae* pv. tomato
TM: transmembrane
