# Supplementary figures and images for: Gene Gain and Loss during Evolution of Obligate Parasitism in the White Rust Pathogen of Arabidopsis thaliana
Source: PLoS Biol. 2011 Jul 5;9(7):e1001094. doi: 10.1371/journal.pbio.1001094 (PMC3130010; doi:10.1371/journal.pbio.1001094)

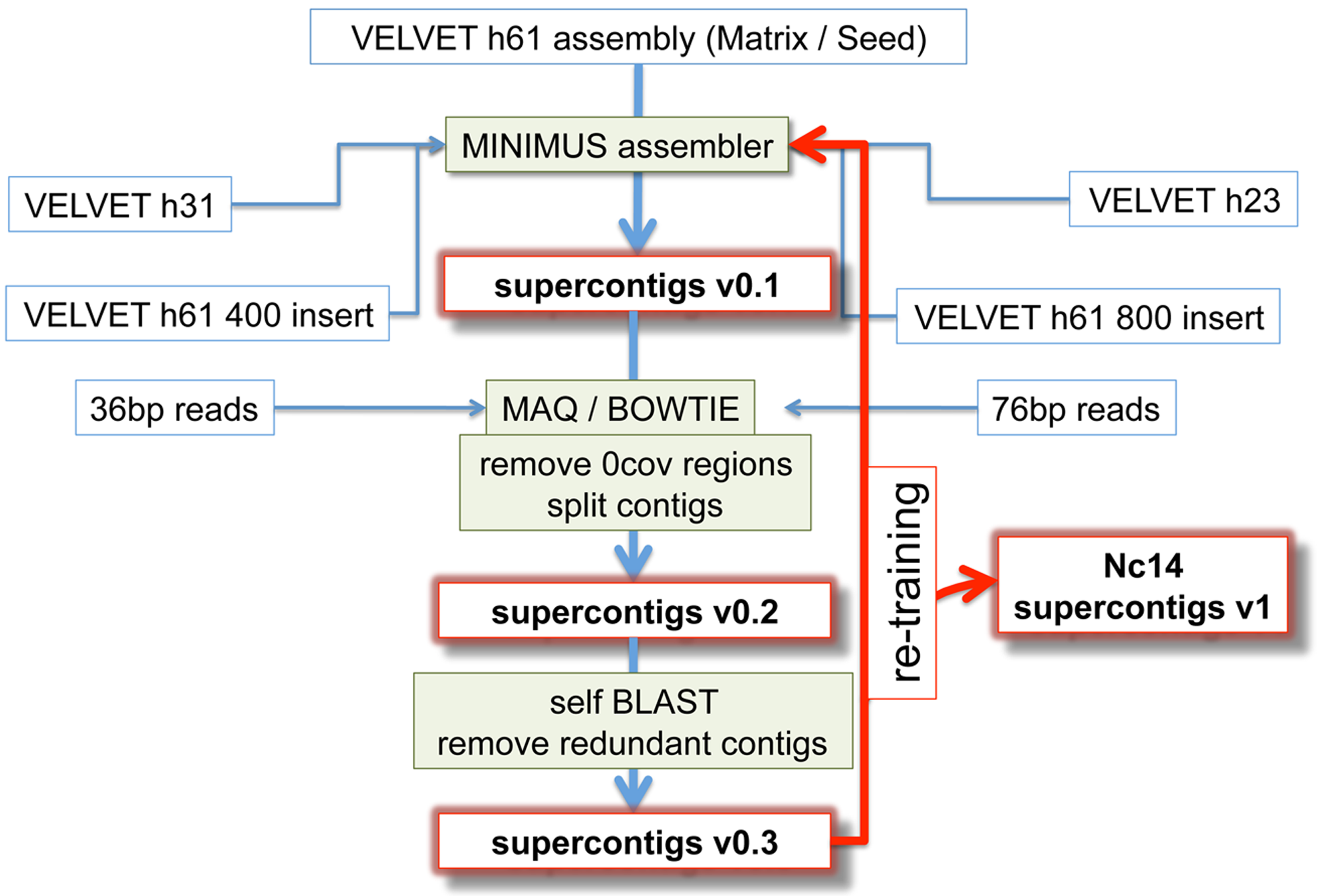

Supplement: Figure S1 — Assembly pipeline using Velvet and Minimus. Blue boxes with white filling indicate the different Velvet assemblies used. For the Minimus assembler the best contig was used as a seed leading to supercontigs v1. Mis-assemblies in this version were identified and corrected by back aligning all reads (Figure 1A) using MAQ [73] and Bowtie [78]. A self-BLAST was used to avoid redundancy in the contigs. This pipeline was retrained using RACE data of highly heterozygous regions using contig-spanning genes. (TIF) [file pbio.1001094.s001.tif]

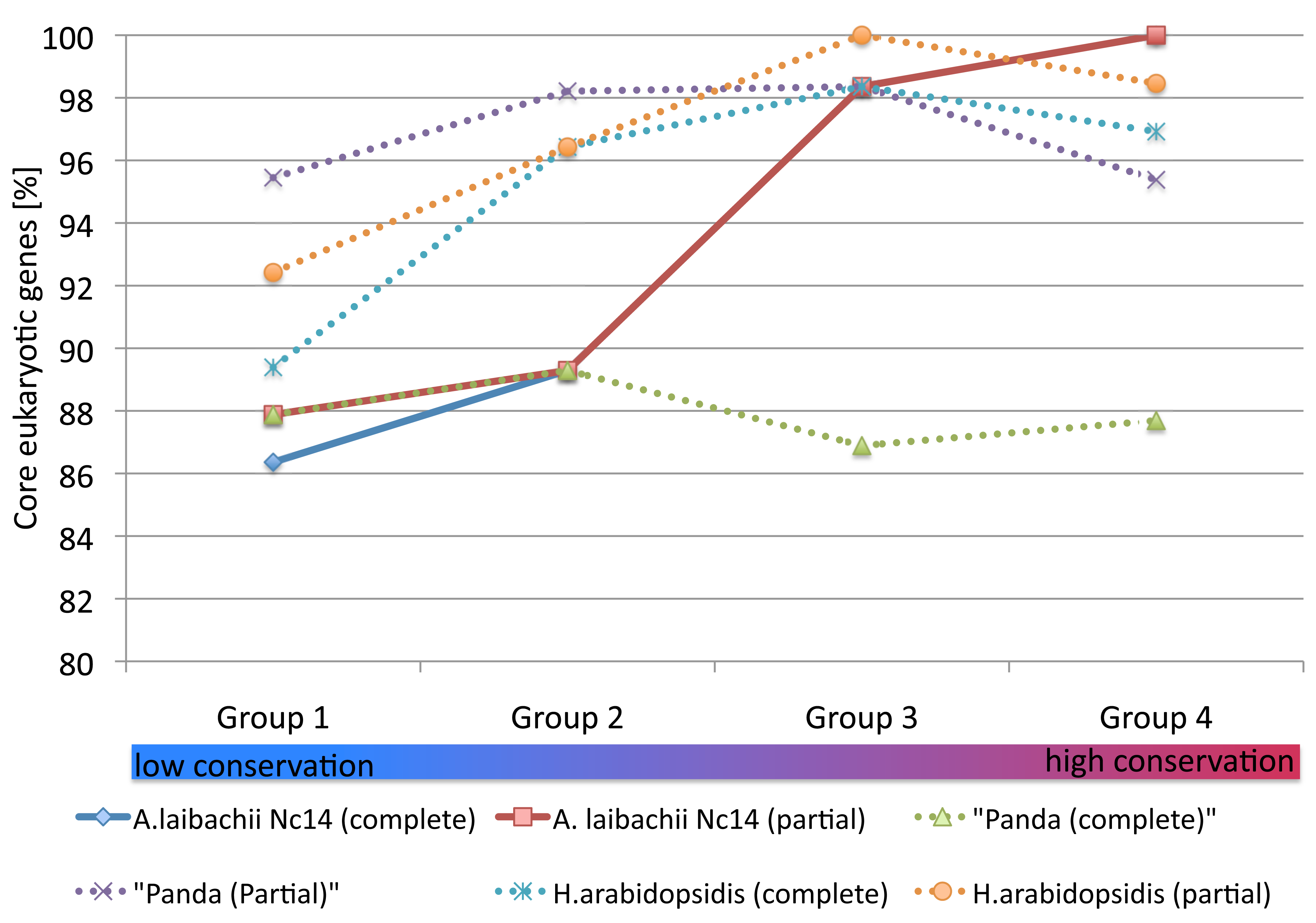

Supplement: Figure S2 — The continuity and quality of the assembled contigs were assessed using CEGMA. In terms of core eukaryotic genes, 93.6% of a selected set of 248 genes could be detected. While 98.4% and 100%, respectively, of the highly conserved classes 3 and 4 were detected, 86.4% and 89.3%, respectively, of the more divergent classes 1 and 2 were found. Since CEGMA distinguishes between partial and full-length predicted genes, it allows studying the continuity of the genome as well. For the A. laibachii Nc14 genome only poorly conserved proteins show an elevated number in partial compared to full-length genes. For groups 2, 3, and 4, all genes predicted were present in full length, indicating that none of the genes was split over contigs. The Illumina-assembled Panda genome and the Sanger/Illumina combined genome of H. arabidopsidis were compared (dotted lines). The Panda genome shows high fragmentation of genes, indicated by the distance between partial and complete annotations. The H. arabidopsidis genome shows high continuity and a high detection level, although some genes are fragmented in the highly conserved class 4. (TIF) [file pbio.1001094.s002.tif]

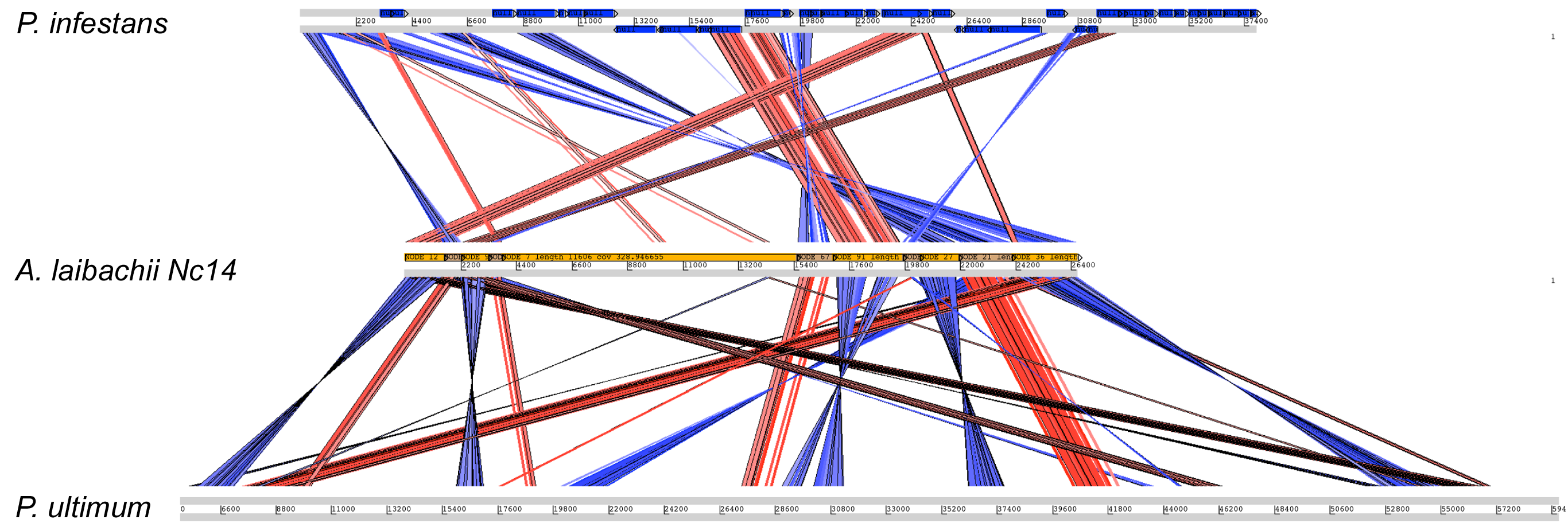

Supplement: Figure S3 — Synteny between the A. laibachii Nc14 draft, the P. infestans Ia, and the Py. ultimum mitochondrial sequence. The much bigger size of the Py. ultimum mitochondrial genome is due to a ∼22-kb inverted repeat [10]. Several regions within the A. laibachii mitochondrion show direct synteny (red) and inverted synteny (blue), reflecting regions within the Py. ultimum inverted repeats. The same region is not inverted in comparison to the P. infestans mitochondrion (far left and far right contigs of the A. laibachii assembly). Gene annotation in the P. infestans genome (annotated by BLAST from the protein sequences) shows that some genes don't show synteny in the A. laibachii Nc14 sequence, which is due to unresolved tRNA sequences. Genes in regions with synteny are in particular genes coding for ribosomal proteins, NADH dehydrogenase, and cytochrome C oxidase. (TIF) [file pbio.1001094.s003.tif]

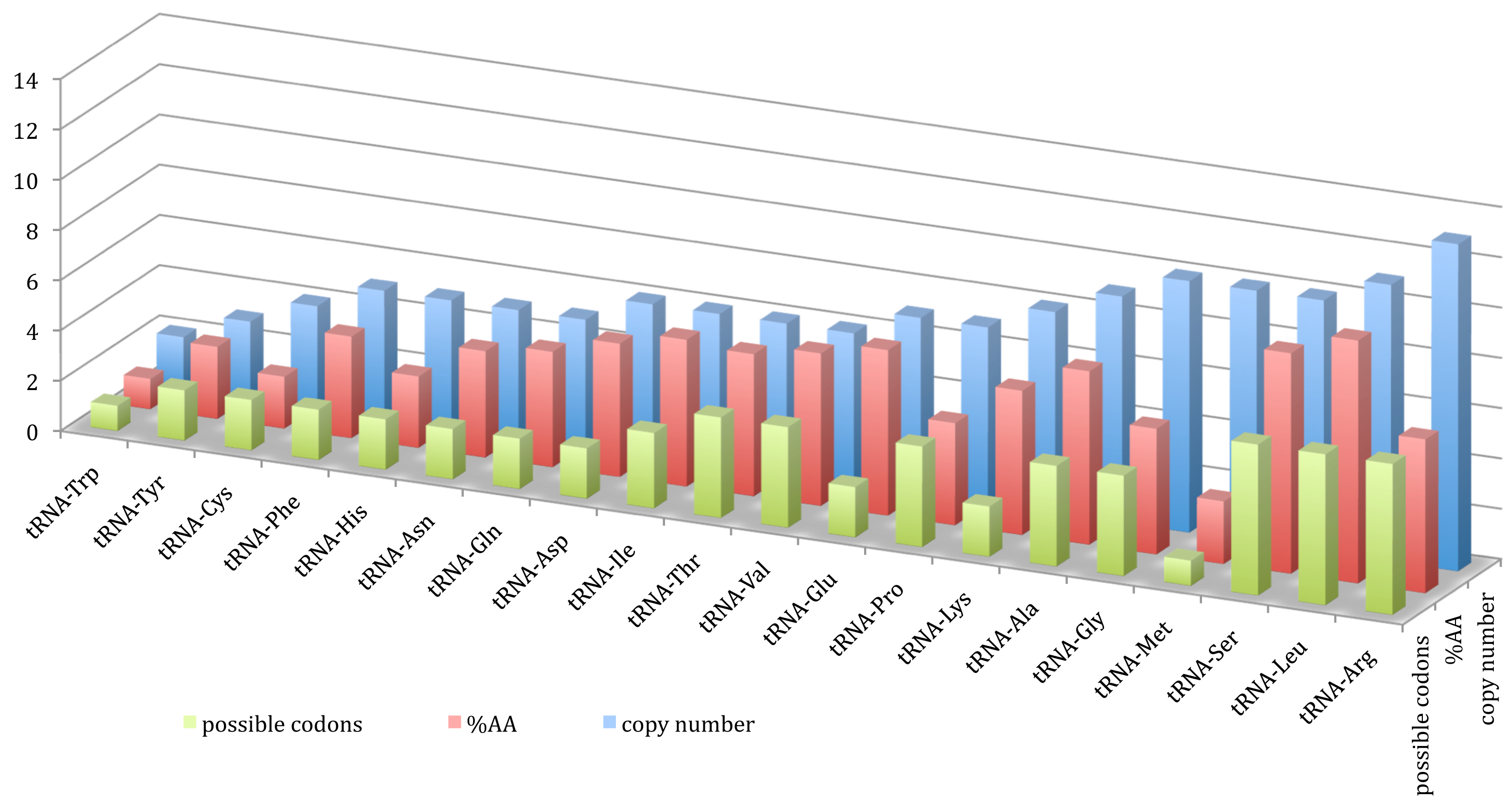

Supplement: Figure S4 — Annotation of tRNA genes. The trend shows that copy number correlates with possible codons and amino acid usage in the proteome. Exceptions are the tRNA for the start codon that encodes Met and for the codons that encode Val and Pro. (TIF) [file pbio.1001094.s004.tif]

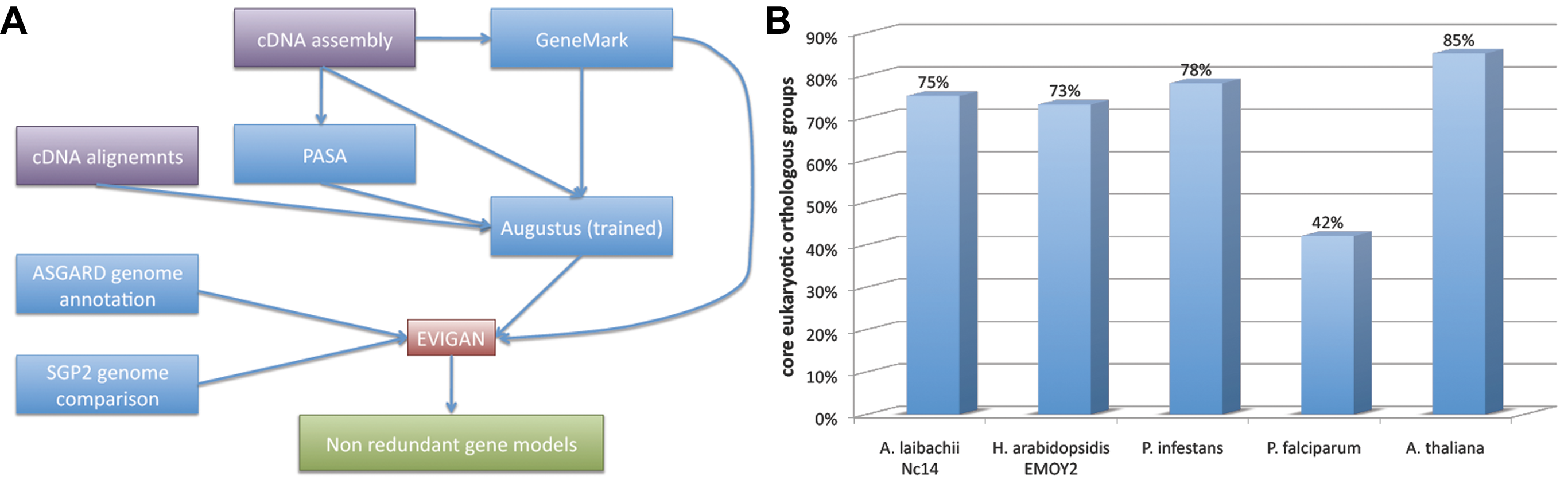

Supplement: Figure S5 — Gene prediction pipeline and quality control. (A) To ensure the best possible gene calls, we combined trained (Augustus), ab initio (GeneMark), and consensus (SGP2) gene predictions. Consensus gene calls were made using Evigan based on cDNA evidence. Evidence was generated either by direct alignment of cDNA reads from different stages of infection using Bowtie or by assembling the cDNA using Velvet in combination with Oases or/and using ABySS. (B) For validation of these gene models, a set of 860 annotated KOGs was compiled and tested. Results indicate that 75% of these groups are present in the current annotation. For comparison, 78% of KOGs were present in P. infestans, 73% in H. arabidopsidis, 42% in Pl. falciparum, and 85% in Ar. thaliana. (TIF) [file pbio.1001094.s005.tif]

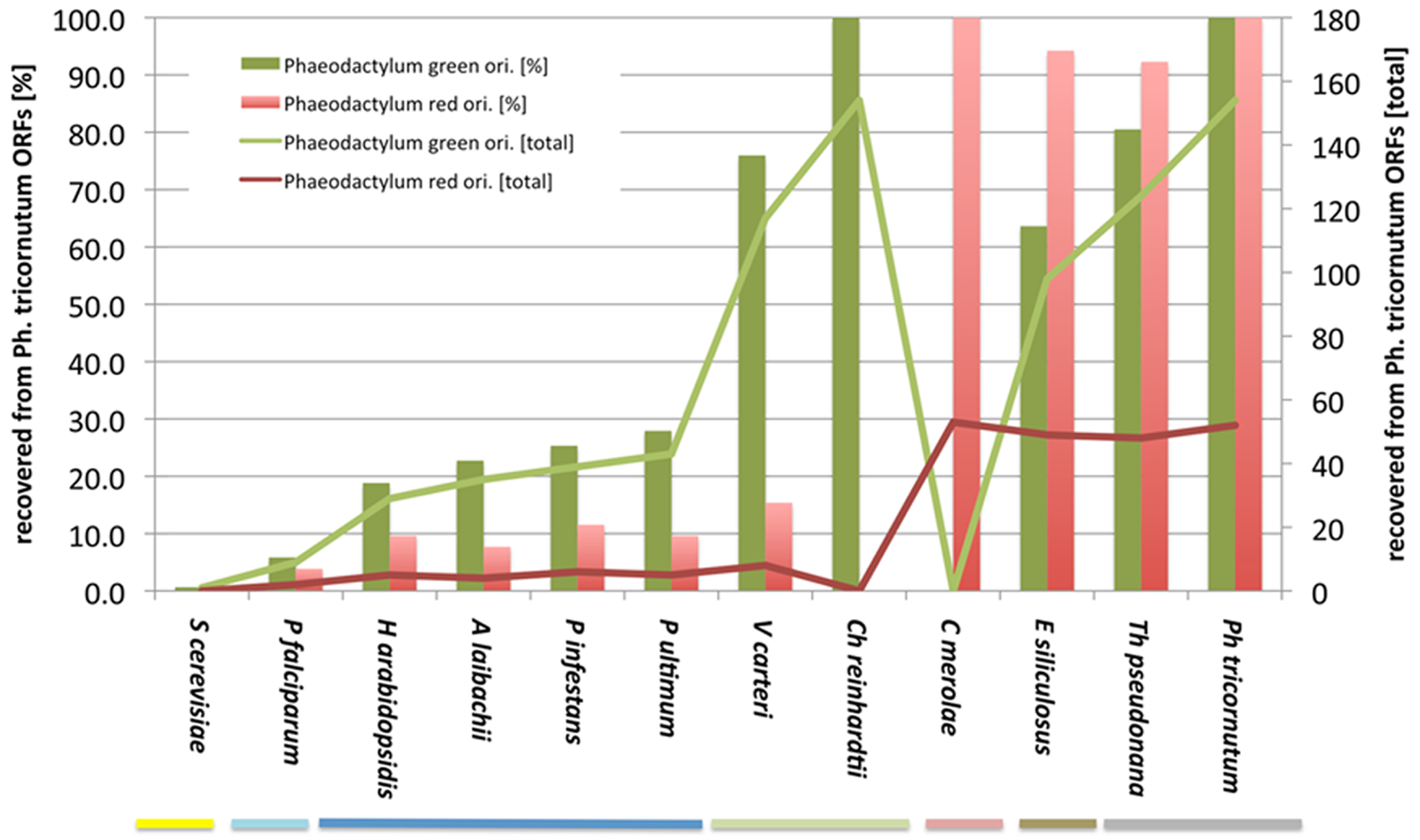

Supplement: Figure S6 — Genes of “green” or “red” origin present in diatoms and a set of other chromalveolates. Diagram showing the fraction of genes that are in common between the diatom Ph. tricornutum and the tested species that are integrated into the nuclear genome but are of green alga or red alga origin [37]. Bars show the percent of genes present in Ph. tricornutum; lines show absolute numbers. Coloured bar below the diagram indicates systematic groups (yellow: fungi; light blue: Apicomplexa; blue: Oomycota; green: green algae; red: red algae; brown: brown algae; lilac: diatoms). The diagram shows that oomycetes still carry about 20% of the green-alga-derived genes that diatoms do. The brown alga E. siliculosus carries ∼60% of the green alga genes the diatoms do. This might indicate that the ancestral brown algae contained far more green alga genes but these genes were replaced by red alga genes. (TIF) [file pbio.1001094.s006.tif]

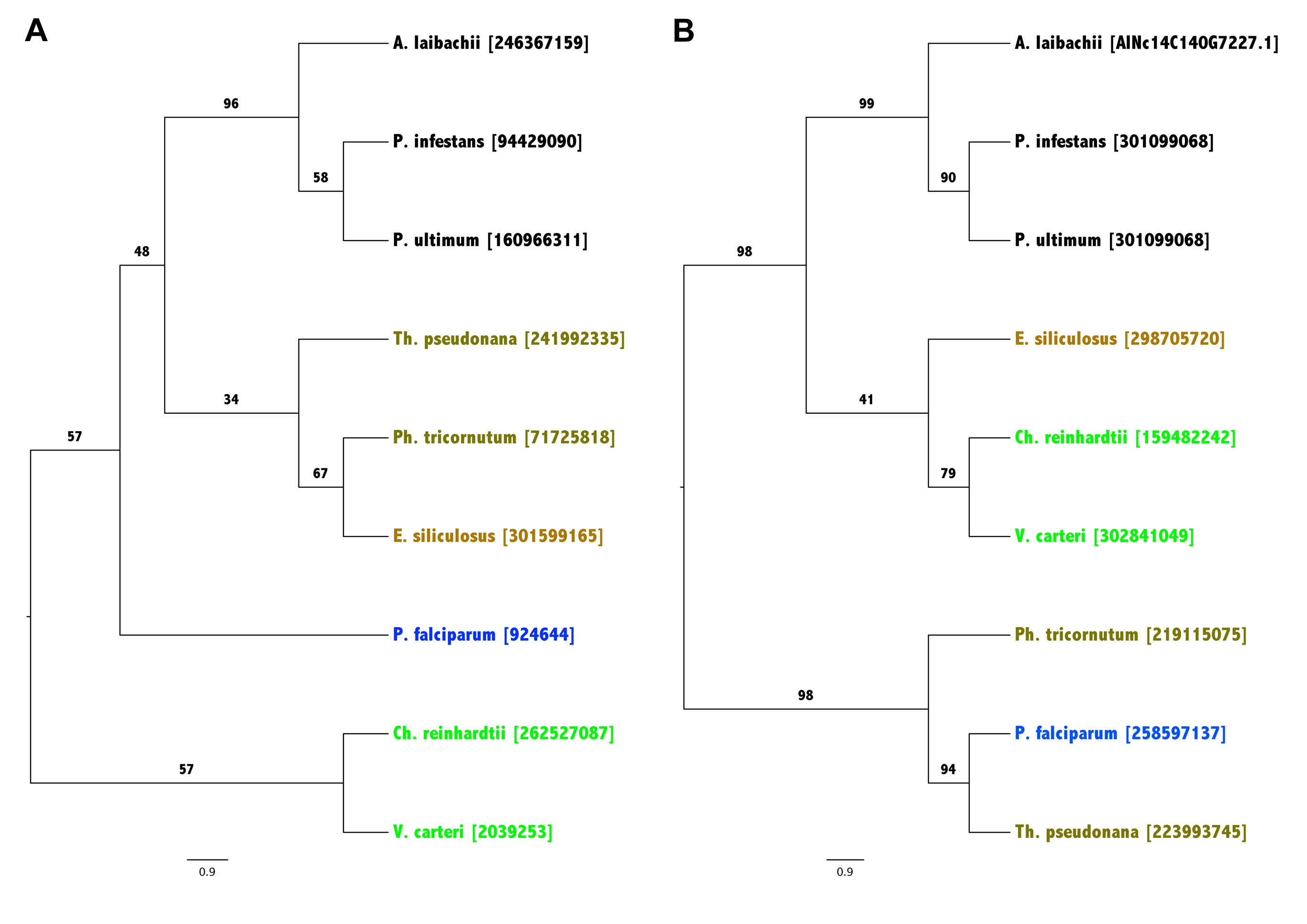

Supplement: Figure S7 — Maximum likelihood trees inferred from comparisons of ITS2 (A) or MORN repeat proteins (B). A comparison between both trees indicates incongruence between the ITS2 tree and the MORN repeat tree. The ITS2 tree reflects current systematics and indicates that brown algae and diatoms are closer to oomycetes than green algae are. Green algae build an isolated clade from brown algae and chromalveolates. The MORN repeat analyses indicate closer clustering of green algae to brown algae and oomycetes than to diatoms and apicomplexans. These analyses might support a hypothesis that brown-alga-like ancestors accumulated green alga genes. (All bootstrap counts were calculated from 100 replications. Both trees are midpoint rooted.) (TIF) [file pbio.1001094.s007.tif]

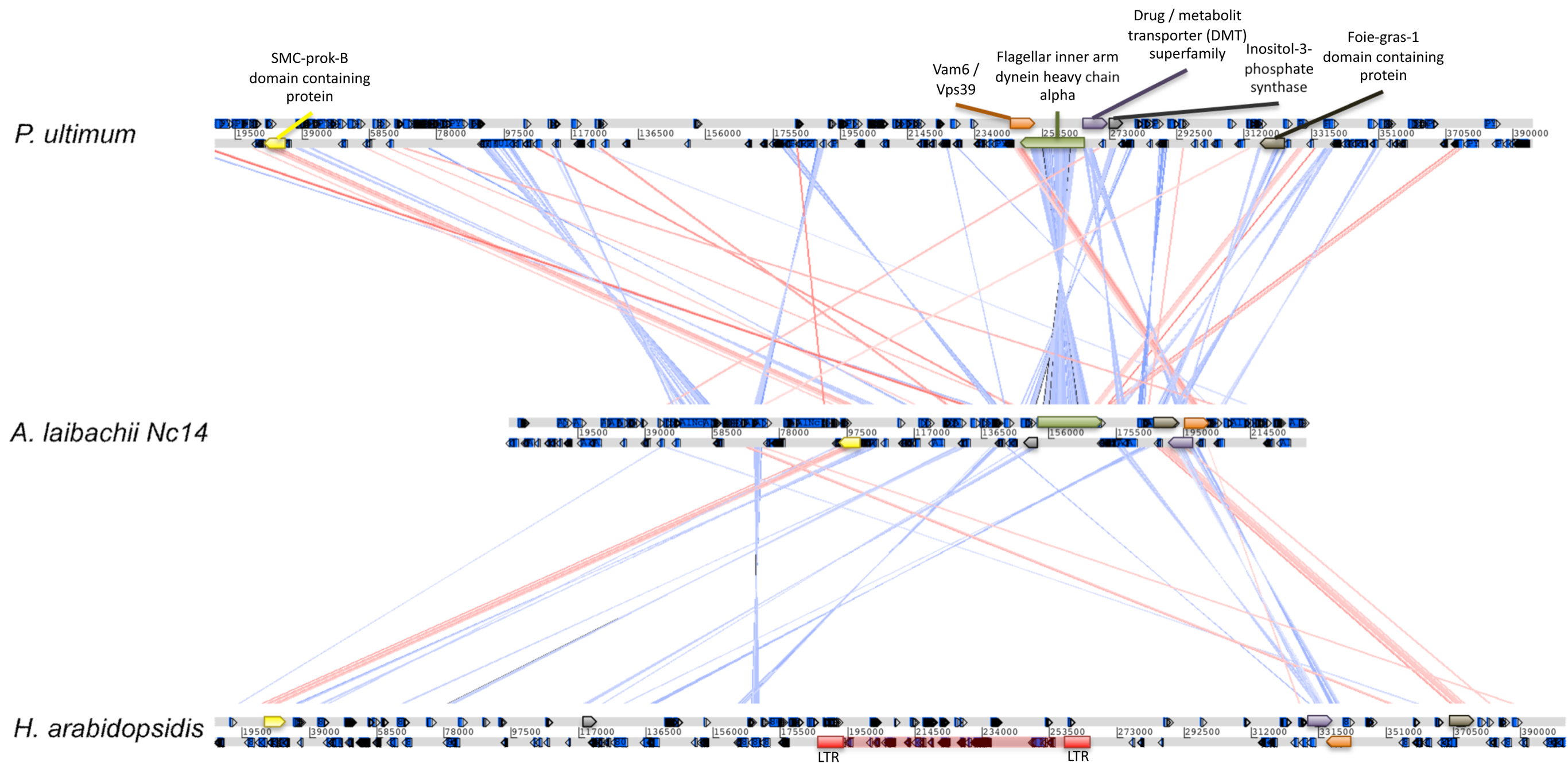

Supplement: Figure S8 — Synteny of a region in A. laibachii containing the flagellar inner arm dynein 1 heavy chain alpha (essential for flagellar function) to Py. ultimum and H. arabidopsidis . Py. ultimum is able to form mobile zoospores while H. arabidopsidis isn't. Compared to A. laibachii the region is expanded in Py. ultimum and H. arabidopsidis, but while Py. ultimum maintains the flagellar dynein, H. arabidopsidis shows a region with synteny but an insertion with homology to transposable elements. LTR sites were annotated using LTR_Finder (labelled in red). (TIF) [file pbio.1001094.s008.tif]

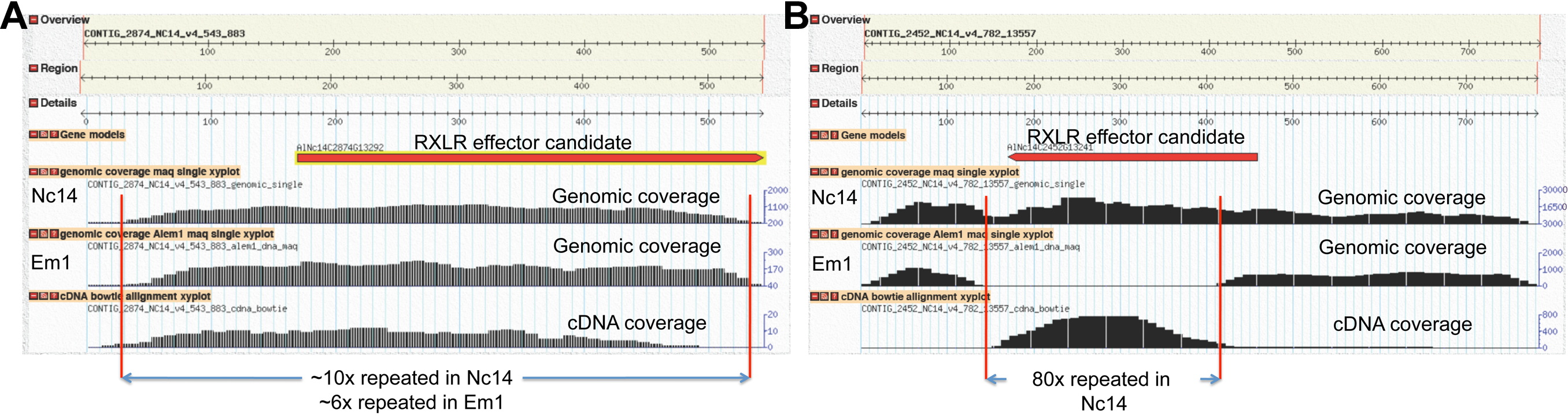

Supplement: Figure S9 — Gbrowse view of two repetitive regions in the A. laibachii Nc14 and A. laibachii Em1 genome. Both regions contain RXLR effector candidates. (A) A highly conserved repeat region with ∼10 repeats in Nc14 and ∼6 repeats in Em1. (B) A more diverged repeat region with >80 repeats within the Nc14 genome but deletion of the gene-containing region within the Em1 repeats. (TIF) [file pbio.1001094.s009.tif]

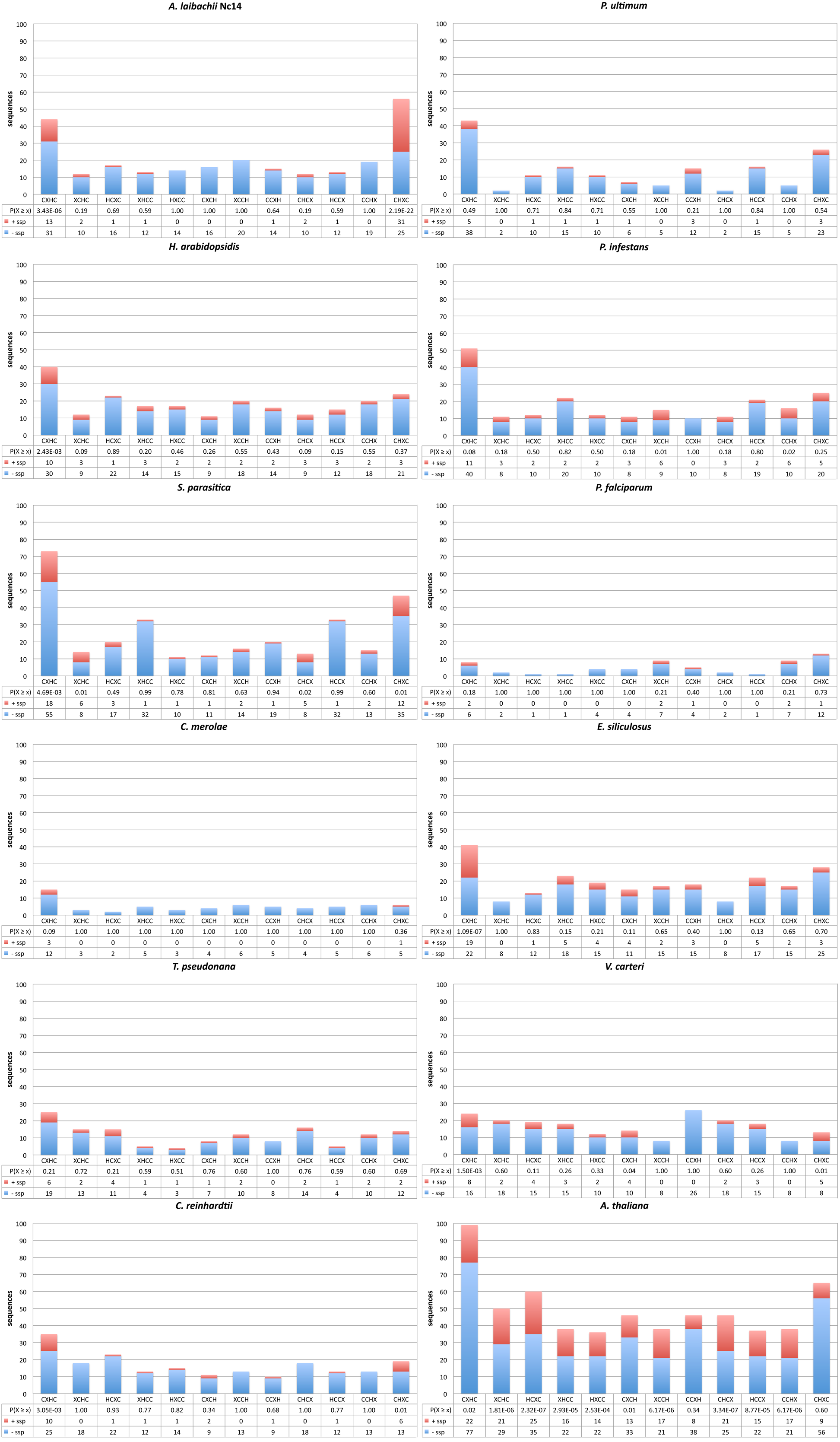

Supplement: Figure S10 — Representation analyses of permutated CHXC motifs in the proteome of selected chromalveolates, red and green algae, and Ar. thaliana . The analyses reveal that only A. laibachii and S. parasitica contain a significant number of CHXC-motif-containing proteins in the secretome. Only for A. laibachii is there a significant enrichment of secreted CHXCs over non-secreted CHXCs. All organisms show a significant number of CXHC proteins, with a high proportion of secreted proteins. CXHC proteins are conserved between genomes and are predominantly enzymes like disulphide isomerase. (TIF) [file pbio.1001094.s010.tif]
