# Supplementary material for: Gene Gain and Loss during Evolution of Obligate Parasitism in the White Rust Pathogen of Arabidopsis thaliana
Source: PLoS Biol. 2011 Jul 5;9(7):e1001094. doi: 10.1371/journal.pbio.1001094 (PMC3130010; doi:10.1371/journal.pbio.1001094)
Supplement: Table S1 — Host range of A. laibachii Nc14 and A. laibachii Em1 tested on 126 Ar. thaliana ecotypes. Twelve ecotypes could be identified that show resistance to only one of the A. laibachii isolates, indicating a difference in host range (red: Ar. thaliana ecotypes resistant to both A. laibachii isolates; orange: ecotypes resistant to one; green: ecotypes susceptible to both). (DOC) [file pbio.1001094.s011.doc]

| **Ecotype** | **Em1** | **Nc14** |
| --- | --- | --- |
| Ag-0 | S | S |
| An-1 | S | S |
| Bay-0 | S | S |
| Bil-5 | S | S |
| Bil-7 | S | S |
| Bor-1 | S | S |
| Bor-4 | S | S |
| Br-0 | S | S |
| Bur-0 | S | S |
| C24 | S | S |
| CIBC-17 | S | S |
| CIBC-5 | R | S |
| Col-0 | S | S |
| CS22491 | S | S |
| Ct-1 | S | S |
| Cvi-0 | S | S |
| Eden-1 | S | S |
| Eden-2 | S | S |
| Edi-0 | S | S |
| Ei-2 | R | S |
| Est-1 | S | S |
| Fab-2 | S | S |
| Fab-4 | S | S |
| Fei-0 | R | R |
| Ga-0 | S | S |
| Got-22 | S | S |
| Got-7 | S | S |
| Gu-0 | S | S |
| Gy-0 | S | S |
| HR-10 | R | R |
| HR-5 | S | R |
| Kas-1 | S | S |
| Kin-0 | R | S |
| Knox-10 | S | S |
| Knox-18 | R | R |
| Kondara | S | S |
| Kz-1 | S | S |
| Kz-9 | S | S |
| Ler-1 | S | S |
| LL-0 | S | S |
| Lov-1 | S | S |
| Lov-5 | S | S |
| Lp2-2 | S | S |
| Lp2-6 | S | S |
| Lz-0 | S | S |
| Mr-0 | S | S |
| Mrk-0 | S | S |
| Ms-0 | S | S |
| Mt-0 | S | S |
| Mz-0 | S | S |
| Nd-1 | S | S |
| NFA-10 | R | R |
| NFA-8 | S | S |
| Nok-3 | S | S |
| Omo2-1 | S | S |
| Omo2-3 | S | S |
| Oy-0 | S | S |
| Pna-10 | S | S |
| Pna-17 | R | S |
| Pro-0 | S | S |
| Pu2-23 | S | S |
| Pu2-7 | S | S |
| Ra-0 | S | S |
| Ren-1 | S | S |
| Ren-11 | S | R |
| Rmx-A02 | S | S |
| Rmx-A180 | S | S |
| RRS-10 | S | S |
| RRS-7 | R | S |
| Se-0 | S | R |
| Shahdara | S | S |
| Sorbo | S | S |
| Spr1-2 | S | S |
| Spr1-6 | S | S |
| Sq-1 | R | R |
| Sq-8 | S | S |
| Tamm-2 | S | S |
| Tamm-27 | S | S |
| Ts-1 | R | R |
| Ts-5 | R | S |
| Tsu-1 | S | S |
| Ull2-3 | S | S |
| Uod-1 | S | S |
| Uod-7 | S | S |
| Van-0 | S | S |
| Var2-1 | S | S |
| Var2-6 | S | S |
| Wa-1 | S | S |
| Wei-0 | S | S |
| Ws-0 | S | S |
| Ws-2 | S | S |
| Yo-0 | S | S |
| Zdr-1 | S | S |
| Zdr-6 | S | S |
| Sf2 | R | R |
| St-0 | S | S |
| Pa-3 | S | S |
| N6 (Karelian) | S | S |
| N7 (Pinguba) | S | S |
| N13 (Konchezero) | S | S |
| Eri-1 | S | S |
| Akita (252AV) | S | S |
| Bch-1 (37AV) | S | S |
| Blh-1 (180AV) | S | S |
| Co-4 (174AV) | R | R |
| Da(1)-12 (191AV) | S | S |
| Db-1 (132AV) | R | R |
| Enkheim-T (197AV) | S | S |
| Est-0 (71AV) | S | S |
| Ge-0 (101AV) | R | S |
| Gre-0 (200AV) | S | S |
| Hiroshima (254AV) | S | S |
| Ishikawa (253AV) | S | S |
| Ita-0 (157AV) | S | S |
| Jea (25AV) | S | S |
| Mh-0 (175AV) | S | S |
| Mh-0 (66AV) (b) | S | S |
| Nok-0 (492AV) | S | S |
| Nok-1 (95AV) | S | S |
| Pyl-1 (8AV) | R | S |
| Ri-0 (160AV) | S | S |
| Sakata (257AV) | S | S |
| Sp-o (53AV) | S | S |
| Tul-0 (240AV) | S | S |
| Uk-1 (113AV) | R | S |
| Uk-3 (521AV) | S | S |
