# Supplementary material for: Gene Gain and Loss during Evolution of Obligate Parasitism in the White Rust Pathogen of Arabidopsis thaliana
Source: PLoS Biol. 2011 Jul 5;9(7):e1001094. doi: 10.1371/journal.pbio.1001094 (PMC3130010; doi:10.1371/journal.pbio.1001094)
Supplement: Table S2 — Genes missing from the CEGMA prediction. Genes not detected by CEGMA in the A. laibachii Nc14 assembly were further analysed and compared to the P. infestans genome and H. arabidopsidis Emoy2 genome. In all, 12 out of 28 core eukaryotic genes not predicted in A. laibachii Nc14 were not predicted in the other two oomycete genomes as well (light grey shading). In addition, three were present in only one of the tested genomes. To rule out the possibility that genes were not predicted because of unusual gene models that cannot be predicted by CEGMA, a BLAST and manual curation was performed on all missing candidates. Eleven could not be identified in the genome as well, while some genes gave multiple results (e.g., ATB binding domains) and were therefore ignored (labelled with “?”). The blast cut-off value was 1e−20. (Asterisk indicates partial genes.) (DOC) [file pbio.1001094.s012.doc]

| KOG ID | Conservation group | Description | present in *P.infestans* | present in *H.arabidopsidis* | identified by blast gene id (gi number NCBI) | NCBI hit | e-vlaue |
| --- | --- | --- | --- | --- | --- | --- | --- |
| 0871 | 1 | Class 2 transcription repressor NC2, beta subunit (Dr1) | no | no | AlNc14C70G4842.1 (325184910) | XP_002901676.1 | 7E-63 |
| 1185 | 1 | Thiamine pyrophosphate-requiring enzyme | no | no | - |  |  |
| 1211 | 1 | Amidases | no | yes | AlNc14C12G1495.1 (325181146) | EFA83614.1 | 4E-74 |
| 1235 | 1 | Predicted unusual protein kinase | no | no | AlNc14C481G11893.1 ? (325192838) | XP_002874966.1 | 4E-129 |
| 1535 | 1 | Predicted fumarylacetoacetate hydralase | yes | yes | AlNc14C338G10753.1 (325191192) | XP_002894801.1 | 5E-101 |
| 1727 | 1 | Microtubule-binding protein (translationally controlled tumor protein) | no | no | - |  |  |
| 1760 | 1 | Molecular chaperone Prefoldin, subunit 4 | no | no | - |  |  |
| 2017 | 1 | Molybdopterin synthase sulfurylase | yes | yes | - |  |  |
| 2531 | 1 | Sugar (pentulose and hexulose) kinases | yes | yes | - |  |  |
| 2707 | 1 | Predicted metalloprotease with chaperone activity (RNAse H/HSP70 fold) | yes | yes | AlNc14C4G621.1 (325180149) | YP_001412625.1 | 4E-71 |
| 2785 | 1 | C2H2-type Zn-finger protein | yes | yes | AlNc14C35G3137.1 (325183012) | EFA84046.1 | 2E-42 |
| 2909 | 1 | Vacuolar H+-ATPase V1 sector, subunit C | no | no | AlNc14C622G12269.1 (325193322) | XP_002296350.1 | 4E-73 |
| 2967 | 1 | Uncharacterized conserved protein | yes | yes | ? |  |  |
| 3205 | 1 | Rho GDP-dissociation inhibitor | no | no | - |  |  |
| 3239 | 1 | Density-regulated protein related to translation initiation factor 1 (eIF-1/SUI1) | no | no | - |  |  |
| 3318 | 1 | Predicted membrane protein | yes | no | ? |  |  |
| 1394 | 2 | 3-oxoacyl-(acyl-carrier-protein) synthase (I and II) | no | yes | - |  |  |
| 1468 | 2 | Predicted translation initiation factor related to eIF-2B alpha/beta/delta subunits (CIG2/IDI2) | no | no | AlNc14C67G4702.1 (325184765) | XP_002997632.1 | 1E-105 |
| 1597 | 2 | Transcription initiation factor TFIIB | no | no | AlNc14C153G7570.1 (325187852) | XP_002898499.1 | 8E-107 |
| 1712 | 2 | Adenine phosphoribosyl transferases | yes | yes | - |  |  |
| 1733 | 2 | Mitochondrial import inner membrane translocase, subunit TIM13 | no | no | - |  |  |
| 2104 | 2 | Nuclear transport factor 2 | yes | yes | AlNc14C160G7771.1 (325188077) | XP_002895567.1 | 2E-38 |
| 3330 | 2 | Transport protein particle (TRAPP) complex subunit | yes | yes | AlNc14C2073G13148.1* | XP_002899641.1 | 1E-38 |
| 3479 | 2 | Mitochondrial import inner membrane translocase, subunit TIM9 | no | no | - |  |  |
| 1688 | 3 | Golgi proteins involved in ER retention (RER) | yes | yes | AlNc14C184G8296.1 (325188668) | XP_002897246.1 | 5E-69 |
| 1769 | 3 | Ubiquitin-like proteins | yes | yes | AlNc14C243G9509.1 (325190026) | XP_002903234.1 | 4E-34 |
| 2825 | 3 | Putative arsenite-translocating ATPase | yes | yes | AlNc14C1545G12986.1* | XP_002896634.1 | 8E-125 |
| 3432 | 3 | Vacuolar H+-ATPase V1 sector, subunit F | yes | yes | AlNc14C94G5813.1 (325185946) | XP_002900892.1 | 1E-50 |
