# Supplementary material for: Gene Gain and Loss during Evolution of Obligate Parasitism in the White Rust Pathogen of Arabidopsis thaliana
Source: PLoS Biol. 2011 Jul 5;9(7):e1001094. doi: 10.1371/journal.pbio.1001094 (PMC3130010; doi:10.1371/journal.pbio.1001094)
Supplement: Table S3 — Primer pairs used to validate genome continuity and accuracy. Genomic regions were selected and PCR amplified. The first column gives the primer name and orientation, the second column, primer sequence, the third column, expected length of the PCR product, and the last column indicates if the region could be amplified or not. (DOC) [file pbio.1001094.s013.doc]

| Primer name | Primer sequence | PCR product length | Amplified |
| --- | --- | --- | --- |
| EK_1002_56F | CTCGACCGCAATCATTCAG | 1503 | yes |
| EK_1002_56R | TGAGCCTCTGCACAAAGAGA |  |  |
| EK_1024_69F | GATTGGGAGAAATGCACACC | 1728 | yes |
| EK_1024_69R | CAAGTGCCTCATTGGGCTA |  |  |
| EK_1079_91F | GGCACTACCTTGACTGGTTCA | 879 | yes |
| EK_1079_91R | CCACGTTTGGGTTTTCGTAG |  |  |
| EK_1196_62F | AGCGGAATTTCACCAAAAGA | 2064 | yes |
| EK_1196_62R | ATTCCCGCACTTTATGTTGC |  |  |
| EK_120_69F | AAGTTGCGATCCATTCATCA | 2325 | yes |
| EK_120_69R | TCATATGATTGGCCAAAATCC |  |  |
| EK_13973_19F | ATGTCCAGCCAATCAAAAGC | 447 | yes |
| EK_13973_19R | GCACCCATTTTGCCAATACT |  |  |
| EK_14094_14F | CCAAACGCGCTTTTTAATTG | 1691 | yes |
| EK_14094_14R | CATGCATCCCTTTGGAAGTT |  |  |
| EK_1511_50F | AGCGGAGCAAAGGATCAATA | 796 | yes |
| EK_1511_50R | GCAAGGATCCATCTGCAAGT |  |  |
| EK_16101_7F | CCTCTCCACTGTTTTCTCACG | 776 | yes |
| EK_16101_7R | AATGCGTCATTTCAAGCAAA |  |  |
| EK_1800_79F | AGCGAATCGAATTCAACCAA | 1257 | yes |
| EK_1800_79R | CGGAGAATGAGGCTGTCTCT |  |  |
| EK_1882_35F | TAGCACTTCCACCGCCTAAC | 671 | yes |
| EK_1882_35R | ACCTCTCATCTGCCCAACTG |  |  |
| EK_2118_5F | CGTACTCGACCATTCATACCG | 3811 | yes |
| EK_2118_5R | ATGGGTGTAGGGTAGCGTGA |  |  |
| EK_2152_36F | TTCGCAATTCGCATACTTTC | 1284 | yes |
| EK_2152_36R | TGACTATCCGGCTGAGAAGC |  |  |
| EK_2444_55F | CGTTGTTACTGGCCCTTGAT | 993 | no |
| EK_2444_55R | TCCGCTCACATTTGTCAATC |  |  |
| EK_2452_58F | CCAACGATATCGGAGCTGTT | 1131 | yes |
| EK_2452_58R | TGGGTTTTATCAATGCTTTTCC | |  |
| EK_2161_113F | CGGCTCTGCTCATCGATTTA | 769 | yes |
| EK_2161_113R | GCAAAGAGCGAACTGCAAA |  |  |
| EK_2662_23F | TCAATTCGCACTGCTCAAAG | 1083 | yes |
| EK_2662_23R | ATTGCCTAAAAGCGGGATTC |  |  |
| EK_2697_29F | TTCCTCTGCTCTTTCCATGTT | 1437 | yes |
| EK_2697_29R | TGAAAATGTTGAAATCGAAGGA | |  |
| EK_312_32F | TACACTGGTTGCCGCACAT | 1006 | yes |
| EK_312_32R | TGCAGTCGTTTGTACGATGG |  |  |
| EK_3164_58F | TTCATCAACAGAACAAGTCGAAA | 1188 | yes |
| EK_3164_58R | AAGCTGAAAAGAGTTCTACCAAGC | |  |
| EK_3172_83F | GGAAGAAAAGGGAGCGAAAC | 755 | yes |
| EK_3172_83R | ATGAATTCTGGCTGGAGCTG |  |  |
| EK_3225_7F | TCATTGCGCTCAGATTGTGT | 1429 | yes |
| EK_3225_7R | TGCATACTATGTGGTCAAGAACG | |  |
| EK_326_140F | TGGGTGTGAATCCCGATTAT | 5144 | yes |
| EK_326_140R | TGCACTCCTTTTTGCCTTCTA |  |  |
| EK_1156_136F | GGTCTTAGTCGGGCAAACCT | 2524 | yes |
| EK_1156_136R | TCCAGCCTTAGGTCAACCAG |  |  |
| EK_491_32F | GACTGAAGATCGTGCGGTTT | 1445 | yes |
| EK_491_32R | CAATGAATCTTGGCGACCTT |  |  |
| EK_1216_88F | AGAAGTGATAGTGGAGTGGAACG | 1335 | yes |
| EK_1216_88R | TGCTTGTGCAAACGAATCTT |  |  |
| EK_640_34F | CGCAAATTCGACTCCACATA | 1174 | yes |
| EK_640_34R | TTCAAAGTTCAAATGCCTTACG | |  |
| EK_4869_24F | AAGGGGGAATCCAAAGTACG | 1895 | yes |
| EK_4869_24R | TTGGTAGGAAACAGACAATTCG | |  |
| EK_2709_40F | GCCGTCGAATACGAAGAGAA | 1496 | yes |
| EK_2709_40R | TGCCTTTCATGCAACGATTA |  |  |
| EK_3435_43F | ACCCACATCACTTAGAACAATCAA | 1124 | yes |
| EK_3435_43R | TTCCAGCCTCTACATTGAATAAA | |  |
| EK_2152_58F | TCGAACATTGTGGCATGTTT | 617 | yes |
| EK_2152_58R | TGATATATCCGACCGCAAATG |  |  |
| EK_561_11F | CTGCGAGCGGTGCTATTG | 1300 | yes |
| EK_561_11R | TGTTGCTCTTGATTCGTTTCC |  |  |
