# Supplementary material for: Gene Gain and Loss during Evolution of Obligate Parasitism in the White Rust Pathogen of Arabidopsis thaliana
Source: PLoS Biol. 2011 Jul 5;9(7):e1001094. doi: 10.1371/journal.pbio.1001094 (PMC3130010; doi:10.1371/journal.pbio.1001094)
Supplement: Table S4 — Repetitive elements in the A. laibachii assembly. After a search of the library generated with RepeatScout for sequences homologous to transposons, we identified 270 consensus elements showing significant similarity to known transposons. The most abundant in the genome were mariner (DNA transposon) and copia (LTR retrotransposon) elements. Consensus repeats that do not match any deposited in the NCBI database and do not overlap with Nc14 protein coding genes are either Albugo-specific repeats (light grey background) or simple repeats. We identified 191 such consensus sequences that compose about 1% of the assembly. (DOC) [file pbio.1001094.s014.doc]

| **Name of transposon** | **Total length in the genome, bp** | **Proportion in the genome, %** |
| --- | --- | --- |
| ***Mariner***  DNA transposon | 2514560 | 7.7 |
| ***Copia***  LTR retrotransposon | 1895041 | 5.8 |
| ***MuDR***  DNA transposon | 1355685 | 4.1 |
| ***Gypsy***  LTR retrotransposon | 499383 | 1.5 |
| ***L1***  non-LTR retrotransposon | 373579 | 1.1 |
| ***RTE***  non-LTR retrotransposon | 98761 | 0.3 |
| ***Harbinger***  DNA transposon | 54717 | 0.17 |
| ***hAT***  DNA transposon | 50229 | 0.15 |
| ***Albugo specific repeats*** | 280660 | 0.8 |
| ***Simple repeats*** | 33042 | 0.1 |
| ***Telomeric repeats*** | 5925 | 0.02 |
| **Total** | **7161582** | **21.8** |
