# Supplementary material for: Gene Gain and Loss during Evolution of Obligate Parasitism in the White Rust Pathogen of Arabidopsis thaliana
Source: PLoS Biol. 2011 Jul 5;9(7):e1001094. doi: 10.1371/journal.pbio.1001094 (PMC3130010; doi:10.1371/journal.pbio.1001094)
Supplement: Table S5 — Distribution of repetitive elements relative to contig length. Out of the total 3,816 contigs in the assembly, 2,211 contigs have regions with similarity to transposons or other repetitive sequences. Most of these contigs (1,528 contigs) are less than 5,000 bp long. (DOC) [file pbio.1001094.s015.doc]

|  | **Number of short contigs (<= 5kb)** | **Number of long contigs**  **(> 5kb)** |
| --- | --- | --- |
| *Assembly in total* | *3094* | *722* |
| Transposons | 1077 | 624 |
| Albugo-specific/simple repeats/telomeric repeats | 653 | 614 |
| Total repetitive elements | 1528 | 683 |
