# Supplementary material for: Gene Gain and Loss during Evolution of Obligate Parasitism in the White Rust Pathogen of Arabidopsis thaliana
Source: PLoS Biol. 2011 Jul 5;9(7):e1001094. doi: 10.1371/journal.pbio.1001094 (PMC3130010; doi:10.1371/journal.pbio.1001094)
Supplement: Table S6 — Distribution of repeats matching telomeric consensus sequences. Forward and reverse telomeric consensus sequences were identified with RepeatScout. A total of 45 contigs have repeats matching telomeric consensus sequences; amongst these, 25 contigs have telomeric repeats located either at the beginning or at the end of a contig. In all, 5,925 bp of telomeric repeats was assembled. (DOC) [file pbio.1001094.s016.doc]

| Position | Contig | Start position | End position |
| --- | --- | --- | --- |
| START of the contig | CONTIG_18_NC14_v4_198151_220  CONTIG_76_NC14_v4_95330_245  CONTIG_109_NC14_v4_76566_235  CONTIG_142_NC14_v4_63680_233  CONTIG_161_NC14_v4_57741_228  CONTIG_300_NC14_v4_31427_229  CONTIG_358_NC14_v4_24322_232  CONTIG_411_NC14_v4_20238_236  CONTIG_836_NC14_v4_5486_437  CONTIG_886_NC14_v4_4920_349  CONTIG_936_NC14_v4_4597_652  CONTIG_1152_NC14_v4_3274_980  CONTIG_1217_NC14_v4_2958_608 | 1  7  1  1  1  7  1  1  1  1  1  2  1 | 278  407  250  171  79  209  60  200  59  59  110  100  65 |
| END of the contig | CONTIG_2_NC14_v4_395950_240  CONTIG_127_NC14_v4_70157_159  CONTIG_133_NC14_v4_68809_223  CONTIG_180_NC14_v4_52563_236  CONTIG_425_NC14_v4_18806_221  CONTIG_530_NC14_v4_12259_223  CONTIG_565_NC14_v4_11071_245  CONTIG_820_NC14_v4_5688_278  CONTIG_1045_NC14_v4_3806_1029  CONTIG_2047_NC14_v4_1153_1168  CONTIG_2370_NC14_v4_844_490  CONTIG_3788_NC14_v4_312_519 | 395786  70045  68570  52488  18747  12195  10939  5602  3596  1045  786  235 | 395950  70156  68809  52563  18806  12259  11071  5688  3806  1153  844  312 |
| Most of the contig - repeat | CONTIG_3949_NC14_v4_289_386  CONTIG_4055_NC14_v4_278_240  CONTIG_4557_NC14_v4_216_456  CONTIG_4744_NC14_v4_176_416  CONTIG_4757_NC14_v4_172_583  CONTIG_4575_NC14_v4_212_381  CONTIG_4828_NC14_v4_162_619  CONTIG_4985_NC14_v4_126_236  CONTIG_4950_NC14_v4_142_293  CONTIG_4962_NC14_v4_136_425  CONTIG_4975_NC14_v4_129_191  CONTIG_4982_NC14_v4_126_786  CONTIG_5005_NC14_v4_121_1193 | 1  1  1  1  2  1  1  1  1  1  1  2  1 | 278  267  197  127  170  162  162  74  142  136  129  126  121 |
| Other locations | CONTIG_48_NC14_v4_120044_121  CONTIG_50_NC14_v4_115337_117  CONTIG_144_NC14_v4_63583_160  CONTIG_272_NC14_v4_35091_229  CONTIG_606_NC14_v4_9712_428  CONTIG_112_NC14_v4_75306_234  CONTIG_507_NC14_v4_13379_204 | 6639  114075  61724  31712  9320  66229  4767 | 6694  114130  61791  31800  9384  66300  4821 |
