# Supplementary material for: Gene Gain and Loss during Evolution of Obligate Parasitism in the White Rust Pathogen of Arabidopsis thaliana
Source: PLoS Biol. 2011 Jul 5;9(7):e1001094. doi: 10.1371/journal.pbio.1001094 (PMC3130010; doi:10.1371/journal.pbio.1001094)
Supplement: Table S7 — Characterisation of tRNA genes in the assembled A. laibachii contigs. Type of tRNA gene, number of genes (without and with introns), number of anticodons, type of anticodon, and frequency of usage as a number of stars; 15 tRNA genes were predicted with introns. (DOC) [file pbio.1001094.s017.doc]

| ***tRNA*** | ***Genes without introns*** | ***Genes with introns*** | ***Number of anticodons*** |
| --- | --- | --- | --- |
| tRNA-Pro | 7 | 0 | 3  cgg*, tgg*****, agg* |
| tRNA-Gln | 5 | 2 | 2  ctg**, ttg***** |
| tRNA-Leu | 11 | 2 | 5  caa****, taa**, tag**, cag*, aag**** |
| tRNA-Asn | 5 | 0 | 1  gtt* |
| tRNA-Asp | 6 | 0 | 1  gtc* |
| tRNA-Ser | 10 | 4 | 4  cga*, aga**, gct********, tga*** |
| tRNA-His | 5 | 0 | 1  gtg* |
| tRNA-Glu | 7 | 0 | 2  ttc*****, ctc** |
| tRNA-Met | 10 | 0 | 1  cat********** |
| tRNA-Arg | 13 | 1 | 6  cct**, gcg**, acg****, ccg*, tcg**, tct *** |
| tRNA-Phe | 5 | 0 | 1  gaa* |
| tRNA-Lys | 8 | 2 | 2  ctt****, ttt****** |
| tRNA-Cys | 4 | 1 | 1  gca* |
| tRNA-Thr | 6 | 0 | 3  tgt**, cgt*, agt*** |
| tRNA-Trp | 2 | 0 | 1  cca* |
| tRNA-Tyr | 3 | 0 | 1  gta* |
| tRNA-Ala | 9 | 0 | 3  tgc*****, agc**, cgc** |
| tRNA-Val | 6 | 1 | 3  aac***, cac**, tac** |
| tRNA-Gly | 10 | 2 | 4  gcc*****, ccc*, acc*, tcc***** |
| tRNA-Ile | 6 | 0 | 3  aat***, gat**, tat* |
| **TOTAL** | **138** | **15** |  |
