# Supplementary material for: Gene Gain and Loss during Evolution of Obligate Parasitism in the White Rust Pathogen of Arabidopsis thaliana
Source: PLoS Biol. 2011 Jul 5;9(7):e1001094. doi: 10.1371/journal.pbio.1001094 (PMC3130010; doi:10.1371/journal.pbio.1001094)
Supplement: Table S8 — Annotations for the brassinosteroid biosynthesis pathway. The first column gives the enzyme commission numbers (EC numbers) of possible genes. The second column indicates gene names in Ar. thaliana. Question marks indicate genes that are difficult to annotate for a certain function (genes that belong to the superfamily of cytochrome P450s). The third column indicates genes identified using the ASGARD annotation pipeline, and the fourth column indicates manual annotation. (GI numbers in brackets.) (DOC) [file pbio.1001094.s018.doc]

| EC | Corresponding gene in *A. thaliana* | ASGARD annotation | manual annotation |
| --- | --- | --- | --- |
| 1.14.13.59 | Dwf4 | AlNc14C22G2282.1 (325182055) | AlNc14C22G2282.1 (325182055) |
| 1.14.99.29 | Br6ox (?) | AlNc14C91G5694.1 (325185818) | AlNc14C130G6945.1  (325187155) |
| 1.14.99.3 | Br6ox (?) | AlNc14C154G7595.1 (325187876) | nd |
| 1.14.19.1 | D2 / CPD (?) | AlNc14C32G2939.1 (325182787) | AlNc14C22G2282.1 (325182055) |
| 1.14.11.8 | D2 / CPD (?) | AlNc14C398G11346.1 (325192192) | AlNc14C22G2282.1 (325182055) |
| 1.3.99.5 | DET2 | AlNc14C153G7565.1 (325187847) | AlNc14C153G7565.1 (325182055) |
