# Supplementary material for: Gene Gain and Loss during Evolution of Obligate Parasitism in the White Rust Pathogen of Arabidopsis thaliana
Source: PLoS Biol. 2011 Jul 5;9(7):e1001094. doi: 10.1371/journal.pbio.1001094 (PMC3130010; doi:10.1371/journal.pbio.1001094)
Supplement: Table S9 — Potentially green-alga-derived genes that were identified based on results of a set of green- and red-alga-derived genes present in the diatom Ph. tricornutum . Genes listed here had to be present in the green alga Ch. reinhardtii (chloroplast or nuclear genome) but had to be absent from the red alga C. merolae and from the fungi F. oxysporum and U. maydis. (Orange: in A. laibachii, P. infestans, Py. ultimum, Ph. tricornutum, Th. pseudonana, Ch. reinhardtii, and Pl. falciparum but not in H. arabidopsidis and E. siliculosus. Brown: as before but in E. siliculosus. Green: shared at least between Pl. falciparum and oomycetes. Annotations for identified genes were taken from the list published by Moustafa et al. [37].) (DOC) [file pbio.1001094.s019.doc]

| A. laibachii gene | GI number (*A. laibachii* genes) | GI number (best hit matching the *P. tricornutum* gene [37]) | Annotation (from [37]) |
| --- | --- | --- | --- |
| AlNc14C37G3260.1 | 325183138 | 219113565 | Adenylyl cyclase (ISS) |
| AlNc14C37G3260.1 | 325183138 | 219113401 | Adenylyl cyclase (ISS) |
| AlNc14C33G2991.1 | 325182842 | 219113401 | Adenylyl cyclase (ISS) |
| AlNc14C40G3433.1 | 325183330 | 219121324 | AGAP010200‐PA (Fragment) |
| AlNc14C38G3311.1 | 325183199 | 219110611 | Aspartic protease PM5 |
| AlNc14C38G3310.1 | 325183198 | 219110611 | Aspartic protease PM5 |
| AlNc14C261G9819.1 | 325190367 | 219110611 | Aspartic protease PM5 |
| AlNc14C186G8327.1 | 325188700 | 219110611 | Aspartic protease PM5 |
| AlNc14C60G4422.1 | 325184469 | 219110611 | Aspartic protease PM5 |
| AlNc14C9G1195.1 | 325180819 | 219111733 | Cathepsin Z (ISS) |
| AlNc14C18G1899.1 | 325181665 | 219111733 | Cathepsin Z (ISS) |
| AlNc14C281G10113.1 | 325190706 | 219111733 | Cathepsin Z (ISS) |
| AlNc14C55G4233.1 | 325184271 | 219111733 | Cathepsin Z (ISS) |
| AlNc14C9G1195.1 | 325180819 | 219111735 | Cathepsin Z (ISS) |
| AlNc14C18G1899.1 | 325181665 | 219111735 | Cathepsin Z (ISS) |
| AlNc14C281G10113.1 | 325190706 | 219111735 | Cathepsin Z (ISS) |
| AlNc14C55G4233.1 | 325184271 | 219111735 | Cathepsin Z (ISS) |
| AlNc14C9G1195.1 | 325180819 | 219111779 | Cathepsin Z (ISS) |
| AlNc14C18G1899.1 | 325181665 | 219111779 | Cathepsin Z (ISS) |
| AlNc14C281G10113.1 | 325190706 | 219111779 | Cathepsin Z (ISS) |
| AlNc14C55G4233.1 | 325184271 | 219111779 | Cathepsin Z (ISS) |
| AlNc14C9G1195.1 | 325180819 | 219111781 | Cathepsin Z (ISS) |
| AlNc14C18G1899.1 | 325181665 | 219111781 | Cathepsin Z (ISS) |
| AlNc14C281G10113.1 | 325190706 | 219111781 | Cathepsin Z (ISS) |
| AlNc14C55G4233.1 | 325184271 | 219111781 | Cathepsin Z (ISS) |
| AlNc14C206G8818.1 | 325189248 | 219111805 | Chromosome chr11 scaffold_13 |
| AlNc14C62G4481.1 | 325184531 | 219111363 | Chromosome chr19 scaffold_4 |
| AlNc14C657G12347.1 | 325193402 | 219117946 | Chromosome chr9 scaffold_49 |
| AlNc14C247G9577.1 | 325190099 | 219117946 | Chromosome chr9 scaffold_49 |
| AlNc14C113G6444.1 | 325186596 | 219127275 | Chromosome undetermined scaffold_68 |
| AlNc14C33G2991.1 | 325182842 | 219121289 | Chromosome undetermined scaffold_73 |
| AlNc14C37G3260.1 | 325183138 | 219121289 | Chromosome undetermined scaffold_73 |
| AlNc14C254G9695.1 | 325190233 | 219123575 | Diacylglycerol kinase 1 |
| AlNc14C11G1326.1 | 325180973 | 219127616 | Expressed protein |
| AlNc14C3G440.1 | 325179931 | 219119237 | F1N19.9 (26S proteasome subunit RPN12) (At1g64520/F1N19_10) |
| AlNc14C165G7866.1 | 325188181 | 219114987 | GRF zinc finger family protein (Os12g0143100 protein) |
| AlNc14C54G4152.1 | 325184184 | 219118724 | Lipoate protein ligase‐like protein |
| AlNc14C221G9109.1 | 325189598 | 219129505 | MA3 protein |
| AlNc14C140G7227.1 | 325187462 | 219117183 | MORN repeat protein |
| AlNc14C65G4634.1 | 325184695 | 219117183 | MORN repeat protein |
| AlNc14C85G5448.1 | 325185564 | 219117183 | MORN repeat protein |
| AlNc14C6G875.1 | 325180428 | 219129981 | Os04g0542900 protein |
| AlNc14C16G1748.1 | 325181448 | 219119700 | Predicted protein |
| AlNc14C184G8288.1 | 325188659 | 219119700 | Predicted protein |
| AlNc14C16G1748.9 | 325181448 | 219119700 | Predicted protein |
| AlNc14C92G5738.1 | 325185868 | 219124724 | Predicted protein |
| AlNc14C15G1715.1 | 325181409 | 219110983 | Probable aldehyde dehydrogenase (EC 1.2.1.3) (Flax‐inducible sequence 1) |
| AlNc14C291G10237.1 | 325190843 | 219116456 | Putative tetratricopeptide repeat protein |
| AlNc14C202G8721.1 | 325189145 | 219116456 | Putative tetratricopeptide repeat protein |
| AlNc14C247G9577.1 | 325190099 | 219118433 | Putative uncharacterized protein |
| AlNc14C657G12347.1 | 325193402 | 219118433 | Putative uncharacterized protein |
| AlNc14C4G553.1 | 325180081 | 219118433 | Putative uncharacterized protein |
| AlNc14C6G854.1 | 325180399 | 219122903 | Putative uncharacterized protein pkci (Fragment) |
| AlNc14C18G1883.1 | 325181646 | 219124674 | PV72 |
| AlNc14C110G6377.1 | 325186519 | 219124674 | PV72 |
| AlNc14C49G3917.1 | 325183929 | 219112273 | Ran binding 16 homologue |
| AlNc14C39G3392.1 | 325183285 | 219112273 | Ran binding 16 homologue |
| AlNc14C400G11360.1 | 325192208 | 219117429 | Ribokinase (ISS) |
| AlNc14C142G7298.1 | 325187534 | 219130448 | Saccharopine dehydrogenase |
| AlNc14C38G3308.1 | 325183191 | 219120550 | Solute carrier family 35 |
| AlNc14C110G6359.1 | 325186501 | 219126900 | SpoU protein |
| AlNc14C6G875.1 | 325180428 | 219129979 | Zgc:110053 |
| AlNc14C24G2410.1 | 325182200 | 219112301 | Zgc:163002 protein |
