# Supplementary material for: Gene Gain and Loss during Evolution of Obligate Parasitism in the White Rust Pathogen of Arabidopsis thaliana
Source: PLoS Biol. 2011 Jul 5;9(7):e1001094. doi: 10.1371/journal.pbio.1001094 (PMC3130010; doi:10.1371/journal.pbio.1001094)
Supplement: Table S10 — Green alga genes showing homology to A. laibachii genes but not to diatome, red alga, brown alga, or fungal genes. Genes listed here had to be present in the green algae Ch. reinhardtii (chloroplast or nuclear genome) and V. carteri but had to be absent from the red alga C. merolae, the fungi F. oxysporum and U. maydis, and the brown alga E. siliculosus (for the BLAST analyses, an e-value cut-off of 1e−20 was used; proteins retained by repeating the analyses using an e-value cut-off of 1e−5 are indicated in blue). (DOC) [file pbio.1001094.s020.doc]

| A. laibachii gene | GI number (*A. laibachii* genes) | GI number for best NCBI BLAST hit | annotation |
| --- | --- | --- | --- |
| AlNc14C2G277.1 | 325179766 | XP_002906836 | 2,3-bisphosphoglycerate-independent phosphoglycerate mutase, putative |
| AlNc14C128G6885.1 | 325187091 | XP_002900811 | biotin-protein ligase , putative |
| AlNc14C620G12262.1 | 325193313 | XP_002897350 | carbohydrate esterase , putative |
| AlNc14C107G6246.1 | 325186412 | XP_002906650 | conserved hypothetical protein |
| AlNc14C17G1802.1 | 325181535 | XP_002906410 | conserved hypothetical protein |
| AlNc14C223G9157.1 | 325189648 | XP_002907908 | conserved hypothetical protein |
| AlNc14C28G2728.1 | 325182550 | XP_002998268 | conserved hypothetical protein |
| AlNc14C297G10326.1 | 325190948 | XP_002895421 | conserved hypothetical protein |
| AlNc14C31G2871.1 | 325182715 | XP_002896418 | conserved hypothetical protein |
| AlNc14C31G2877.1 | 325182721 | XP_002905652 | conserved hypothetical protein |
| AlNc14C3G376.1 | 325179866 | XP_002904642 | conserved hypothetical protein |
| AlNc14C3G393.1 | 325179883 | XP_002905403 | conserved hypothetical protein |
| AlNc14C46G3732.1 | 325183720 | XP_002907782 | conserved hypothetical protein |
| AlNc14C68G4784.1 | 325184849 | XP_002904997 | conserved hypothetical protein |
| AlNc14C73G4988.1 | 325185070 | XP_002899945 | conserved hypothetical protein |
| AlNc14C87G5539.1 | 325185660 | XP_002904152 | conserved hypothetical protein |
| AlNc14C89G5621.1 | 325185746 | XP_002906618 | conserved hypothetical protein |
| AlNc14C928G12641.1 | 325193763 | XP_002904939 | conserved hypothetical protein |
| AlNc14C25G2494.1 | 325182296 | XP_002897113 | Di-N-acetylchitobiase , putative |
| AlNc14C40G3410.1 | 325183307 | XP_002900250 | dolichyl-diphosphooligosaccharide-protein glycosyltransferase subunit , putative |
| AlNc14C12G1437.1 | 325181087 | XP_002898120 | guanylate-binding protein , putative |
| AlNc14C82G5339.1 | 325185448 | XP_002902498 | guanylate-binding protein , putative |
| AlNc14C62G4519.1 | 325184573 | XP_002899326 | leishmanolysin-like peptidase , putative |
| AlNc14C100G6003.1 | 325186156 | XP_002902195 | nitric oxide synthase-interacting protein , putative |
| AlNc14C167G7918.1 | 325188235 | XP_002898691 | phosphate acetyltransferase , putative |
| AlNc14C192G8477.1 | 325188871 | XP_002505328 | predicted protein , putative |
| AlNc14C27G2645.1 | 325182456 | XP_002504522 | predicted protein , putative |
| AlNc14C166G7882.1 | 325188197 | XP_002904828 | riboflavin kinase , putative |
| AlNc14C49G3908.1 | 325183917 | XP_002904773 | serine protease family S33 , putative |
| AlNc14C2G274.1 | 325179763 | XP_002906835 | splicing factor , putative |
| AlNc14C571G12180.1 | 325193196 | XP_002904058 | translation initiation factor eIF-2B subunit gamma , putative |
| AlNc14C99G5966.1 | 325186115 | XP_002909148 | ubiquinone biosynthesis protein COQ9 , putative |
