# Supplementary material for: Gene Gain and Loss during Evolution of Obligate Parasitism in the White Rust Pathogen of Arabidopsis thaliana
Source: PLoS Biol. 2011 Jul 5;9(7):e1001094. doi: 10.1371/journal.pbio.1001094 (PMC3130010; doi:10.1371/journal.pbio.1001094)
Supplement: Table S11 — Red alga genes showing homology to A. laibachii genes but not to diatom, green algae, brown alga, or fungal genes. Genes listed here had to be present in the red algae C. merolae and G. sulphuraria but had to be absent from the green algae Ch. reinhardtii (chloroplast or nuclear genome) and V. carteri, the fungi F. oxysporum and U. maydis, and the brown alga E. siliculosus (for the BLAST analyses, an e-value cut-off of 1e−20 was used; proteins retained by repeating the analyses using an e-value cut-off of 1e−5 are indicated in blue). (DOC) [file pbio.1001094.s021.doc]

| A. laibachii gene | GI number (*A. laibachii* genes) | GI number for best NCBI BLAST hit | annotation |
| --- | --- | --- | --- |
| AlNc14C313G10517.1 | 325191745 | XP_002997459 | conserved hypothetical protein |
| AlNc14C230G9279.1 | 325189779 | XP_002997618 | conserved hypothetical protein |
| AlNc14C35G3137.1 | 325183012 | XP_002907194 | conserved hypothetical protein |
| AlNc14C419G11509.1 | 325192379 | XP_002905302 | formin-homology 2 domain-containing protein , putative |
| AlNc14C158G7708.1 | 325188011 | XP_002909280 | isoamyl acetate-hydrolyzing esterase 1 , putative |
| AlNc14C56G4252.1 | 325184291 | XP_002900469 | phosphatidylinositol transfer protein beta isoform , putative |
| AlNc14C192G8476.1 | 325188870 | XP_002909640 | ras family GTPase , putative |
| AlNc14C47G3781.1 | 325183780 | XP_002906015 | ribosome biogenesis regulatory protein , putative |
| AlNc14C13G1552.1 | 325181219 | XP_002999167 | RNA pseudouridylate synthase , putative |
| AlNc14C105G6169.1 | 325186333 | XP_002908624 | SWI/SNF-related matrix-associated actin-dependent regulator of chromatin , putative |
| AlNc14C368G11070.1 | 325191902 | XP_002897356 | tRNA guanosine-2'-O-methyltransferase , putative |
