# Supplementary material for: Gene Gain and Loss during Evolution of Obligate Parasitism in the White Rust Pathogen of Arabidopsis thaliana
Source: PLoS Biol. 2011 Jul 5;9(7):e1001094. doi: 10.1371/journal.pbio.1001094 (PMC3130010; doi:10.1371/journal.pbio.1001094)
Supplement: Table S12 — Potentially green-alga-derived genes that are present in the diatoms Ph. tricornutum and Th. pseudonana but not in A. laibachii Nc14. Genes listed here had to be present in the green alga Ch. reinhardtii (chloroplast or nuclear genome) but had to be absent from A. laibachii Nc14, the red alga C. merolae, and the fungi F. oxysporum and U. maydis. Columns 3–7 show presence/absence in Py. ultimum, P. infestans, H. arabidopsidis, Pl. falciparum, and E. siliculosus using the same criteria. (a, absent; p, present. Annotations for identified genes were taken from the list published by Moustafa et al. [33].) (DOC) [file pbio.1001094.s022.doc]

| GI number  (*T. pseudonana* genes) | Annotation (*T. pseudonana* genes) | *P. ultimum* | *P. infestans* | *H. arabidopsidis* | *P. falciparum* | *E. siliculosus* |
| --- | --- | --- | --- | --- | --- | --- |
| gi|224014066 | 6‐phosphogluconolactonase‐like protein (ISS) | a | a | a | a | a |
| gi|219115701 | ABC(Binding protein) family transporter: phosphate | a | a | a | a | a |
| gi|224002016 | ABC(Binding protein) family transporter: phosphate | a | a | a | a | a |
| gi|219113517 | Acetylpolyamine aminohydolase | a | a | a | a | a |
| gi|219112585 | AGAP000194‐PA | a | a | a | a | p |
| gi|219111791 | Arabidopsis thaliana genomic DNA,chromosome 3, P1 clone: MOE17 | a | a | a | a | p |
| gi|224000784 | Ataxin 3 | a | a | a | a | p |
| gi|219121792 | Ataxin‐3 (EC 3.4.22.‐)(Machado‐Joseph disease protein 1 homolog) | a | a | a | a | p |
| gi|224005248 | Chromosome 01 contig 1, DNA sequence | a | a | a | a | a |
| gi|219123757 | Chromosome 02 contig 1, DNA sequence | a | a | a | a | p |
| gi|219128011 | Chromosome 04 contig 1, DNA sequence | a | a | a | a | p |
| gi|224005803 | Chromosome 04 contig 1, DNA sequence | a | a | a | a | p |
| gi|219120159 | Chromosome 05 contig 1, DNA sequence | a | a | a | a | a |
| gi|219125461 | Chromosome 05 contig 1, DNA sequence | a | a | a | a | p |
| gi|219121668 | Chromosome 14 contig 1, DNA sequence | a | a | a | a | p |
| gi|224011066 | Chromosome 14 contig 1, DNA sequence | a | a | a | a | p |
| gi|224010870 | Chromosome chr13 scaffold_210, whole genome shotgun sequence | p | p | p | a | p |
| gi|219122997 | Chromosome chr13 scaffold_45, whole genome shotgun sequence | a | a | a | a | a |
| gi|219130926 | Chromosome chr13 scaffold_45, whole genome shotgun sequence | a | a | a | a | a |
| gi|219127508 | Chromosome chr14 scaffold_27, whole genome shotgun sequence | a | a | p | a | a |
| gi|219128946 | Chromosome chr14 scaffold_9, whole genome shotgun sequence | a | a | a | a | p |
| gi|224015147 | Chromosome chr14 scaffold_9, whole genome shotgun sequence | a | a | a | a | p |
| gi|219120413 | Chromosome chr15 scaffold_40, whole genome shotgun sequence | a | a | a | a | a |
| gi|219127878 | Chromosome chr17 scaffold_16, whole genome shotgun sequence | a | a | a | a | p |
| gi|224013208 | Chromosome chr2 scaffold_105, whole genome shotgun sequence | a | a | a | a | p |
| gi|224002142 | Chromosome chr3 scaffold_8, whole genome shotgun sequence | a | a | a | a | a |
| gi|219123980 | Chromosome chr3 scaffold_8, whole genome shotgun sequence | a | a | a | a | p |
| gi|223993155 | Chromosome chr3 scaffold_8, whole genome shotgun sequence | a | a | a | a | p |
| gi|224007901 | Chromosome chr3 scaffold_8, whole genome shotgun sequence | a | a | a | a | p |
| gi|219123065 | Chromosome chr5 scaffold_2, whole genome shotgun sequence | a | a | a | a | a |
| gi|219124147 | Chromosome chr5 scaffold_2, whole genome shotgun sequence | a | p | a | a | p |
| gi|224002132 | Chromosome chr5 scaffold_2, whole genome shotgun sequence | a | p | a | a | p |
| gi|224003533 | Chromosome chr5 scaffold_2, whole genome shotgun sequence | a | a | a | a | p |
| gi|219116568 | Chromosome chr6 scaffold_3, whole genome shotgun sequence | a | a | a | a | p |
| gi|219111523 | Chromosome chr7 scaffold_20, whole genome shotgun sequence | a | a | a | a | p |
| gi|219109820 | Chromosome chr7 scaffold_42, whole genome shotgun sequence | a | a | a | a | p |
| gi|223994801 | Chromosome chr7 scaffold_42, whole genome shotgun sequence | a | a | a | a | p |
| gi|224011006 | Chromosome chr8 scaffold_106, whole genome shotgun sequence | a | a | a | a | a |
| gi|224010267 | Chromosome undetermined SCAF14565,whole genome shotgun sequence. | p | p | p | a | p |
| gi|219123139 | Chromosome undetermined scaffold_87,whole genome shotgun sequence | a | a | a | a | p |
| gi|219113165 | COG1028: Dehydrogenases with different specificities (ISS) | a | a | a | a | p |
| gi|224000585 | COG3781: Predicted membrane protein(ISS) | a | a | a | a | a |
| gi|219118943 | CpABA1 protein | a | a | a | a | p |
| gi|219111765 | Cytochrome P450 hydroxylase | a | a | a | a | a |
| gi|219113827 | Cytochrome P450 | a | a | a | a | p |
| gi|219115065 | Cytochrome P450 | a | a | a | a | p |
| gi|219114867 | D‐isomer specific 2‐hydroxyacid dehydrogenase, NAD‐binding | a | a | a | a | p |
| gi|219121407 | DBH‐like monooxygenase protein 1 precursor (EC 1.14.17.‐) | a | a | a | a | p |
| gi|219130703 | Delta 12 fatty acid desaturase FAT‐2 (Putative uncharacterized protein fat‐ | a | a | a | a | p |
| gi|224002771 | Delta 12 fatty acid desaturase FAT‐2 (Putative uncharacterized protein fat‐ | p | p | p | a | p |
| gi|224000772 | Delta‐11 fatty acid desaturase | a | a | a | a | p |
| gi|224005637 | Digalactosyldiacylglycerol synthase 1 | a | a | a | a | p |
| gi|223993947 | Expressed protein (Os03g0109700 protein) | a | a | a | a | p |
| gi|219122834 | Expressed protein (Os03g0137600 protein) | a | a | a | a | a |
| gi|224010159 | Expressed protein (Putative uncharacterized protein At2g21960) | a | a | a | a | a |
| gi|219119472 | Ferredoxin nitrite reductase chlo Red | a | p | a | a | p |
| gi|223999185 | Ferredoxin nitrite reductase chlo Red | a | p | a | a | p |
| gi|224013062 | Fucoxanthin chlorophyll a/c binding protein precursor (Fragment) | a | a | a | a | p |
| gi|224002166 | Fucoxanthin chlorophyll a/c binding protein precursor (Fragment) | a | a | a | a | p |
| gi|219117950 | Fucoxanthin chlorophyll a/c binding protein precursor | a | a | a | a | p |
| gi|219124963 | Fucoxanthin chlorophyll a/c binding protein precursor | a | a | a | a | p |
| gi|219110471 | Fucoxanthin chlorophyll a/c binding protein precursor | a | a | a | a | p |
| gi|219115808 | Fucoxanthin chlorophyll a/c binding protein precursor | a | a | a | a | p |
| gi|223994783 | Fucoxanthin chlorophyll a/c binding protein precursor | a | a | a | a | p |
| gi|224013064 | Fucoxanthin chlorophyll a/c binding protein precursor | a | a | a | a | p |
| gi|224013212 | Fucoxanthin chlorophyll a/c binding protein precursor | a | a | a | a | p |
| gi|219117455 | Gb AAC18972.1 (Putative uncharacterized protein At5g67370) | a | a | a | a | p |
| gi|219127292 | Glutathione S‐transferase (ISS)(Fragment) | a | a | a | a | a |
| gi|219118261 | GONST4 Golgi Nucleotide sugar transporter (At5g19980) (Fragment) | a | a | a | a | a |
| gi|223998396 | GONST4 Golgi Nucleotide sugar transporter (At5g19980) (Fragment) | a | a | a | a | a |
| gi|219112627 | Green | a | a | a | a | p |
| gi|219113593 | Green | a | a | a | a | p |
| gi|219113863 | Green | a | a | a | a | a |
| gi|219112197 | Haloacid dehalogenase‐like hydrolase‐like (ISS) (Fragment) other Red | a | a | a | a | p |
| gi|219119393 | Homology to unknown gene (Fragment) | a | a | a | a | p |
| gi|219111259 | Homology to unknown gene | a | a | a | a | p |
| gi|219109759 | Homology to unknown gene | a | a | a | a | p |
| gi|219124475 | Homology to unknown gene | a | a | a | a | p |
| gi|219129470 | Homology to unknown gene | a | a | a | a | a |
| gi|219118429 | Hybrid cluster protein | a | a | a | a | a |
| gi|223997498 | Hybrid cluster protein | a | a | a | a | a |
| gi|219127725 | Hypothetical conserved protein | a | a | a | a | p |
| gi|219115769 | Hypothetical isochorismatase family protein | a | a | a | a | p |
| gi|219123121 | JmjC domain‐containing protein 5(Jumonji domain‐containing protein 5) | p | p | a | a | p |
| gi|219111009 | Kelch repeat containing protein(Os09g0249000 protein) | a | a | a | a | a |
| gi|223999905 | Magnesium transporter | a | a | a | a | a |
| gi|219113509 | Metalloendopeptidase | a | a | a | a | p |
| gi|219111095 | MFS family transporter: sugar | a | a | a | a | p |
| gi|223995121 | MFS family transporter: sugar | a | a | a | a | p |
| gi|223998290 | MFS family transporter: sugar | a | a | a | a | a |
| gi|219127896 | MGC159930 protein | p | p | a | a | p |
| gi|219109876 | Mll2645 protein | a | a | a | a | a |
| gi|224007042 | Nuclear pore complex component (Sc Seh1) (ISS) | a | a | a | a | p |
| gi|219115337 | Os07g0417800 protein | a | a | a | a | a |
| gi|219115339 | Os07g0417800 protein | a | a | a | a | a |
| gi|219115347 | Os07g0417800 protein | a | a | a | a | a |
| gi|223994079 | Os09g0444700 protein (Fragment) | a | p | a | a | a |
| gi|223993267 | OSIGBa0092M08.4 protein | a | a | a | a | p |
| gi|219110026 | Peptidyl‐prolyl cis‐trans isomerase (EC 5.2.1.8) | a | a | a | a | p |
| gi|219130496 | Peptidyl‐prolyl cis‐trans isomerase (EC 5.2.1.8) | a | a | a | a | a |
| gi|224010860 | Peptidyl‐prolyl cis‐trans isomerase (EC 5.2.1.8) | a | a | a | a | p |
| gi|224010746 | Permease, putative (ISS) | a | a | a | a | p |
| gi|219115359 | Peroxisomal membrane 22 kDa family protein (ISS) | a | a | a | a | a |
| gi|219109668 | Peroxisomal membrane 22 kDa family protein (ISS) | a | a | a | a | a |
| gi|223995829 | Peroxisomal membrane 22 kDa family protein (ISS) | a | a | a | a | a |
| gi|223997712 | Peroxisomal membrane 22 kDa family protein (ISS) | a | a | a | a | a |
| gi|224006321 | Predicted NUDIX hydrolase FGF‐2 and related proteins (ISS) | a | a | a | a | p |
| gi|219115017 | Predicted protein (Fragment) | a | a | a | a | a |
| gi|223997908 | Predicted protein (Fragment) | a | a | a | a | p |
| gi|224012885 | Predicted protein (Fragment) | a | a | a | a | a |
| gi|219112087 | Predicted protein (Fragment) | p | a | a | a | p |
| gi|219116132 | Predicted protein (Fragment) | a | a | a | a | a |
| gi|219120024 | Predicted protein (Fragment) | a | a | a | a | a |
| gi|219120026 | Predicted protein (Fragment) | a | a | a | a | a |
| gi|219124574 | Predicted protein (Fragment) | a | a | a | a | p |
| gi|223999673 | Predicted protein (Fragment) | a | a | a | a | a |
| gi|219121399 | Predicted protein | a | a | a | a | a |
| gi|219127986 | Predicted protein | a | a | a | a | p |
| gi|219129999 | Predicted protein | a | a | a | a | a |
| gi|224001312 | Predicted protein | a | a | a | a | a |
| gi|224009444 | Predicted protein | a | a | a | a | a |
| gi|224012188 | Predicted protein | p | a | a | a | a |
| gi|219116066 | Predicted protein | a | a | a | a | a |
| gi|219111375 | Predicted protein | a | a | a | a | p |
| gi|219113307 | Predicted protein | a | a | a | a | a |
| gi|219122576 | Predicted protein | a | a | a | a | p |
| gi|219125971 | Predicted protein | a | a | a | a | a |
| gi|219127502 | Predicted protein | a | a | a | a | p |
| gi|219127794 | Predicted protein | a | a | a | a | a |
| gi|223999577 | Predicted protein | a | a | a | a | p |
| gi|224001572 | Predicted protein | a | a | a | a | p |
| gi|224009558 | Predicted protein | a | a | a | a | p |
| gi|224009598 | Predicted protein | a | a | a | a | p |
| gi|224014369 | Predicted protein | a | a | a | a | p |
| gi|219116685 | Probable carboxypeptidase; Cxp (ISS) | a | a | a | a | p |
| gi|219125127 | Probable dioxygenase | p | a | a | a | p |
| gi|219109664 | Protein disulfide‐isomerase‐like protein EhSep2 | a | a | a | a | a |
| gi|219113095 | Putative aspartate aminotransferase;38163‐36256 (At1g80360) (Putative | a | a | a | a | p |
| gi|219126206 | Putative digalactosyldiacylglycerol synthase (Os02g0539100 protein) | a | a | a | a | p |
| gi|224012669 | Putative DnaJ (Os05g0543700 protein) | a | a | a | p | a |
| gi|223996253 | Putative enzyme | a | a | a | a | a |
| gi|219115978 | Putative histidine decarboxylase(Serine decarboxylase) | a | a | a | a | p |
| gi|224006580 | Putative histidine decarboxylase(Serine decarboxylase) | a | a | a | a | p |
| gi|219111703 | Putative isochorismatase | a | a | a | a | a |
| gi|223998911 | Putative isochorismatase | a | a | a | a | a |
| gi|219129442 | Putative mitochondrial carrier protein | a | a | a | a | a |
| gi|219110759 | Putative mono‐or diacylglycerol acyltransferase (Os06g0326700 protein) | a | a | a | a | p |
| gi|224011052 | Putative mono‐or diacylglycerol acyltransferase (Os06g0326700 protein) | p | p | a | a | p |
| gi|219125253 | Putative oxigenase (ISS) | a | a | a | a | p |
| gi|219122374 | Putative Reg receptor (Putative uncharacterized protein) | a | a | a | a | p |
| gi|219128329 | Putative uncharacterized protein At3g50685 | a | a | a | a | a |
| gi|219116304 | Putative uncharacterized protein At4g22830/T12H17_220 (Putative | a | a | a | a | p |
| gi|219117235 | Putative uncharacterized protein CBG01120 | a | a | a | a | p |
| gi|219125837 | Putative uncharacterized protein | a | a | a | a | a |
| gi|223995061 | Putative uncharacterized protein | a | a | a | a | p |
| gi|224000848 | Putative uncharacterized protein | a | a | a | a | p |
| gi|223993711 | Putative uncharacterized protein di33 | a | a | a | a | p |
| gi|219128212 | Putative uncharacterized protein F15B8.20 | a | a | a | a | a |
| gi|219129089 | Putative uncharacterized protein F21F14.40 (AT3g61870/F21F14_40) | a | a | a | a | p |
| gi|224000157 | Putative uncharacterized protein F21F14.40 (AT3g61870/F21F14_40) | a | a | a | a | p |
| gi|224001310 | Putative uncharacterized protein | a | a | a | a | p |
| gi|219122551 | Putative uncharacterized protein OSJNBa0009L15.2 (Os05g0358400 | p | p | a | a | a |
| gi|224006305 | Putative uncharacterized protein OSJNBa0009L15.2 (Os05g0358400 | p | p | a | a | a |
| gi|219113251 | Putative uncharacterized protein OSJNBb0081B07.22 (Os03g0822200 | a | a | a | a | p |
| gi|223997414 | Putative uncharacterized protein OSJNBb0081B07.22 (Os03g0822200 | a | a | a | a | p |
| gi|224005240 | Putative uncharacterized protein OSJNBb0081B07.22 (Os03g0822200 | a | a | a | a | p |
| gi|219110441 | Putative uncharacterized protein | p | p | a | a | a |
| gi|219115976 | Putative uncharacterized protein | a | a | a | a | p |
| gi|219116326 | Putative uncharacterized protein | a | a | a | a | p |
| gi|219117125 | Putative uncharacterized protein | a | a | a | a | p |
| gi|219117355 | Putative uncharacterized protein | a | a | a | a | a |
| gi|219118345 | Putative uncharacterized protein | a | a | a | a | a |
| gi|219118645 | Putative uncharacterized protein | a | a | a | a | p |
| gi|219119254 | Putative uncharacterized protein | a | a | a | a | p |
| gi|219121453 | Putative uncharacterized protein | a | a | a | a | p |
| gi|219122175 | Putative uncharacterized protein | a | a | a | a | a |
| gi|219122177 | Putative uncharacterized protein | a | a | a | a | a |
| gi|219126312 | Putative uncharacterized protein | p | a | a | a | p |
| gi|219127880 | Putative uncharacterized protein | a | a | a | a | p |
| gi|219127900 | Putative uncharacterized protein | a | a | a | a | a |
| gi|219128829 | Putative uncharacterized protein | a | a | a | a | p |
| gi|219129244 | Putative uncharacterized protein | p | p | a | p | p |
| gi|223992735 | Putative uncharacterized protein | a | a | a | a | p |
| gi|223995299 | Putative uncharacterized protein | a | a | a | a | p |
| gi|224000786 | Putative uncharacterized protein | a | a | a | a | a |
| gi|224001336 | Putative uncharacterized protein | p | p | p | a | p |
| gi|224001748 | Putative uncharacterized protein | a | a | a | a | p |
| gi|224003423 | Putative uncharacterized protein | a | a | a | a | p |
| gi|224003733 | Putative uncharacterized protein | p | p | a | p | p |
| gi|224006548 | Putative uncharacterized protein | a | a | a | a | a |
| gi|224013724 | Putative uncharacterized protein | a | a | a | a | p |
| gi|224014276 | Putative uncharacterized protein | a | a | a | a | a |
| gi|224014982 | Putative uncharacterized protein | a | a | a | a | p |
| gi|223994903 | Putative uncharacterized protein T17F3.13 (Putative uncharacterized | a | a | a | a | p |
| gi|219126992 | Pyruvate Pi dikinase regulatory protein | a | a | a | a | a |
| gi|224003105 | Retinoblastoma‐binding protein 9(RBBP‐9) (B5T overexpressed gene | a | a | a | a | a |
| gi|219119347 | Root border cell‐specific protein(ISS) | a | a | a | a | p |
| gi|224002090 | SET domain‐containing protein (ISS) | a | a | a | a | p |
| gi|219116062 | SET domain‐containing protein (ISS) | a | a | a | a | p |
| gi|219115321 | Similar to polyhydroxyalkanoate depolymerase | a | a | a | a | a |
| gi|219118652 | Similarity to Serine/arginine‐rich protein (Putative serine/arginine‐ rich | p | p | a | a | a |
| gi|219124722 | Sodium/bile acid cotransporter 7‐B(Na(+)/bile acid cotransporter 7‐B) | a | a | a | a | p |
| gi|219124720 | Sodium/bile acid cotransporter 7(Na(+)/bile acid cotransporter 7) (Solute | a | a | a | a | p |
| gi|224002088 | Sulfotransferase:Tetratricopeptide TPR_1:Tetratricopeptide | a | a | a | a | a |
| gi|219117457 | T22N4.8 protein (At3g01290)(Hypersensitive‐induced response protein) | a | a | a | a | p |
| gi|219116733 | UPF0060 membrane protein yfjF | a | a | a | a | a |
| gi|219119979 | UPF0187 protein At2g45870,chloroplast precursor | a | a | a | a | a |
| gi|219110743 | Xanthine/uracil/vitamin C permease | a | a | a | a | a |
| gi|224007735 | Ycf53 protein | a | a | a | a | p |
| gi|219110367 | Zeaxanthin epoxidase (Fragment) | a | a | a | a | p |
| gi|219115143 | Zeaxanthin epoxidase | a | a | a | a | p |
| gi|224003755 | Zeaxanthin epoxidase | a | a | a | a | p |
| gi|223995267 | Zeaxanthin epoxidase | a | a | a | a | p |
| gi|219126299 | Zgc:136770 | a | a | a | a | p |
