# Supplementary material for: Gene Gain and Loss during Evolution of Obligate Parasitism in the White Rust Pathogen of Arabidopsis thaliana
Source: PLoS Biol. 2011 Jul 5;9(7):e1001094. doi: 10.1371/journal.pbio.1001094 (PMC3130010; doi:10.1371/journal.pbio.1001094)
Supplement: Table S13 — Molecular divergence of A. laibachii based on all orthologous genes. (DOC) [file pbio.1001094.s023.doc]

|  | *Phytophthora sojae/*  *Albugo laibachii* | *Phytophthora infestans/*  *Albugo laibachii* | *Pythium ultimum/*  *Albugo laibachii* | *Hyaloperonospora arabidopsidis/*  *Albugo laibachii* | *Plasmodium falciparum/*  *Albugo laibachii* | *Thaloasiosira pseudonana/*  *Albugo laibachii* | *Phaeodactylum tricornutum/*  *Albugo laibachii* | *Chlamydomonas reinhardtii/*  *Albugo laibachii* |
| --- | --- | --- | --- | --- | --- | --- | --- | --- |
| Total orthologous pairs | 5433 | 5824 | 5910 | 4826 | 1444 | 2596 | 2541 | 2369 |
| Orthologous pairs, single copy orthologs | 4255 | 4527 | 4899 | 3454 | 1094 | 1941 | 1951 | 1747 |
| Mean amino acid identity (%) for all orthologous pairs (1 to 1 orthologs) with gaps | 41.5 | 42.1 | 42 | 39.5 | 27.8 | 31.2 | 31.7 | 30.6 |
| Mean amino acid identity (%) for all orthologous pairs (1 to 1 orthologs)  excluding alignment gaps | 52.2 | 51.6 | 52.1 | 50.5 | 37.1 | 43.8 | 42.8 | 42 |

|  | *Ectocarpus siliculosus/*  *Albugo laibachii* | *Thaloasiosira pseudonana****/*** *Phaeodactylum tricornutum* | *Saccharomyces cerevisiae/ Debaryomyces hansenii* | *Homo sapiens/*  *Takifugu rubripes* | *Hyaloperonospora arabidopsidis/*  *Phytophthora infestans* | *Pythium ultimum/ Hyaloperonospora arabidopsidis* | *Pythium ultimum/*  *Phytophthora infestans* |  |
| --- | --- | --- | --- | --- | --- | --- | --- | --- |
| Total orthologous pairs | 3469 | 5631 | 3635 | 8877 | 6610 | 6096 | 8588 |  |
| Orthologous pairs, single copy orthologs | 2714 | 5095 | 3136 | 6325 | 5293 | 4909 | 7376 |  |
| Mean amino acid identity (%) for all orthologous pairs (1 to 1 orthologs) with gaps | 30.9 | 41.1 | 40 | 51.1 | 59.6 | 47.3 | 50.5 |  |
| Mean amino acid identity (%) for all orthologous pairs (1 to 1 orthologs)  excluding alignment gaps | 41.6 | 53.1 | 45.9 | 62.7 | 73.3 | 59.8 | 62.1 |  |
