# Supplementary material for: Gene Gain and Loss during Evolution of Obligate Parasitism in the White Rust Pathogen of Arabidopsis thaliana
Source: PLoS Biol. 2011 Jul 5;9(7):e1001094. doi: 10.1371/journal.pbio.1001094 (PMC3130010; doi:10.1371/journal.pbio.1001094)
Supplement: Table S14 — Molecular divergence of A. laibachii based on core eukaryotic gene pairs. (DOC) [file pbio.1001094.s024.doc]

|  | *Hyaloperonospora arabidopsidis/*  *Phytophthora infestans* | *Phytophthora sojae/*  *Albugo laibachii* | *Phytophthora infestans/*  *Albugo laibachii* | *Thaloasiosira pseudonana****/***  *Phaeodactylum tricornutum* | *Pythium ultimum/*  *Albugo laibachii* | *Hyaloperonospora arabidopsidis/*  *Albugo laibachii* | *Plasmodium falciparum/*  *Albugo laibachii* | *Saccharomyces cerevisiae/*  *Debaryomyces hansenii* |
| --- | --- | --- | --- | --- | --- | --- | --- | --- |
| Core subset*  (1 to 1 orthologs) | 730 | 744 | 737 | 899 | 779 | 599 | 636 | 1044 |
| Mean amino acid identity (%) for the core orthologous pairs, gaps counted | 70.2 | 57.1 | 57.2 | 54 | 57.4 | 51.2 | 30 | 47.6 |
| Mean amino acid identity (%) for the core orthologous pairs, excluding gaps | 82.1 | 66.3 | 65.6 | 64.2 | 66.2 | 62.2 | 39.4 | 52.6 |

|  | *Thaloasiosira pseudonana/*  *Albugo laibachii* | *Phaeodactylum tricornutum/*  *Albugo laibachii* | *Chlamydomonas reinhardtii/*  *Albugo laibachii* | *Ectocarpus siliculosus/*  *Albugo laibachii* | *Homo sapiens/*  *Takifugu rubripes* | *Pythium ultimum/ Hyaloperonospora arabidopsidis* | *Pythium ultimum/*  *Phytophthora infestans* |
| --- | --- | --- | --- | --- | --- | --- | --- |
| Core subset*  (1 to 1 orthologs) | 688 | 706 | 657 | 782 | 888 | 731 | 917 |
| Mean amino acid identity (%) for the core orthologous pairs, gaps counted | 38.2 | 38 | 36.1 | 38.6 | 62 | 60.3 | 67.2 |
| Mean amino acid identity (%) for the core orthologous pairs, excluding gaps | 50.4 | 49.3 | 47 | 48.8 | 72 | 71.5 | 76.1 |
