# Supplementary material for: Gene Gain and Loss during Evolution of Obligate Parasitism in the White Rust Pathogen of Arabidopsis thaliana
Source: PLoS Biol. 2011 Jul 5;9(7):e1001094. doi: 10.1371/journal.pbio.1001094 (PMC3130010; doi:10.1371/journal.pbio.1001094)
Supplement: Table S15 — Presence and absence of important metabolic enzymes. Red indicates absence and green indicates presence of genes. Genes present were annotated or validated in each organism. Remarkable is the absence of all molybdopterin biosynthesis genes, and enzymes using the cofactor, in A. laibachii and Pl. falciparum. P. infestans and H. arabidopsidis each lack one of the molybdopterin biosynthesis enzymes but contain molybdopterin-dependent enzymes, which might indicate that other enzymes can compensate for the missing step; in case of B73, the missing enzyme might be replaced by a multifunctional Cnx1 or by high concentrations of Mo inside the cell [95]. (DOC) [file pbio.1001094.s025.doc]

|  | *P. falciparum* | *T. pseudonana* | *A. laibachii* | *P. ultimum* | *P. infestans* | *H. arabidopsidis* |
| --- | --- | --- | --- | --- | --- | --- |
| Potential secreted invertase |  |  | AlNc14C8G1068.1 | PYU1_T009242 (partial protein) | PITG_14237T0 | 814664 |
| Hexose transporter | PFB0210c | 269158 | AlNc14C908G12621.1 | PYU1_T010964 | PITG_16383T0 | 806682 |
| Molybdopterin biosynthesis | | | | | | |
| Cnx2 homolog |  | 39516 |  | PYU1_T003071 | PITG_10601T0 | 813499 |
| Cnx3 homolog |  | 9118 |  | PYU1_T008905 |  | 806007 |
| Cnx6 homolog |  | 38193 |  | PYU1_T003371 | PITG_11178T0 | 804342 |
| Cnx1 homolog |  | 32229 |  | PYU1_T014760 | PITG_12540T0 | 810610 |
| B73; molybdenum ion binding |  | 22780 |  | PYU1_T014760 | PITG_12540T0 |  |
| Molybdenum dependant enzyme | | | | | | |
| sulfite oxidase |  | 263844 |  | PYU1_T014111 | PITG_12342T0 | 812582 |
| nitrate reductase |  | 25299 |  | PYU1_T000379 | PITG_13012T0 |  |
| Thiamine biosynthesis | | | | | | |
| Phosphomethyl-pyrimidine kinase | PFE1030c | 262964 |  | PYU1_T007670 |  |  |
| Thiamine-phosphate pyro-phosphorylase | PFF0680c |  |  | PYU1_T005231 |  |  |
