# Supplementary material for: Gene Gain and Loss during Evolution of Obligate Parasitism in the White Rust Pathogen of Arabidopsis thaliana
Source: PLoS Biol. 2011 Jul 5;9(7):e1001094. doi: 10.1371/journal.pbio.1001094 (PMC3130010; doi:10.1371/journal.pbio.1001094)
Supplement: Table S16 — List of all annotated proteins of A. laibachii that might be associated with pathogenicity. Annotation and identification were done using Pfam and BLASTP against the NCBI database. Localisation was predicted using a local installation of WoLF PSORT [96]. SignalP 3.0 was used for secretion prediction. (DOC) [file pbio.1001094.s026.doc]

| **Genes** | **Annotation** | **GI number (*A. laibachii* genes)** | **Localisation prediction** | **Prediction for signal peptide** |
| --- | --- | --- | --- | --- |
| Proteases | | | | |
| AlNc14C66G4683.1 | aspartic protease | 325184745 | cyto_nucl | no_sp |
| AlNc14C38G3311.1 | aspartic protease PM5 | 325183199 | nucl | no_sp |
| AlNc14C2203G13183.1 | aspartyl protease | 325194580 | extr | 0.999 |
| AlNc14C66G4667.1 | aspartyl protease family A01A | 325184729 | nucl | no_sp |
| AlNc14C186G8327.1 | aspartyl protease family A01B | 325188700 | cyto | no_sp |
| AlNc14C246G9563.1 | aspartyl protease family A01B | 325190084 | nucl | no_sp |
| AlNc14C261G9819.1 | aspartyl protease family A01B | 325190367 | plas | no_sp |
| AlNc14C38G3310.1 | aspartyl protease family A01B | 325183198 | plas | no_sp |
| AlNc14C60G4422.1 | aspartyl protease family A01B | 325184469 | plas | no_sp |
| AlNc14C349G10887.1 | aspartyl protease family A22B | 325191346 | plas | no_sp |
| AlNc14C16G1751.1 | astacin-domain containing protein | 325181458 | nucl | no_sp |
| AlNc14C242G9486.1 | ATP-dependent Clp protease | 325189998 | mito | no_sp |
| AlNc14C153G7575.1 | ATP-dependent Clp protease proteolytic subunit | 325187857 | cysk | no_sp |
| AlNc14C22G2277.1 | ATP-dependent hsl protease ATP-binding subunit hslU | 325182050 | mito | no_sp |
| AlNc14C100G6019.1 | ATP-dependent serine protease | 325186172 | mito | no_sp |
| AlNc14C281G10115.1 | CAAX prenyl protease 1 | 325190708 | mito | no_sp |
| AlNc14C55G4231.1 | CAAX prenyl protease 1 | 325184269 | mito | no_sp |
| AlNc14C72G4932.1 | Calpain family cysteine protease containing protein | 325185014 | plas | no_sp |
| AlNc14C281G10113.1 | cathepsin B, cysteine protease family C01A | 325190706 | E.R. | 0.984 |
| AlNc14C55G4233.1 | cathepsin B, cysteine protease family C01A | 325184271 | E.R. | 0.984 |
| AlNc14C9G1195.1 | cathepsin-like cysteine protease | 325180819 | extr | 1 |
| AlNc14C8G1059.1 | cathepsin, cysteine protease family C01A | 325180654 | mito | no_sp |
| AlNc14C18G1899.1 | cathepsin, cysteine protease family C01A | 325181665 | mito | no_sp |
| AlNc14C1G80.1 | cell division protease ftsH | 325179545 | cyto | no_sp |
| AlNc14C521G12032.1 | cleavage induced serine protease family S33 | 325193007 | mito | 0.97 |
| AlNc14C72G4934.1 | cysteine protease family C01A | 325185016 | extr | 0.985 |
| AlNc14C85G5450.1 | cysteine protease family C01A | 325185566 | extr | 0.95 |
| AlNc14C162G7798.1 | cysteine protease family C01A | 325188106 | extr | 0.975 |
| AlNc14C9G1166.1 | cysteine protease family C02 | 325180791 | nucl | no_sp |
| AlNc14C29G2772.1 | cysteine protease family C26 | 325182602 | nucl | no_sp |
| AlNc14C64G4588.1 | cysteine protease family C54 | 325184648 | extr | no_sp |
| AlNc14C263G9844.1 | Calpain family cysteine protease containing protein | 325190394 | nucl | no_sp |
| AlNc14C266G9895.1 | Calpain family cysteine protease containing protein | 325190448 | nucl | no_sp |
| AlNc14C9G1166.1 | Calpain family cysteine protease containing protein | 325180791 | nucl | no_sp |
| AlNc14C12G1490.1 | Calpain family cysteine protease containing protein | 325181141 | cyto_nucl | no_sp |
| AlNc14C72G4932.1 | Calpain family cysteine protease containing protein | 325185014 | plas | no_sp |
| AlNc14C100G6007.1 | endothelin-converting enzyme, metalloprotease family M13 | 325186160 | nucl | no_sp |
| AlNc14C137G7113.1 | gastricsin, aspartyl protease family A01A | 325187342 | plas | 0.923 |
| AlNc14C78G5151.1 | insulin-degrading-like enzyme, metalloprotease family M16A | 325185246 | nucl | no_sp |
| AlNc14C62G4519.1 | leishmanolysin-like peptidase, metalloprotease family M08 | 325184573 | extr | 0.961 |
| AlNc14C21G2195.1 | metalloprotease family M01 | 325181967 | nucl | no_sp |
| AlNc14C1178G12830.1 | metalloprotease family M01, putative | 325193978 | cysk | no_sp |
| AlNc14C274G10019.1 | metalloprotease family M12A | 325190595 | mito | 0.639 |
| AlNc14C188G8366.1 | metalloprotease family M16C | 325188740 | nucl | no_sp |
| AlNc14C84G5413.1 | metalloprotease family M16C | 325185529 | nucl | no_sp |
| AlNc14C251G9646.1 | metalloprotease family M20D | 325190178 | nucl | no_sp |
| AlNc14C4G593.1 | metalloprotease family M48X | 325180120 | cyto_nucl | no_sp |
| AlNc14C132G6983.1 | metalloprotease family M67A | 325187195 | nucl | no_sp |
| AlNc14C37G3251.1 | metalloprotease family M76 | 325183129 | nucl | no_sp |
| AlNc14C4G621.1 | O-sialoglycoprotein endopeptidase | 325180149 | mito | no_sp |
| AlNc14C128G6852.1 | oligopeptidase B | 325187046 | mito | 0.804 |
| AlNc14C454G11755.1 | OTU-like cysteine protease family protein | 325192674 | nucl | no_sp |
| AlNc14C964G12668.1 | OTU-like cysteine protease family protein | 325193793 | nucl | no_sp |
| AlNc14C6G908.1 | peptidase S41 like | 325180461 | plas | no_sp |
| AlNc14C1G36.1 | pro-apoptotic serine protease nma111-like protein | 325179501 | cyto | no_sp |
| AlNc14C22G2250.1 | pro-apoptotic serine protease nma111-like protein | 325182023 | cyto | no_sp |
| AlNc14C2G306.1 | protease | 325179795 | extr | no_sp |
| AlNc14C137G7138.1 | sentrin-specific protease 7 (similar to) | 325187368 | nucl | no_sp |
| AlNc14C125G6778.1 | serine protease | 325186959 | mito | no_sp |
| AlNc14C45G3693.2 | serine protease family S01A | 325183677 | extr | no_sp |
| AlNc14C685G12401.1 | serine protease family S01B | 325193472 | mito | no_sp |
| AlNc14C2G281.1 | serine protease family S01B | 325179770 | mito | no_sp |
| AlNc14C5G671.1 | serine protease family S01B | 325180203 | nucl | no_sp |
| AlNc14C220G9094.1 | serine protease family S08A | 325189579 | extr | 0.998 |
| AlNc14C28G2701.1 | serine protease family S08A | 325182517 | plas | no_sp |
| AlNc14C137G7116.1 | serine protease family S08A | 325187345 | extr | 0.998 |
| AlNc14C206G8813.1 | serine protease family S09A | 325189243 | mito | no_sp |
| AlNc14C205G8796.1 | serine protease family S09X | 325189225 | nucl | no_sp |
| AlNc14C225G9194.1 | serine protease family S09X | 325189690 | nucl | no_sp |
| AlNc14C267G9906.1 | serine protease family S09X | 325190462 | nucl | no_sp |
| AlNc14C61G4448.1 | serine protease family S09X | 325184498 | mito | no_sp |
| AlNc14C64G4594.1 | serine protease family S09X | 325184654 | nucl | no_sp |
| AlNc14C178G8174.1 | serine protease family S09X | 325188535 | cyto | no_sp |
| AlNc14C156G7657.1 | serine protease family S09X | 325187955 | nucl | no_sp |
| AlNc14C333G10711.1 | serine protease family S10 | 325191143 | extr | 1 |
| AlNc14C342G10802.1 | serine protease family S10 | 325191248 | extr | 0.954 |
| AlNc14C4G542.1 | serine protease family S10 | 325180070 | extr | 0.99 |
| AlNc14C4G550.1 | serine protease family S10 | 325180078 | extr | no_sp |
| AlNc14C437G11633.1 | serine protease family S15 | 325192536 | cyto_nucl | no_sp |
| AlNc14C189G8408.1 | serine protease family S33 | 325188787 | cyto | no_sp |
| AlNc14C204G8772.1 | serine protease family S33 | 325189199 | plas | no_sp |
| AlNc14C212G8939.1 | serine protease family S33 | 325189406 | plas | 0.989 |
| AlNc14C235G9365.1 | serine protease family S33 | 325189866 | cyto | no_sp |
| AlNc14C436G11630.1 | serine protease family S33 | 325192532 | cyto_nucl | no_sp |
| AlNc14C622G12270.1 | serine protease family S33 | 325193323 | plas | no_sp |
| AlNc14C23G2314.1 | serine protease family S33 | 325182098 | mito | no_sp |
| AlNc14C39G3383.1 | serine protease family S33 | 325183271 | mito | no_sp |
| AlNc14C49G3908.1 | serine protease family S33 | 325183917 | mito | no_sp |
| AlNc14C103G6104.1 | serine protease family S33 | 325186266 | plas | no_sp |
| AlNc14C91G5703.1 | serine protease family S54 | 325185827 | plas | no_sp |
| AlNc14C1G91.1 | signal peptide peptidase-like, aspartyl protease family A22B | 325179557 | plas | no_sp |
| AlNc14C91G5704.1 | threonine protease family T01A | 325185828 | cyto | no_sp |
| AlNc14C298G10347.1 | vacuolar-processing enzyme | 325190970 | extr | 1 |
| AlNc14C298G10348.1 | vacuolar-processing enzyme | 325190971 | nucl | no_sp |
| AlNc14C87G5549.1 | xaa-Pro dipeptidase, metalloprotease family M24B | 325185670 | cyto_nucl | no_sp |
| Protease inhibitors | | | | |
| AlNc14C76G5100.1 | Kazal-like serine protease inhibitor | 325185192 | extr | 0.785 |
| AlNc14C177G8157.1 | Kazal-like serine protease inhibitor | 325188512 | extr | 0.995 |
| AlNc14C621G12264.1 | Kazal-like serine protease inhibitor | 325193316 | nucl | no_sp |
| AlNc14C202G8728.1 | cystatin-like cysteine protease inhibitor | 325189152 | cyto | 0.920 |
| Glycosyl hydrolases | | | | |
| AlNc14C181G8226.1 | ER degradation-enhancing alpha-mannosidase-like protein | 325188591 | mito | 0.998 |
| AlNc14C206G8823.1 | glycoside hydrolase GH72 | 325189253 | plas | 0.999 |
| AlNc14C309G10482.1 | beta-mannosidase | 325191783 | mito | no_sp |
| AlNc14C330G10680.1 | betra-glucosidase | 325191519 | mito | 0.999 |
| AlNc14C338G10751.1 | glycoside hydrolase GH6 | 325185569 | cyto_nucl | no_sp |
| AlNc14C453G11745.1 | glucan 1,4-_-glucosidase (best candidate) | 325192664 | plas | 0.994 |
| AlNc14C661G12353.1 | beta-glucosidase | 325193408 | plas | no_sp |
| AlNc14C735G12464.1 | beta-galactosidase | 325193544 | nucl | no_sp |
| AlNc14C1128G12796.1 | ER degradation-enhancing alpha-mannosidase-like protein | 325193942 | cyto | no_sp |
| AlNc14C1177G12829.1 | ER degradation-enhancing alpha-mannosidase-like protein | 325193977 | cysk | no_sp |
| AlNc14C2036G13133.1 | ER degradation-enhancing alpha-mannosidase-like protein | 325194475 | nucl | no_sp |
| AlNc14C2428G13233.1 | ER degradation-enhancing alpha-mannosidase-like protein | 325194724 | cysk | no_sp |
| AlNc14C3644G13352.1 | ER degradation-enhancing alpha-mannosidase-like protein | 325195697 | cysk | no_sp |
| AlNc14C15G1711.1 | glycoside hydrolase GH63 | 325181405 | nucl | no_sp |
| AlNc14C270G9949.1 | endo-1,3(4)-beta-glucanase | 325190508 | extr | no_sp |
| AlNc14C501G11942.1 | endo-Polygalacturonase | 325192898 | cyto | no_sp |
| AlNc14C98G5938.1 | endo-1,3(4)-beta-glucanase | 325186081 | plas | no_sp |
| AlNc14C98G5938.2 | endo-1,3(4)-beta-glucanase | 325186077 | plas | no_sp |
| AlNc14C98G5938.3 | endo-1,3(4)-beta-glucanase | 325186080 | plas | no_sp |
| AlNc14C98G5938.4 | endo-1,3(4)-beta-glucanase | 325186079 | plas | no_sp |
| AlNc14C98G5938.5 | endo-1,3(4)-beta-glucanase | 325186078 | plas | no_sp |
| AlNc14C270G9952.1 | endo-1,3(4)-beta-glucanase | 325190511 | mito | no_sp |
| AlNc14C536G12090.1 | Di-N-acetylchitobiase | 325193085 | pero | no_sp |
| AlNc14C1G41.1 | lysosomal alpha-mannosidase | 325179506 | extr | 0.988 |
| AlNc14C3G399.1 | glucosylceramidase | 325179889 | plas | no_sp |
| AlNc14C3G411.1 | glycoside hydrolase GH72 | 325179901 | extr | 1 |
| AlNc14C12G1414.1 | glycoside hydrolase GH72, GTPase domain | 325181064 | plas | 0.999 |
| AlNc14C12G1414.2 | glycoside hydrolase GH72, GTPase domain | 325181064 | nucl | no_sp |
| AlNc14C12G1414.3 | glycoside hydrolase GH72, GTPase domain | 325181064 | nucl | no_sp |
| AlNc14C8G1064.1 | glycoside hydrolase GH32NC | 325180659 | mito | no_sp |
| AlNc14C8G1068.1 | beta-D-fructofuranosidase (invertase) | 325180663 | extr | 0.74 |
| AlNc14C14G1654.1 | 1,3-beta-glucanosyltransferase | 325181333 | nucl | no_sp |
| AlNc14C16G1761.1 | glycoside hydrolase GH72 | 325181467 | extr | no_sp |
| AlNc14C25G2494.1 | Di-N-acetylchitobiase | 325182296 | cyto | no_sp |
| AlNc14C28G2684.1 | glycoside hydrolase GH6 | 325182499 | mito | 0.997 |
| AlNc14C36G3225.1 | beta-D-galactosidase | 325183103 | nucl | no_sp |
| AlNc14C58G4339.1 | endo-1,3-beta-glucanase, family GH81 | 325184383 | plas | no_sp |
| AlNc14C58G4341.1 | mannosyl-oligosaccharide glucosidase | 325184385 | extr | 0.773 |
| AlNc14C61G4450.1 | glycoside hydrolase GH53 | 325184500 | mito | 0.984 |
| AlNc14C62G4501.1 | endo-1,3(4)-beta-glucanase | 325184554 | plas | no_sp |
| AlNc14C62G4523.1 | polygalacturonase | 325184577 | cyto | no_sp |
| AlNc14C62G4525.1 | endo-polygalacturonase | 325184580 | cyto | no_sp |
| AlNc14C85G5453.1 | cellulose 1,4-_-cellobiosidase | 325185569 | cyto | no_sp |
| AlNc14C88G5610.1 | thioglucosidase, myrosinase; sinigrinase; sinigrase | 325185734 | mito | 0.93 |
| AlNc14C120G6656.1 | glycoside hydrolase GH3 | 325186831 | mito | 0.976 |
| AlNc14C120G6657.1 | _-glucosidase | 325186832 | extr | 0.998 |
| AlNc14C37G3269.1 | endo-1,3(4)-beta-glucanase | 325183152 | mito | no_sp |
| AlNc14C130G6935.1 | exoglucanase 1 | 325187145 | mito | no_sp |
| AlNc14C145G7367.1 | endo-1,3-beta-glucanase | 325187607 | mito | 0.995 |
| AlNc14C162G7796.1 | glycoside hydrolase GH72 | 325188104 | plas | no_sp |
|  |  | 325189221 |  |  |
| AlNc14C205G8792.1 | cutinase | 325184956 | extr | 0.944 |
| AlNc14C71G4881.1 | cutinase | 325192855 | extr | 1 |
| AlNc14C489G11911.1 | pectate lyase | 325188591 | cyto | no_sp |
| Lipases | | | | |
| AlNc14C38G3302.1 | hormone-sensitive lipase | 325183185 | nucl | no_sp |
| AlNc14C100G6016.1 | lipase | 325186169 | plas | 0.999 |
| AlNc14C278G10075.1 | lipase class 3 family protein | 325190663 | extr | no_sp |
| AlNc14C438G11639.1 | lipase class 3 family protein | 325192544 | plas | no_sp |
| AlNc14C7G997.1 | lipase class 3 family protein | 325180590 | nucl | no_sp |
| AlNc14C38G3300.1 | lipase class 3 family protein | 325183183 | plas | no_sp |
| AlNc14C100G6013.1 | lipase class 3 family protein | 325186166 | extr | 0.579 |
| AlNc14C115G6525.1 | lipase class 3 family protein | 325186686 | plas | 0.532 |
| AlNc14C183G8267.1 | Patatin like protein | 325188636 | cyto | no_sp |
| AlNc14C8G1028.1 | Patatin like protein | 325180622 | cyto | no_sp |
| AlNc14C82G5340.1 | Patatin like protein | 325185449 | mito | no_sp |
| AlNc14C156G7653.1 | Patatin like protein | 325187951 | plas | no_sp |
| AlNc14C435G11619.1 | phospholipase A-2-activating protein | 325192519 | nucl | no_sp |
| AlNc14C151G7529.1 | phospholipase A2 | 325187801 | cyto | 0.999 |
| AlNc14C192G8477.1 | phospholipase D | 325188871 | cysk | no_sp |
| AlNc14C265G9874.1 | Phospholipase D | 325190425 | plas | no_sp |
| AlNc14C265G9874.2 | Phospholipase D | 325190425 | plas | no_sp |
| AlNc14C265G9874.3 | Phospholipase D | 325190425 | plas | no_sp |
| AlNc14C265G9874.4 | Phospholipase D | 325190425 | plas | no_sp |
| AlNc14C265G9874.5 | Phospholipase D | 325190425 | plas | no_sp |
| AlNc14C265G9874.6 | Phospholipase D | 325190425 | plas | no_sp |
| AlNc14C265G9874.7 | Phospholipase D | 325190425 | plas | no_sp |
| AlNc14C265G9874.8 | Phospholipase D | 325190425 | plas | no_sp |
| AlNc14C265G9874.9 | Phospholipase D | 325190425 | plas | no_sp |
| AlNc14C265G9874.10 | Phospholipase D | 325190425 | plas | no_sp |
| AlNc14C265G9874.11 | Phospholipase D | 325190425 | plas | no_sp |
| AlNc14C265G9874.12 | Phospholipase D | 325190425 | plas | no_sp |
| AlNc14C265G9874.13 | Phospholipase D | 325190425 | plas | no_sp |
| AlNc14C265G9874.14 | Phospholipase D | 325190425 | plas | no_sp |
| AlNc14C265G9874.15 | Phospholipase D | 325190425 | plas | no_sp |
| AlNc14C265G9874.16 | Phospholipase D | 325190425 | plas | no_sp |
| AlNc14C265G9874.17 | Phospholipase D | 325190425 | plas | no_sp |
| AlNc14C265G9874.18 | Phospholipase D | 325190425 | plas | no_sp |
| AlNc14C265G9874.19 | Phospholipase D | 325190425 | plas | no_sp |
| AlNc14C265G9874.20 | Phospholipase D | 325190425 | plas | no_sp |
| AlNc14C265G9874.21 | Phospholipase D | 325190425 | plas | no_sp |
| AlNc14C265G9874.22 | Phospholipase D | 325190425 | plas | no_sp |
| AlNc14C265G9874.23 | Phospholipase D | 325190425 | plas | no_sp |
| AlNc14C265G9874.24 | Phospholipase D | 325190425 | plas | no_sp |
| AlNc14C265G9874.25 | Phospholipase D | 325190425 | plas | no_sp |
| AlNc14C265G9874.26 | Phospholipase D | 325190425 | plas | no_sp |
| AlNc14C265G9874.27 | Phospholipase D | 325190425 | plas | no_sp |
| AlNc14C265G9874.28 | Phospholipase D | 325190425 | plas | no_sp |
| AlNc14C265G9874.29 | Phospholipase D | 325190425 | plas | no_sp |
| AlNc14C265G9874.30 | Phospholipase D | 325190425 | plas | no_sp |
| AlNc14C265G9874.31 | Phospholipase D | 325190425 | plas | no_sp |
| AlNc14C265G9874.32 | Phospholipase D | 325190425 | plas | no_sp |
| AlNc14C6G881.1 | Phospholipase D | 325180434 | plas | no_sp |
| AlNc14C99G5969.1 | Phospholipase D | 325186118 | plas | no_sp |
| AlNc14C144G7333.1 | Phospholipase D | 325187571 | cysk | no_sp |
| AlNc14C152G7552.1 | phospholipase D | 325187825 | nucl | no_sp |
| AlNc14C199G8647.1 | phospholipase D, Pi-PLD-like-1 | 325189058 | nucl | no_sp |
| AlNc14C107G6266.1 | phospholipase D, Pi-PXPH-PLD | 325186432 | nucl | no_sp |
| AlNc14C199G8645.1 | phospholipase D, Pi-sPLD-like-9 | 325189055 | extr | 0.987 |
| AlNc14C199G8646.1 | phospholipase D, Pi-sPLD-like-9 | 325189057 | extr | 0.964 |
| AlNc14C91G5706.1 | phospholipase, with phosphoinositides binding domain | 325185830 | cyto | no_sp |
| AlNc14C174G8076.1 | sn1-specific diacylglycerol lipase beta | 325188420 | mito | no_sp |
| P450 | | | | |
| AlNc14C22G2282.1 | cytochrome P450 | 325182055 | E.R. | no_sp |
| AlNc14C61G4475.1 | cytochrome P450 | 325184526 | extr | no_sp |
| AlNc14C130G6945.1 | cytochrome P450 | 325187155 | mito | 0.844 |
| ABC transporter | | | | |
| AlNc14C186G8323.1 | ATP-binding cassette sub-family F member 1 | 325188696 | nucl | no_sp |
| AlNc14C189G8394.1 | ATP-binding Cassette (ABC) Superfamily | 325188773 | plas | no_sp |
| AlNc14C192G8473.1 | ATP-binding Cassette (ABC) Superfamily | 325188867 | plas | no_sp |
| AlNc14C216G9009.1 | ATP-binding Cassette (ABC) Superfamily | 325189484 | plas | no_sp |
| AlNc14C220G9092.1 | ATP-binding Cassette (ABC) Superfamily | 325189577 | plas | no_sp |
| AlNc14C235G9378.1 | ATP-binding Cassette (ABC) Superfamily | 325189879 | plas | no_sp |
| AlNc14C258G9762.1 | ATP-binding Cassette (ABC) Superfamily (molybdenum transport protein) | 325190305 | mito | no_sp |
| AlNc14C268G9930.1 | ATP-binding Cassette (ABC) Superfamily | 325190487 | plas | 1 |
| AlNc14C283G10139.1 | ATP-binding Cassette (ABC) Superfamily | 325190735 | plas | no_sp |
| AlNc14C296G10298.1 | ATP-binding Cassette (ABC) Superfamily | 325190919 | plas | no_sp |
| AlNc14C381G11221.1 | ATP-binding Cassette (ABC) Superfamily | 325192051 | plas | 1 |
| AlNc14C394G11310.1 | ATP-binding Cassette (ABC) Superfamily | 325192149 | plas | no_sp |
| AlNc14C4224G13377.1 | ATP-binding Cassette (ABC) Superfamily | 325196267 | cysk | no_sp |
| AlNc14C3G412.1 | ATP-binding Cassette (ABC) Superfamily | 325179902 | plas | 0.987 |
| AlNc14C6G830.1 | ATP-binding Cassette (ABC) Superfamily | 325180376 | plas | no_sp |
| AlNc14C6G864.1 | ATP-binding Cassette (ABC) Superfamily | 325180417 | nucl | no_sp |
| AlNc14C11G1323.1 | ATP-binding Cassette (ABC) Superfamily | 325180970 | plas | no_sp |
| AlNc14C23G2370.1 | ATP-binding Cassette (ABC) Superfamily | 325182160 | cysk | no_sp |
| AlNc14C23G2383.1 | ATP-binding Cassette (ABC) Superfamily | 325182173 | plas | no_sp |
| AlNc14C24G2409.1 | ATP-binding Cassette (ABC) Superfamily | 325182199 | plas | no_sp |
| AlNc14C32G2951.1 | ATP-binding Cassette (ABC) Superfamily | 325182799 | plas | no_sp |
| AlNc14C35G3135.1 | ATP-binding Cassette (ABC) Superfamily | 325183010 | plas | no_sp |
| AlNc14C47G3806.1 | ATP-binding Cassette (ABC) Superfamily | 325183807 | plas | no_sp |
| AlNc14C64G4562.1 | ATP-binding Cassette (ABC) Superfamily | 325184621 | cysk | no_sp |
| AlNc14C64G4564.1 | ATP-binding Cassette (ABC) Superfamily | 325184623 | plas | no_sp |
| AlNc14C64G4604.1 | ATP-binding Cassette (ABC) Superfamily | 325184664 | plas | no_sp |
| AlNc14C115G6526.1 | ATP-binding Cassette (ABC) Superfamily | 325186687 | plas | no_sp |
| AlNc14C37G3266.1 | ATP-binding Cassette (ABC) Superfamily | 325183145 | cyto_nucl | no_sp |
| AlNc14C37G3266.2 | ATP-binding Cassette (ABC) Superfamily | 325183147 | nucl | no_sp |
| AlNc14C37G3266.3 | ATP-binding Cassette (ABC) Superfamily | 325183146 | cyto_nucl | no_sp |
| AlNc14C37G3266.4 | ATP-binding Cassette (ABC) Superfamily | 325183144 | cyto_nucl | no_sp |
| AlNc14C37G3266.5 | ATP-binding Cassette (ABC) Superfamily | 325183148 | nucl | no_sp |
| AlNc14C37G3266.6 | ATP-binding Cassette (ABC) Superfamily | 325183149 | cyto_nucl | no_sp |
| AlNc14C132G6985.1 | ATP-binding Cassette (ABC) Superfamily | 325187197 | plas | no_sp |
| AlNc14C138G7162.1 | ATP-binding Cassette (ABC) Superfamily (molybdenum transport protein) | 325187392 | mito | no_sp |
| AlNc14C144G7337.1 | ATP-binding Cassette (ABC) Superfamily | 325187575 | plas | no_sp |
| AlNc14C157G7673.1 | ATP-binding Cassette (ABC) Superfamily | 325187972 | plas | no_sp |
| AlNc14C159G7728.1 | ATP-binding Cassette (ABC) Superfamily | 325188031 | plas | no_sp |
| AlNc14C166G7876.1 | ATP-binding Cassette (ABC) Superfamily | 325188192 | mito | no_sp |
| AlNc14C174G8094.1 | ATP-binding Cassette (ABC) Superfamily | 325188441 | mito | no_sp |
| AlNc14C178G8166.1 | ATP-binding Cassette (ABC) Superfamily | 325188522 | plas | no_sp |
| Potential PAMPS | | | | |
| AlNc14C26G2564.1 | elicitin-like protein SOL7 | 325182370 | nucl | 0.999 |
| AlNc14C34G3088.1 | elicitin-like protein | 325182958 | cyto | no_sp |
| AlNc14C159G7739.1 | elicitin-like protein | 325188042 | nucl | no_sp |
| AlNc14C6G846.1 | cellulose binding elicitor lectin (CBEL) | 325180392 | extr | 0.996 |
| AlNc14C67G4718.1 | cellulose binding elicitor lectin (CBEL) | 325184781 | extr | 0.995 |
| AlNc14C67G4719.1 | cellulose binding elicitor lectin (CBEL) | 325184782 | mito | 0.987 |
| AlNc14C311G10490.1 | lectin | 325191762 | nucl | no_sp |
| AlNc14C511G11992.1 | lectin | 325192957 | extr | 0.989 |
| AlNc14C12G1421.1 | lectin | 325181071 | mito | 0.952 |
| AlNc14C2G338.1 | transglutaminase elicitor M81C | 325179827 | extr | 0.998 |
| AlNc14C2G340.1 | elicitor-like transglutaminase M81-like protein (with PEP13) | 325179829 | mito | 0.914 |
| AlNc14C5G717.1 | elicitor-like mating protein M81 | 325180253 | extr | 0.746 |
| AlNc14C5G727.1 | glycoprotein elicitor precursor | 325180263 | extr | 0.989 |
| AlNc14C5G736.1 | glycoprotein elicitor precursor | 325180276 | extr | 0.771 |
