# Supplementary material for: Gene Gain and Loss during Evolution of Obligate Parasitism in the White Rust Pathogen of Arabidopsis thaliana
Source: PLoS Biol. 2011 Jul 5;9(7):e1001094. doi: 10.1371/journal.pbio.1001094 (PMC3130010; doi:10.1371/journal.pbio.1001094)
Supplement: Table S17 — Intraspecies comparison between Nc14 and Em1. All genes, genes with a predicted secretion signal peptide and without a TM domain, genes representing KOGs, or genes carrying a CHXC, RXLR, or RXLQ motif were compared. The second column in the table indicates heterozygosity (het) within Nc14; the third column indicates heterozygous positions within Em1 (green) or homozygous (hom) SNPs between Nc14 and Em1 (blue). The fourth column shows Em1-specific heterozygous positions or SNPs corrected against Nc14 heterozygous positions carrying the same nucleotide in one of the haplotypes. Frequencies of non-synonymous and synonymous mutations (darker coloured fields, mutations per 100 bp) are almost balanced in the all-gene and KOG gene comparisons, while a comparison of all secreted proteins indicates a 3∶1 ratio (non-synonymous∶synonymous). RXLRs and, particularly, RXLQs show an imbalance (∼2∶1), with high variation due to the small sample size. CHXCs, with a ratio of ∼5∶1, show a significant imbalance in the comparison between Nc14 and Em1. Considering total number and percentage of genes with a ratio of non-synonymous/synonymous <1 or >1 (light-coloured fields), only KOG genes show a significantly higher number of genes with a value <1, while all other classes show more genes with a value >1. (DOC) [file pbio.1001094.s027.doc]

|  | Nc14 🡪 Nc14 | Nc14 🡪 Em1 | Nc14 🡪 Em1 (Em1 unique) |
| --- | --- | --- | --- |
| All genes het:  Synonymous  Non-synonymous  all | 0.0118  0.0234  0.0352 | 0.0211  0.0293  0.0504 | 0.0187  0.0247  0.0434 |
| non-syn. / syn. < 1 | 322  (2.5%) | 1216  (9.3%) | 1189  (9.1%) |
| non-syn. / syn. > 1 | 899  (6.9%) | 1929  (14.8%) | 1796  (13.8%) |
| All genes hom:  Synonymous  Non-synonymous  all | - | 0.0118  0.0181  0.0299 | 0.0090  0.0129  0.0219 |
| non-syn. / syn. < 1 | - | 726  (5.6%) | 664  (5.1) |
| non-syn. / syn. > 1 | - | 1189  (9.1%) | 970  (7.4%) |
| Genes with sec. signal het:  Synonymous  Non-synonymous  All | 0.0090  0.0302  0.0391 | 0.0210  0.0721  0.0930 | 0.0166  0.0570  0.0735 |
| non-syn. / syn. < 1 | 8  (1.4%) | 33  (5.9%) | 30  (5.4%) |
| non-syn. / syn. het > 1 | 19  (3.4%) | 87  (15.6%) | 84  (15.1%) |
| Genes with sec. signal hom:  Synonymous  Non-synonymous  All | - | 0.0123  0.0365  0.0488 | 0.0180  0.0284  0.0386 |
| non-syn. / syn. hom < 1 | - | 20  (3.6%) | 20  (3.6%) |
| non-syn. / syn. hom > 1 | - | 72  (12.9%) | 52  (9.3%) |
| KOG genes het:  Synonymous  Non-synonymous  All | 0.0033  0.0033  0.0066 | 0.0196  0.0126  0.0322 | 0.0191  0.0126  0.0317 |
| non-syn. / syn. het < 1 | 4  (1.0%) | 60  (15.8%) | 59  (15.5%) |
| non-syn. / syn. het > 1 | 8  (2.1%) | 36  (9.5%) | 37  (9.7%) |
| KOG genes hom:  Synonymous  Non-synonymous  All | - | 0.0099  0.0062  0.0161 | 0.0090  0.0050  0.0140 |
| non-syn. / syn. hom < 1 | - | 30  (7.9%) | 30  (7.9%) |
| non-syn. / syn. hom > 1 | - | 17  (4.5%) | 15  (3.9%) |
| CHXC candidates het:  Synonymous  Non-synonymous  All | 0.0291  0.1853  0.2144 | 0.0400  0.2144  0.2543 | 0.0145  0.0763  0.0908 |
| non-syn. / syn. het < 1 | 0  (0%) | 1  (3.5%) | 1  (3.5%) |
| non-syn. / syn. het > 1 | 3  (10.4%) | 8  (27.6%) | 7  (24.1%) |
| CHXC candidates hom:  Synonymous  Non-synonymous  All | - | 0.0000  0.0327  0.0327 | 0.0000  0.0254  0.0254 |
| non-syn. / syn. hom < 1 | - | 0  (0%) | 0  (0%) |
| non-syn. / syn. hom > 1 | - | 4  (13.7%) | 2  (6.8%) |
| RXLR candidates het:  Synonymous  Non-synonymous  All | 0.0208  0.0934  0.1141 | 0.0156  0.0311  0.0467 | 0.0156  0.0259  0.0415 |
| non-syn. / syn. het < 1 | 0  (0.0%) | 1  (4.0%) | 1  (4.0%) |
| non-syn. / syn. het > 1 | 2  (8.0%) | 3  (12.0%) | 3  (12.0%) |
| RXLR candidates hom:  Synonymous  Non-synonymous  All | - | 0.0415  0.3113  0.0726 | 0.0363  0.0259  0.0623 |
| non-syn. / syn. hom < 1 | - | 2  (8.0%) | 2  (8.0%) |
| non-syn. / syn. hom > 1 | - | 1  (4.0%) | 1  (4.0%) |
| RXLQ candidates het:  Synonymous  Non-synonymous  All | 0.0000  0.0000  0.0000 | 0.0000  0.0311  0.0311 | 0.0000  0.0311  0.0311 |
| non-syn. / syn. het < 1 | 0  (0.0%) | 0  (0.0%) | 0  (0.0%) |
| non-syn. / syn. het > 1 | 0  (0.0%) | 5  (21.7%) | 5  (21.7%) |
| RXLQ candidates:  Synonymous  Non-synonymous  All | - | 0.0116  0.8507  0.0967 | 0.0116  0.8507  0.0967 |
| non-syn. / syn. hom < 1 | - | 0  (0.0%) | 0  (0.0%) |
| non-syn. / syn. hom > 1 | - | 3  (13.0%) | 3  (13.0%) |
