# Supplementary material for: Gene Gain and Loss during Evolution of Obligate Parasitism in the White Rust Pathogen of Arabidopsis thaliana
Source: PLoS Biol. 2011 Jul 5;9(7):e1001094. doi: 10.1371/journal.pbio.1001094 (PMC3130010; doi:10.1371/journal.pbio.1001094)
Supplement: Table S18 — CHXC and CXHC candidate genes. This table gives an overview of all predicted CHXC (white background) and CXHC (grey background) candidates from various species. The first column of the table indicates name of CHXC or CXHC candidates used for the phylogenetic analyses (Figure 9). The second column indicates the species name, and the third column indicates the locus tag within the corresponding genome. The fourth column shows the best BLAST hit against the NCBI nr database with an e-value<10−50. The last column indicates the accession number of the best hit. (DOC) [file pbio.1001094.s028.doc]

| **Tree name** | **Organism** | **Locus tag (corresonding genome)** | **Best blast hit NCBI nr (<1E-50)** | **reference number** |
| --- | --- | --- | --- | --- |
| CXHC_Ath01 | A. thaliana | AT2G28040.1 | aspartyl protease family protein [Arabidopsis thaliana] | NP_180371.2 |
| CXHC_Ath02 | A. thaliana | AT2G28010.1 | aspartyl protease family protein [Arabidopsis thaliana] | NP_180368.1 |
| CXHC_Ath03 | A. thaliana | AT2G47470.1 | UNE5 (UNFERTILIZED EMBRYO SAC 5); protein disulfide isomerase [Arabidopsis thaliana] | NP_182269.1 |
| CXHC_Ath04 | A. thaliana | AT2G47470.3 | UNE5 (UNFERTILIZED EMBRYO SAC 5); protein disulfide isomerase [Arabidopsis thaliana] | NP_001031555.1 |
| CXHC_Ath05 | A. thaliana | AT2G47470.4 | UNE5 (UNFERTILIZED EMBRYO SAC 5); protein disulfide isomerase [Arabidopsis thaliana] | NP_001078074.1 |
| CXHC_Ath06 | A. thaliana | AT4G09120.1 | zinc finger (C3HC4-type RING finger) family protein [Arabidopsis thaliana] | NP_192651.1 |
| CXHC_Ath07 | A. thaliana | AT4G37810.1 | unknown protein [Arabidopsis thaliana] | NP_680774.1 |
| CXHC_Ath08 | A. thaliana | AT1G21750.1 | ATPDIL1-1 (PDI-LIKE 1-1); protein disulfide isomerase [Arabidopsis thaliana] | NP_173594.1 |
| CXHC_Ath09 | A. thaliana | AT1G04980.1 | ATPDIL2-2 (PDI-LIKE 2-2); protein disulfide isomerase [Arabidopsis thaliana] | NP_171990.3 |
| CXHC_Ath10 | A. thaliana | AT1G07960.2 | ATPDIL5-1 (PDI-like 5-1) [Arabidopsis thaliana] | NP_172274.1 |
| CXHC_Ath11 | A. thaliana | AT1G19090.1 | RKF2 (receptor-like serine/threonine kinase 2) [Arabidopsis thaliana] | NP_564071.3 |
| CXHC_Ath12 | A. thaliana | AT1G35620.1 | ATPDIL5-2 (PDI-LIKE 5-2); protein disulfide isomerase [Arabidopsis thaliana] | NP_564462.1 |
| CXHC_Ath13 | A. thaliana | AT1G21750.2 | ATPDIL1-1 (PDI-LIKE 1-1); protein disulfide isomerase [Arabidopsis thaliana] | NP_849696.1 |
| CXHC_Ath14 | A. thaliana | AT1G07960.3 | ATPDIL5-1 (PDI-like 5-1) [Arabidopsis thaliana] | NP_172274.1 |
| CXHC_Ath15 | A. thaliana | AT1G07960.1 | ATPDIL5-1 (PDI-like 5-1) [Arabidopsis thaliana] | NP_172274.1 |
| CXHC_Ath16 | A. thaliana | AT1G73603.1 | - | - |
| CXHC_Ath17 | A. thaliana | AT1G73607.1 | - | - |
| CXHC_Ath18 | A. thaliana | AT1G49715.1 | - | - |
| CXHC_Ath19 | A. thaliana | AT1G13607.1 | - | - |
| CXHC_Ath20 | A. thaliana | AT1G77510.1 | ATPDIL1-2 (PDI-LIKE 1-2); protein disulfide isomerase [Arabidopsis thaliana] | NP_177875.1 |
| CXHC_Ath21 | A. thaliana | AT2G32920.1 | ATPDIL2-3 (PDI-LIKE 2-3); protein disulfide isomerase [Arabidopsis thaliana] | NP_180851.1 |
| CXHC_Ath22 | A. thaliana | AT2G47470.2 | hypothetical protein ARALYDRAFT_904222 [Arabidopsis lyrata subsp. lyrata] | XP_002880305.1 |
| CHXC_Ath01 | A. thaliana | AT2G22807.1 | - | - |
| CHXC_Ath02 | A. thaliana | AT3G04370.1 | PDLP4 (PLASMODESMATA-LOCATED PROTEIN 4) [Arabidopsis thaliana] | NP_187087.2 |
| CHXC_Ath03 | A. thaliana | AT3G21970.1 | receptor-like protein kinase-related [Arabidopsis thaliana] | NP_188835.1 |
| CHXC_Ath04 | A. thaliana | AT3G59390.2 | unknown protein [Arabidopsis thaliana] | NP_191498.2 |
| CHXC_Ath05 | A. thaliana | AT3G29040.1 | protein serine/threonine kinase [Arabidopsis thaliana] | NP_189547.2 |
| CHXC_Ath06 | A. thaliana | AT3G59390.1 | unknown protein [Arabidopsis thaliana] | NP_191498.2 |
| CHXC_Ath07 | A. thaliana | AT3G04370.2 | PDLP4 (PLASMODESMATA-LOCATED PROTEIN 4) [Arabidopsis thaliana] | NP_001030631.1 |
| CHXC_Ath08 | A. thaliana | AT4G23310.1 | receptor-like protein kinase, putative [Arabidopsis thaliana] | NP_194062.1 |
| CHXC_Ath09 | A. thaliana | AT5G10310.1 | unknown protein [Arabidopsis thaliana] | NP_196593.1 |
| CHXC_Ath10 | A. thaliana | AT5G50345.1 | - | - |
| CHXC_Ath11 | A. thaliana | AT1G34040.1 | alliinase family protein [Arabidopsis thaliana] | NP_174666.1 |
| CXHC_Cla01 | C. reinhardtii | 190396 | hypothetical protein [Chlamydomonas reinhardtii] | dbjBAF46293.1 |
| CXHC_Cla02 | C. reinhardtii | 195895 | protein disulfide isomerase 1 [Chlamydomonas reinhardtii] | XP_001701755.1 |
| CXHC_Cla03 | C. reinhardtii | 182974 | protein disulfide isomerase [Chlamydomonas reinhardtii] | XP_001690462.1 |
| CXHC_Cla04 | C. reinhardtii | 188533 | hypothetical protein CHLREDRAFT_188533 [Chlamydomonas reinhardtii] | XP_001690657.1 |
| CXHC_Cla05 | C. reinhardtii | 189940 | protein disulfide isomerase [Chlamydomonas reinhardtii] | XP_001689618.1 |
| CXHC_Cla06 | C. reinhardtii | 187017 | hypothetical protein CHLREDRAFT_187017 [Chlamydomonas reinhardtii] | XP_001696470.1 |
| CXHC_Cla07 | C. reinhardtii | 185378 | protein disulfide isomerase [Chlamydomonas reinhardtii] | XP_001699351.1 |
| CXHC_Cla08 | C. reinhardtii | 178513 | predicted protein [Chlamydomonas reinhardtii] | XP_001700415.1 |
| CXHC_Cla09 | C. reinhardtii | 106059 | hypothetical zinc-dependent DNA-binding protein [Chlamydomonas reinhardtii] | XP_001698247.1 |
| CXHC_Cla10 | C. reinhardtii | 183905 | predicted protein [Chlamydomonas reinhardtii] | XP_001693302.1 |
| CHXC_Cla01 | C. reinhardtii | 191786 | predicted protein [Chlamydomonas reinhardtii] | XP_001695530.1 |
| CHXC_Cla02 | C. reinhardtii | 182461 | gamma-glutamyl hydrolase [Chlamydomonas reinhardtii] | XP_001700978.1 |
| CHXC_Cla03 | C. reinhardtii | 190396 | hypothetical protein [Chlamydomonas reinhardtii] | dbjBAF46293.1 |
| CHXC_Cla04 | C. reinhardtii | 187751 | cullin-related protein [Chlamydomonas reinhardtii] | XP_001698597.1 |
| CHXC_Cla05 | C. reinhardtii | 184380 | hypothetical protein CHLREDRAFT_184380 [Chlamydomonas reinhardtii] | XP_001695907.1 |
| CHXC_Cla06 | C. reinhardtii | 184669 | - | - |
| CHXC_Cla07 | C. reinhardtii | 183905 | predicted protein [Chlamydomonas reinhardtii] | XP_001693302.1 |
| CXHC_Pin01 | P. infestans | 14917T0 | thioredoxin-like protein [Phytophthora infestans T30-4] | XP_002899091.1 |
| CXHC_Pin02 | P. infestans | 01791T0 | protein disulfide-isomerase, putative [Phytophthora infestans T30-4] | XP_002908400.1 |
| CXHC_Pin03 | P. infestans | 21378T0 | protein disulfide-isomerase, putative [Phytophthora infestans T30-4] | XP_002895135.1 |
| CXHC_Pin04 | P. infestans | 21551T0 | thioredoxin-like protein [Phytophthora infestans T30-4] | XP_002894823.1 |
| CXHC_Pin05 | P. infestans | 21552T0 | sulfhydryl oxidase, putative [Phytophthora infestans T30-4] | XP_002894824.1 |
| CXHC_Pin06 | P. infestans | 03694T0 | disulfide-isomerase, putative [Phytophthora infestans T30-4] | XP_002906746.1 |
| CXHC_Pin07 | P. infestans | 03909T0 | thioredoxin-like protein [Phytophthora infestans T30-4] | XP_002906944.1 |
| CXHC_Pin08 | P. infestans | 03910T0 | sulfhydryl oxidase, putative [Phytophthora infestans T30-4] | XP_002906945.1 |
| CXHC_Pin09 | P. infestans | 06657T0 | thioredoxin-like protein [Phytophthora infestans T30-4] | XP_002998735.1 |
| CXHC_Pin10 | P. infestans | 07843T0 | protein disulfide-isomerase, putative [Phytophthora infestans T30-4] | XP_002904062.1 |
| CXHC_Pin11 | P. infestans | 10972T0 | thioredoxin-like protein [Phytophthora infestans T30-4] | XP_002901765.1 |
| CHXC_Pin01 | P. infestans | 12806T0 | phospholipase D, Pi-sPLD-like-12 [Phytophthora infestans T30-4] | XP_002900188.1 |
| CHXC_Pin02 | P. infestans | 12809T0 | phospholipase D, Pi-sPLD-like-11 [Phytophthora infestans T30-4] | XP_002900190.1 |
| CHXC_Pin03 | P. infestans | 00141T0 | conserved hypothetical protein [Phytophthora infestans T30-4] | XP_002908768.1 |
| CHXC_Pin04 | P. infestans | 22814T0 | conserved hypothetical protein [Phytophthora infestans T30-4] | XP_002904456.1 |
| CHXC_Pin05 | P. infestans | 10572T0 | phospholipase D, Pi-sPLD-like-9 [Phytophthora infestans T30-4] | XP_002902345.1 |
| CHXC_Pfa01 | P. infestans | PF11_466 | transporter, putative [Plasmodium falciparum 3D7] | XP_001348133.2 |
| CXHC_Pfa01 | P. infestans | PF13_272 | thioredoxin-related protein, putative [Plasmodium falciparum 3D7] | XP_001350237.1 |
| CXHC_Pfa02 | P. infestans | MAL8P1.17 | protein disulfide isomerase [Plasmodium falciparum 3D7] | XP_002808883.1 |
| CXHC_Pul01 | P. infestans | T011861 | thioredoxin-like protein [Phytophthora infestans T30-4] | XP_002899091.1 |
| CXHC_Pul02 | P. infestans | T014812 | thioredoxin-like protein [Phytophthora infestans T30-4] | XP_002998735.1 |
| CXHC_Pul03 | P. infestans | T011498 | NUK7 [Phytophthora infestans] | gbABM05490.1 |
| CXHC_Pul04 | P. infestans | T005696 | protein disulfide-isomerase [Phytophthora infestans] | gbAAN31493.1 |
| CXHC_Pul05 | P. infestans | T013575 | sulfhydryl oxidase, putative [Phytophthora infestans T30-4] | XP_002906945.1 |
| CHXC_Pul01 | P. infestans | T006605 | phospholipase D, Pi-sPLD-like-9 [Phytophthora infestans T30-4] | XP_002902345.1 |
| CHXC_Pul02 | P. infestans | T006606 | phospholipase D, Pi-sPLD-like-9 [Phytophthora infestans T30-4] | XP_002902345.1 |
| CHXC_Pul03 | P. infestans | T011130 | palmitoyltransferase, putative [Phytophthora infestans T30-4] | XP_002904306.1 |
| CHXC_Pul04 | P. infestans | T008181 | - | - |
| CXHC_Tps01 | T. pseudonana | 10867 | predicted protein [Thalassiosira pseudonana CCMP1335] | XP_002295405.1 |
| CXHC_Tps02 | T. pseudonana | 21965 | predicted protein [Thalassiosira pseudonana CCMP1335] | XP_002289024.1 |
| CXHC_Tps03 | T. pseudonana | 24931 | predicted protein [Thalassiosira pseudonana CCMP1335] | XP_002293826.1 |
| CXHC_Tps04 | T. pseudonana | 24932 | predicted protein [Thalassiosira pseudonana CCMP1335] | XP_002293999.1 |
| CXHC_Tps05 | T. pseudonana | 33929 | predicted protein [Thalassiosira pseudonana CCMP1335] | XP_002289309.1 |
| CXHC_Tps06 | T. pseudonana | 37338 | predicted protein [Thalassiosira pseudonana CCMP1335] | XP_002293992.1 |
| CHXC_Tps01 | T. pseudonana | 1080 | predicted protein [Thalassiosira pseudonana CCMP1335] | XP_002286620.1 |
| CHXC_Tps02 | T. pseudonana | 2224 | predicted protein [Thalassiosira pseudonana CCMP1335] | XP_002287199.1 |
| CXHC_Hpa01 | H. arabidopsidis | 811307 | disulfide-isomerase, putative [Phytophthora infestans T30-4] | XP_002906746.1 |
| CXHC_Hpa02 | H. arabidopsidis | 803550 | NUK7 [Phytophthora infestans] | gbABM05490.1 |
| CXHC_Hpa03 | H. arabidopsidis | 804133 | protein disulfide-isomerase, putative [Phytophthora infestans T30-4]gb|EEY61483.1| | XP_002908400.1 |
| CXHC_Hpa04 | H. arabidopsidis | 805750 | sulfhydryl oxidase, putative [Phytophthora infestans T30-4] | XP_002906945.1 |
| CXHC_Hpa05 | H. arabidopsidis | 806291 | thioredoxin-like protein [Phytophthora infestans T30-4] | XP_002899091.1 |
| CXHC_Hpa06 | H. arabidopsidis | 809065 | protein disulfide-isomerase, putative [Phytophthora infestans T30-4] | XP_002895135.1 |
| CXHC_Hpa07 | H. arabidopsidis | 809066 | protein disulfide-isomerase, putative [Phytophthora infestans T30-4] | XP_002895135.1 |
| CXHC_Hpa08 | H. arabidopsidis | 809133 | thioredoxin-like protein [Phytophthora infestans T30-4] | XP_002998735.1 |
| CXHC_Hpa09 | H. arabidopsidis | 809756 | conserved hypothetical protein [Phytophthora infestans T30-4] | XP_002902542.1 |
| CXHC_Hpa10 | H. arabidopsidis | 807762 | - | - |
| CHXC_Hpa01 | H. arabidopsidis | 811327 | - | - |
| CHXC_Hpa02 | H. arabidopsidis | 801554 | - | - |
| CHXC_Hpa03 | H. arabidopsidis | 810578 | - | - |
| CXHC_Al01 | A. laibachii | AlNc14C67G4708.1 | - | - |
| CXHC_Al02 | A. laibachii | AlNc14C67G4708.2 | - | - |
| CXHC_Al03 | A. laibachii | AlNc14C228G9250.1 | - | - |
| CXHC_Al04 | A. laibachii | AlNc14C1G182.1 | - | - |
| CXHC_Al05 | A. laibachii | AlNc14C2G235.1 | disulfide-isomerase, putative [Phytophthora infestans T30-4] | XP_002906746.1 |
| CXHC_Al06 | A. laibachii | AlNc14C7G976.1 | - | - |
| CXHC_Al07 | A. laibachii | AlNc14C21G2151.1 | - | - |
| CXHC_Al08 | A. laibachii | AlNc14C65G4629.1 | sulfhydryl oxidase, putative [Phytophthora infestans T30-4] | XP_002906945.1 |
| CXHC_Al09 | A. laibachii | AlNc14C361G10994.1 | - | - |
| CXHC_Al10 | A. laibachii | AlNc14C398G11350.1 | protein disulfide-isomerase, putative [Phytophthora infestans T30-4] | XP_002895135.1 |
| CXHC_Al11 | A. laibachii | AlNc14C417G11499.1 | - | - |
| CXHC_Al12 | A. laibachii | AlNc14C442G11682.1 | thioredoxin-like protein [Phytophthora infestans T30-4] | XP_002998735.1 |
| CXHC_Al13 | A. laibachii | AlNc14C845G12567.1 | conserved hypothetical protein [Phytophthora infestans T30-4] | XP_002997428.1 |
| CXHC_Al14 | A. laibachii | AlNc14C1806G13074.1 | - | - |
| CHXC_Al01 | A. laibachii | AlNc14C9G1189.1 | HECT E3 ubiquitin ligase, putative [Phytophthora infestans T30-4] | XP_002907421.1 |
| CHXC_Al02 | A. laibachii | AlNc14C69G4807.1 | - | - |
| CHXC_Al04 | A. laibachii | AlNc14C109G6324.1 | - | - |
| CHXC_Al05 | A. laibachii | AlNc14C83G13418.1 | - | - |
| CHXC_Al06 | A. laibachii | AlNc14C199G8645.1 | phospholipase D, Pi-sPLD-like-9 [Phytophthora infestans T30-4] | XP_002902345.1 |
| CHXC_Al07 | A. laibachii | AlNc14C191G8449.1 | - |  |
| CHXC_Al08 | A. laibachii | AlNc14C92G5741.1 | - | - |
| CHXC_Al09 | A. laibachii | AlNc14C832G12555.1 | - | - |
| CHXC_Al13 | A. laibachii | AlNc14C235G9380.1 | - | - |
| CHXC_Al14 | A. laibachii | AlNc14C286G10177.1 | - | - |
| CHXC_Al15 | A. laibachii | AlNc14C7G1010.1 | - | - |
| CHXC_Al16 | A. laibachii | AlNc14C26G2552.1 | - | - |
| CHXC_Al18 | A. laibachii | AlNc14C255G9708.1 | - | - |
| CHXC_Al19 | A. laibachii | AlNc14C293G10262.1 | - | - |
| CHXC_Al20 | A. laibachii | AlNc14C302G10393.1 | - | - |
| CHXC_Al21 | A. laibachii | AlNc14C140G13405.1 | - | - |
| CHXC_Al22 | A. laibachii | AlNc14C407G13406.1 | - | - |
| CHXC_Al23 | A. laibachii | AlNc14C2245G130407.1 | - | - |
| CHXC_Al24 | A. laibachii | AlNc14C429G13408.1 | - | - |
| CHXC_Al25 | A. laibachii | AlNc14C158G13409.1 | - | - |
| CHXC_Al26 | A. laibachii | AlNc14C906G13410.1 | - | - |
| CHXC_Al27 | A. laibachii | AlNc14C180G13411.1 | - | - |
| CHXC_Al29 | A. laibachii | AlNc14C313G13412.1 | - | - |
| CHXC_Al30 | A. laibachii | AlNc14C74G13413.1 | - | - |
| CHXC_Al31 | A. laibachii | AlNc14C1700G13414.1 | - | - |
| CHXC_Al32 | A. laibachii | AlNc14C444G13415.1 | - | - |
| CHXC_Al33 | A. laibachii | AlNc14C125G13416.1 | - | - |
| CHXC_Al34 | A. laibachii | AlNc14C825G13417.1 | - | - |
| CXHC_Cme01 | C. merolae | CMK015C | - | - |
| CXHC_Cme02 | C. merolae | CMN111C | - | - |
| CXHC_Cme03 | C. merolae | CMC180C | PREDICTED: similar to protein disulfide isomerase-associated 3 [Ciona intestinalis] | XP_002126714.1 |
| CHXC_Cme01 | C. merolae | CMK015C | - | - |
| CXHC_Ect01 | E. siliculosus | Esi0000_0146 | protein disulfide-isomerase [Ectocarpus siliculosus] | embCBN76521.1 |
| CXHC_Ect02 | E. siliculosus | Esi0164_0066 | protein disulfide isomerase [Ectocarpus siliculosus] | embCBJ29913.1 |
| CXHC_Ect03 | E. siliculosus | Esi0213_0049 | EsV-1-166 [Ectocarpus siliculosus] | embCBJ30739.1 |
| CXHC_Ect04 | E. siliculosus | Esi0383_0005 | EsV-1-166 [Ectocarpus siliculosus] | embCBJ32867.1 |
| CXHC_Ect05 | E. siliculosus | Esi0402_0019 | protein disulfide isomerase [Ectocarpus siliculosus] | embCBJ33005.1 |
| CXHC_Ect06 | E. siliculosus | Esi0538_0004 | disulfide-isomerase A6 precursor [Ectocarpus siliculosus] | embCBN79876.1 |
| CXHC_Ect07 | E. siliculosus | Esi0018_0136 | conserved unknown protein [Ectocarpus siliculosus] | embCBJ26043.1 |
| CXHC_Ect08 | E. siliculosus | Esi0030_0031 | Thioredoxin family protein [Ectocarpus siliculosus] | embCBN74544.1 |
| CXHC_Ect09 | E. siliculosus | Esi0041_0044 | EsV-1-166 [Ectocarpus siliculosus] | embCBN74730.1 |
| CXHC_Ect10 | E. siliculosus | Esi0052_0152 | EsV-1-166 [Ectocarpus siliculosus] | embCBN80457.1 |
| CXHC_Ect11 | E. siliculosus | Esi0062_0043 | protein disulfide isomerase [Ectocarpus siliculosus] | embCBN77707.1 |
| CXHC_Ect12 | E. siliculosus | Esi0063_0121 | hypothetical protein [Ectocarpus siliculosus] | embCBJ27276.1 |
| CXHC_Ect13 | E. siliculosus | Esi0094_0038 | conserved unknown protein [Ectocarpus siliculosus] | embCBJ28171.1 |
| CXHC_Ect14 | E. siliculosus | Esi0004_0034 | EsV-1-166 [Ectocarpus siliculosus] | embCBN77502.1 |
| CXHC_Ect15 | E. siliculosus | Esi0004_0035 | EsV-1-166 [Ectocarpus siliculosus] | embCBN77503.1 |
| CXHC_Ect16 | E. siliculosus | Esi0114_0016 | EsV-1-166 [Ectocarpus siliculosus] | embCBN75518.1 |
| CXHC_Ect17 | E. siliculosus | Esi0114_0036 | EsV-1-166 [Ectocarpus siliculosus] | embCBN75520.1 |
| CXHC_Ect18 | E. siliculosus | Esi0004_0156 | EsV-1-166 [Ectocarpus siliculosus] | embCBN77564.1 |
| CXHC_Ect19 | E. siliculosus | Esi0005_0003 | conserved unknown protein [Ectocarpus siliculosus] | embCBN78223.1 |
| CXHC_Ect20 | E. siliculosus | Esi0155_0059 | Protein disulfide-isomerase fusion protein [Ectocarpus siliculosus] | embCBN78923.1 |
| CXHC_Ect21 | E. siliculosus | Esi0016_0047 | - | - |
| CHXC_Ect01 | E. siliculosus | Esi0367_0011 | conserved unknown protein [Ectocarpus siliculosus] | embCBJ32777.1 |
| CHXC_Ect02 | E. siliculosus | Esi0032_0090 | imm upregulated 12 [Ectocarpus siliculosus] | embCBJ26374.1 |
| CHXC_Ect03 | E. siliculosus | Esi0070_0125 | conserved unknown protein [Ectocarpus siliculosus] | embCBN75161.1 |
| CXHC_Spr01 | S. parasitica | 10287T0 | - | - |
| CXHC_Spr02 | S. parasitica | 11026T0 | NUK7 [Phytophthora infestans] | gbABM05490.1 |
| CXHC_Spr03 | S. parasitica | 11118T0 | - | - |
| CXHC_Spr04 | S. parasitica | 11830T0 | - | - |
| CXHC_Spr05 | S. parasitica | 01190T0 | long-chain-fatty-acid-CoA ligase, putative [Phytophthora infestans T30-4] | XP_002907969.1 |
| CXHC_Spr06 | S. parasitica | 15004T0 | - | - |
| CXHC_Spr07 | S. parasitica | 01635T0 | - | - |
| CXHC_Spr08 | S. parasitica | 17312T0 | - | - |
| CXHC_Spr09 | S. parasitica | 17890T0 | - | - |
| CXHC_Spr10 | S. parasitica | 02085T0 | gastricsin, aspartyl protease family A01A, putative [Phytophthora infestans T30-4] | XP_002902303.1 |
| CXHC_Spr11 | S. parasitica | 02145T0 | PREDICTED: similar to protein disulfide isomerase [Hydra magnipapillata] | XP_002159276.1 |
| CXHC_Spr12 | S. parasitica | 02637T0 | thioredoxin-like protein [Phytophthora infestans T30-4] | XP_002901765.1 |
| CXHC_Spr13 | S. parasitica | 03243T0 | - | - |
| CXHC_Spr14 | S. parasitica | 04464T0 | protein disulfide-isomerase, putative [Phytophthora infestans T30-4] | XP_002895135.1 |
| CXHC_Spr15 | S. parasitica | 06029T0 | disulfide-isomerase, putative [Phytophthora infestans T30-4] | XP_002906746.1 |
| CXHC_Spr16 | S. parasitica | 06772T0 | PREDICTED: similar to protein disulfide isomerase [Hydra magnipapillata] | XP_002159276.1 |
| CXHC_Spr17 | S. parasitica | 09907T0 | PREDICTED: similar to protein disulfide isomerase [Hydra magnipapillata] | XP_002159276.1 |
| CXHC_Spr18 | S. parasitica | 09910T0 | - | - |
| CXHC_Spr19 | S. parasitica | 09913T0 | - | - |
| CHXC_Spr01 | S. parasitica | 10287T0 | - | - |
| CHXC_Spr02 | S. parasitica | 11563T0 | HECT E3 ubiquitin ligase, putative [Phytophthora infestans T30-4] | XP_002901655.1 |
| CHXC_Spr03 | S. parasitica | 12666T0 | phospholipase D, Pi-sPLD-like-9 [Phytophthora infestans T30-4] | XP_002902345.1 |
| CHXC_Spr04 | S. parasitica | 12667T0 | phospholipase D, Pi-sPLD-like-9 [Phytophthora infestans T30-4] | XP_002902345.1 |
| CHXC_Spr05 | S. parasitica | 13369T0 | aspartyl protease family A01B, putative [Phytophthora infestans T30-4] | XP_002907533.1 |
| CHXC_Spr06 | S. parasitica | 15975T0 | - | - |
| CHXC_Spr07 | S. parasitica | 16173T0 | twinkle protein, putative [Phytophthora infestans T30-4] | XP_002900786.1 |
| CHXC_Spr08 | S. parasitica | 18119T0 | - | - |
| CHXC_Spr09 | S. parasitica | 18676T0 | - | - |
| CHXC_Spr10 | S. parasitica | 00019T0 | - | - |
| CHXC_Spr11 | S. parasitica | 03745T0 | - | - |
| CHXC_Spr12 | S. parasitica | 03991T0 | - | - |
| CHXC_Spr13 | S. parasitica | 00549T0 | conserved hypothetical protein [Phytophthora infestans T30-4] | XP_002997428.1 |
| CHXC_Spr14 | S. parasitica | 09889T0 | putative lipoprotein [Myxococcus xanthus DK 1622] | YP_633198.1 |
| CXHC_Vca01 | V. carteri | 99167 | - | - |
| CXHC_Vca02 | V. carteri | 105867 | hypothetical protein VOLCADRAFT_105867 [Volvox carteri f. nagariensis] | XP_002953269.1 |
| CXHC_Vca03 | V. carteri | 104135 | hypothetical protein VOLCADRAFT_104135 [Volvox carteri f. nagariensis] | XP_002948959.1 |
| CXHC_Vca04 | V. carteri | 75277 | hypothetical protein VOLCADRAFT_75277 [Volvox carteri f. nagariensis] | XP_002952241.1 |
| CXHC_Vca05 | V. carteri | 62315 | hypothetical protein VOLCADRAFT_62315 [Volvox carteri f. nagariensis] | XP_002952260.1 |
| CXHC_Vca06 | V. carteri | 90262 | hypothetical protein VOLCADRAFT_90262 [Volvox carteri f. nagariensis] | XP_002949836.1 |
| CXHC_Vca07 | V. carteri | 77404 | protein disulfide isomerase 1 [Volvox carteri f. nagariensis] | XP_002957079.1 |
| CXHC_Vca08 | V. carteri | 106773 | hypothetical protein VOLCADRAFT_106773 [Volvox carteri f. nagariensis] | XP_002955374.1 |
| CHXC_Vca01 | V. carteri | 99349 | hypothetical protein VOLCADRAFT_99349 [Volvox carteri f. nagariensis] | XP_002958168.1 |
| CHXC_Vca02 | V. carteri | 106317 | hypothetical protein VOLCADRAFT_106317 [Volvox carteri f. nagariensis] | XP_002954301.1 |
| CHXC_Vca03 | V. carteri | 100038 | hypothetical protein VOLCADRAFT_100038 [Volvox carteri f. nagariensis] | XP_002958742.1 |
| CHXC_Vca04 | V. carteri | 104152 | hypothetical protein VOLCADRAFT_104152 [Volvox carteri f. nagariensis] | XP_002948981.1 |
| CHXC_Vca05 | V. carteri | 89086 | hypothetical protein VOLCADRAFT_89086 [Volvox carteri f. nagariensis] | XP_002948821.1 |
